# Nanobody‐Engineered Biohybrid Bacteria Targeting Gastrointestinal Cancers Induce Robust STING‐Mediated Anti‐Tumor Immunity

**DOI:** 10.1002/advs.202401905

**Published:** 2024-06-18

**Authors:** Xiaolong Xu, Youbin Ding, Yafang Dong, Haitao Yuan, Peng Xia, Chengming Qu, Jingbo Ma, Huifang Wang, Xiaodong Zhang, Liang Zhao, Zhijie Li, Zhen Liang, Jigang Wang

**Affiliations:** ^1^ Department of Geriatrics and Shenzhen Clinical Research Centre for Geriatrics, Department of Urology Shenzhen People's Hospital (The First Affiliated Hospital, Southern University of Science and Technology The Second Clinical Medical College Jinan University) Shenzhen Guangdong 518020 China; ^2^ Integrated Chinese and Western Medicine Postdoctoral Research Station Jinan University Guangzhou 510632 China; ^3^ Department of Medical Imaging The Third Affiliated Hospital Southern Medical University (Academy of Orthopedics Guangdong Province) Guangzhou 510515 China; ^4^ Department of Hepatobiliary & Pancreatic Surgery Zhongnan Hospital of Wuhan University Wuhan Hubei 430071 China; ^5^ Department of Pathology Shunde Hospital, Southern Medical University (The First People's Hospital of Shunde) Foshan 528308 China; ^6^ Department of Pathology & Guangdong Province Key Laboratory of Molecular Tumor Pathology, School of Basic Medical Sciences Southern Medical University Guangzhou 510515 China; ^7^ Department of Oncology The Affiliated Hospital of Southwest Medical University Luzhou Sichuan 646000 China; ^8^ Department of Traditional Chinese Medicine and School of Pharmaceutical Sciences Southern Medical University Guangzhou 510515 China; ^9^ State Key Laboratory for Quality Ensurance and Sustainable Use of Dao‐di Herbs, Artemisinin Research Center, and Institute of Chinese Materia Medica China Academy of Chinese Medical Sciences Beijing 100700 China; ^10^ State Key Laboratory of Antiviral Drugs School of Pharmacy Henan University Kaifeng 475004 China

**Keywords:** bacteria, immunotherapy, nanobody, photothermal therapy, STING

## Abstract

Bacteria can be utilized for cancer therapy owing to their preferential colonization at tumor sites. However, unmodified non‐pathogenic bacteria carry potential risks due to their non‐specific targeting effects, and their anti‐tumor activity is limited when used as monotherapy. In this study, a biohybrid‐engineered bacterial system comprising non‐pathogenic MG1655 bacteria modified with CDH17 nanobodies on their surface and conjugated with photosensitizer croconium (CR) molecules is developed. The resultant biohybrid bacteria can efficiently home to CDH17‐positive tumors, including gastric, pancreatic, and colorectal cancers, and significantly suppress tumor growth upon irradiation. More importantly, biohybrid bacteria‐mediated photothermal therapy (PTT) induced abundant macrophage infiltration in a syngeneic murine colorectal model. Further, that the STING pathway is activated in tumor macrophages by the released bacterial nucleic acid after PTT is revealed, leading to the production of type I interferons. The addition of CD47 nanobody but not PD‐1 antibody to the PTT regimen can eradicate the tumors and extend survival. This results indicate that bacteria endowed with tumor‐specific selectivity and coupled with photothermal payloads can serve as an innovative strategy for low‐immunogenicity cancers. This strategy can potentially reprogram the tumor microenvironment by inducing macrophage infiltration and enhancing the efficacy of immunotherapy targeting macrophages.

## Graphical abstract

This work developed a biohybrid bacterial system through genetic engineering and biomaterial hybridization in which CDH17 nanobodies are engineered onto the bacteria for tumor targeting and a photothermal material croconium (CR) dye is conjugated on bacteria for PTT induction. When combined with checkpoint blockade CD47 nanobody, the biohybrid bacteria successfully eradicated the tumors through STING activation, and considerably extended survival of the mice.

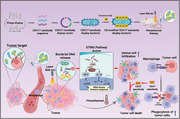

## 1 Introduction

Bacterial cancer therapy can be traced back to William B. Coley's pioneering work of injecting bacteria into cancer patients in 1891 and tumor elimination was observed in some patients, which served as one of the earliest examples of cancer immunotherapy. However, at that time, the immunostimulatory role of bacteria toward tumors was not understood.^[^ ^1^ ^]^ On the other hand, the systemic administration of natural bacteria could potentially result in severe infection and septic shock, as observed in some patients treated by William B. Coley with bacteria.^[^ ^1a^ ^]^ With a better understanding of the tumor microenvironment (TME) and the development of DNA recombinant technology, cancer bacterial therapy has garnered more attention, and numerous preclinical studies have been conducted in recent years.^[^ ^2^ ^]^ The virulence of bacteria, which is highly associated with toxicity, can be attenuated through the modification of corresponding genes, such as lipopolysaccharide (LPS)‐related genes. The intrinsic characteristic of anaerobic bacteria, which preferentially grow and proliferate in hypoxic or necrotic lesions, allows them to accumulate significantly more (≈10 000‐fold) in tumor tissues with the hypoxic and immunosuppressive microenvironment compared to normal organs.^[^ ^3^ ^]^ However, the phase I clinical trial with facultative anaerobe *Salmonella typhimurium* VNP2009, an attenuated strain with high safety, unfortunately failed to show therapeutic effects in metastatic melanoma patients. The trial also revealed dose‐dependent adverse effects and insufficient accumulation in tumor tissues.^[^ ^1^, ^4^ ^]^ These results indicate that the genetic attenuation strategy and passive targeting property of bacteria are insufficient to achieve tumor regression and improve therapeutic index. Although anaerobic bacteria can proliferate in tumor sites and be eliminated by the immune system in normal organs, the temporal distribution of bacteria in normal organs upon systemic administration can still lead to intolerable side effects. Moreover, some facultative anaerobes can survive and proliferate in normal organs with oxygenated microenvironments.^[^ ^3^, ^5^ ^]^ Therefore, it is necessary to develop strategies to improve tumor‐targeting selectivity of bacteria without compromising therapeutic efficacy.

Intratumoral administration is a commonly used route in preclinical studies to demonstrate the potential applications of bacterial therapy for cancer.^[^ ^2^, ^6^ ^]^ This approach avoids the potential safety issues and significantly elevates the local concentration of bacteria. Although this method is feasible for tumors accessible through local injection, it is impractical for inaccessible tumors. Another alternative strategy is to engineer the surface of bacteria using genetic manipulation technologies to confer bacteria with tumor‐specific tropism. This approach can greatly enhance the accumulation of bacteria in tumor sites while limiting their distribution in normal organs. Various molecules have been engineered onto the bacterial surface to increase their tropism toward tumor cells, such as tumor‐targeting peptide arginine–glycine–aspartate (RGD) bound to α_v_β_3_ integrins and antibody fragments targeting tumor antigens like CD20 and cancer‐associated carcinoembryonic antigen (CEA).^[^ ^7^ ^]^ Evidently, the efficient clinical translation of bacterial cancer therapy requires identification of new molecules specifically expressed in tumor tissues and development of novel modalities that target these specific markers. These advancements will enable the design of effective delivery strategies for bacterial therapy in cancer treatment. Irrespective of chemotherapy, targeted therapy, or immunotherapy, monotherapy is normally less effective in clinical settings. Attenuated bacteria alone are inadequate to fully control tumor progression. As live microorganisms, bacteria possess remarkable capabilities for delivering therapeutic payloads. Through genetic modifications and synthetic biological strategies, bacteria can deliver various therapeutic agents, including cytotoxic proteins, DNA, RNA, and small molecules.^[^ ^6^, ^8^ ^]^ Moreover, surface modification of bacteria with diverse materials represents an important strategy to enhance therapeutic efficacy through the combination of bacteria therapy with material properties, such as cytotoxicity, photothermal and photodynamic effects, catalytic activity.^[^ ^6^, ^9^ ^]^ Bacteria modified with payloads or materials can synergistically suppress tumors through more complex mechanisms relative to bacteria alone, primarily involving the priming of the immune system, such as the activation of innate immunity through microbial‐associated molecular patterns (MAMPs) and pattern‐recognition receptors (PRRs).^[^ ^2^, ^5^, ^10^ ^]^

Although various preclinical studies on bacterial cancer therapy have been conducted in different animal models with promising results and bacteria are also a potent and versatile system for cancer therapy, successful clinical translation remains unseen. Most preclinical studies focused solely on one aspect of bacteria for cancer therapy. For instance, harnessing the intrinsic tumor‐homing ability of bacteria delivers payloads or produces therapeutic agents in situ, which overlooks the potential systemic side effects associated with the distribution of bacteria in normal organs. On the other hand, some studies solely enhanced the tumor selectivity of bacteria through surface engineering without fully utilizing their potent capacity for payload delivery or production. Few studies have consolidated active tumor targeting and payload delivery into one bacterial system to achieve efficient tumor suppression and even imaging and diagnosis. Additionally, a deeper understanding of the underlying mechanisms of action for distinct bacterial platforms is crucial for achieving successful clinical translation.

**Scheme 1:**
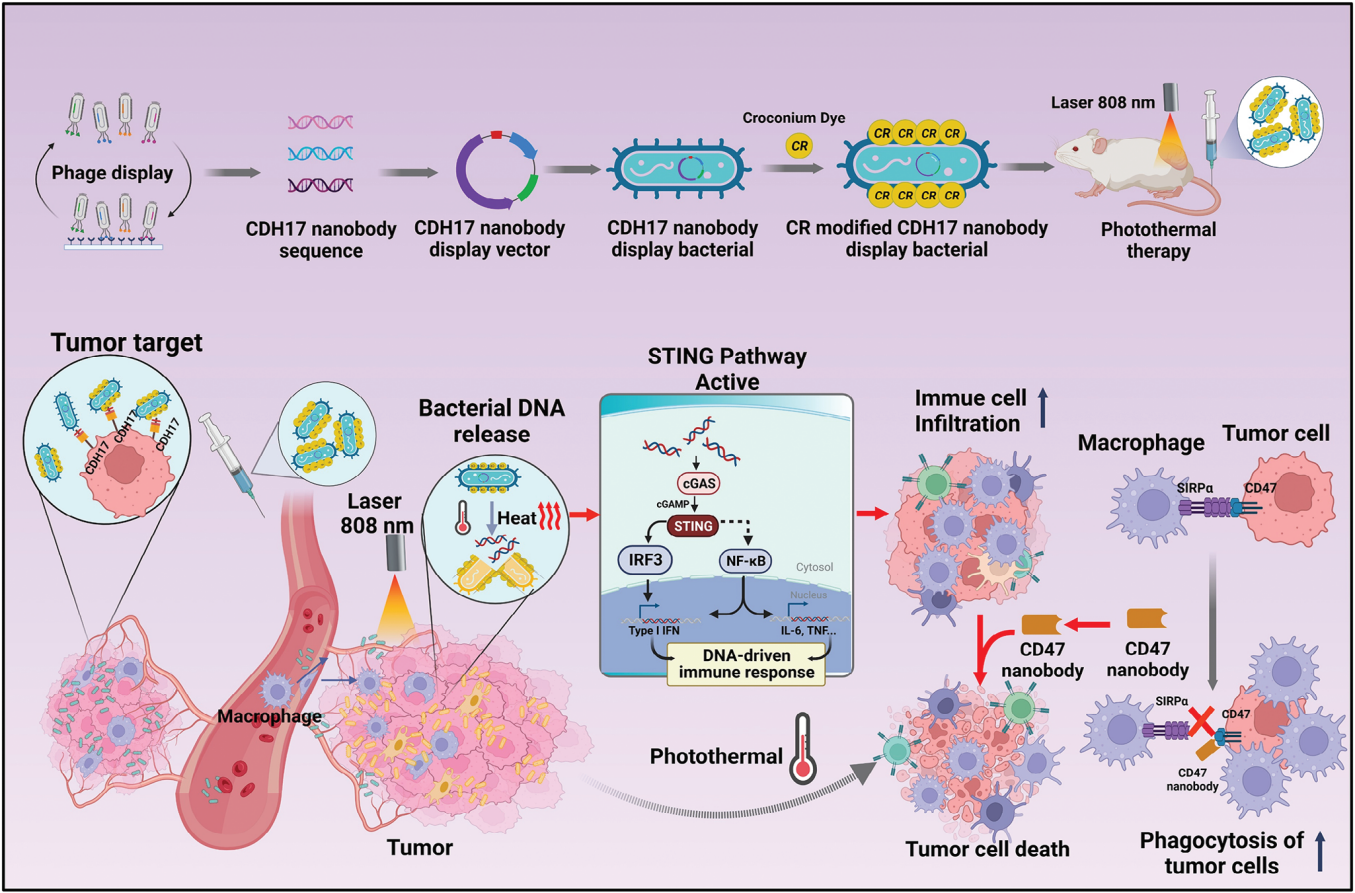
Schematic illustration of engineered biohybrid bacteria MG1655 targeting CDH17‐positive tumors to suppress tumor growth through photothermal effect and STING activation in macrophages. The CDH17 nanobodies applied in biohybrid bacteria were screened using phage display and deep sequencing technology. Bacteria were engineered to display nanobodies on the surface and further conjugated with CR dye. The resultant biohybrid bacteria can specifically home to tumor mass (gastric, colorectal, and pancreatic tumors) overexpressing CDH17 protein. Laser irradiation at 808 nm can directly kill tumor cells through the photothermal effect and simultaneously activate the STING pathway in tumor‐associated macrophages via the release of internal nucleic acid from bacteria and cancer cells. Therefore, macrophage infiltration is further induced, and the addition of CD47 nanobody based on biohybrid bacteria plus irradiation eliminates tumors.

In this study, we developed a biohybrid bacterial system through genetic engineering and biomaterial hybridization, which exhibited efficient tumor targeting and activation of anti‐tumor immunity (**Scheme** **1**). Furthermore, to enhance the tumor‐targeting ability of bacteria, we isolated nanobodies that specifically recognize cadherin 17 (CDH17) protein, which is highly expressed on the membranes of gastrointestinal (GI) cancer cells in gastric, colorectal, and pancreatic cancers. These nanobodies were then engineered onto the surface of bacteria. Additionally, to leverage the delivery capacity of bacteria and synergistically reprogram the TME, we conjugated the photothermal material croconium (CR) dye on bacteria. The resultant biohybrid bacterial system demonstrated outstanding tumor‐targeting capability and photothermal therapeutic function. In vivo experiments revealed that this engineered bacterial system exhibited excellent tumor targeting and penetration effects in CDH17‐positive solid tumors, including desmoplastic stroma‐enriched pancreatic cancer. The photothermal effect mediated by the CR dye from bacteria not only induced cancer cell death but also triggered the release of internal nucleic acids from both the bacteria and tumor cells, which in turn activate the STING pathway in macrophages within the TME, which resulted in increased infiltration of macrophages, rather than T cells, into the TME, and create a cascading effect. The biohybrid bacteria‐mediated photothermal therapy (PTT) effectively inhibited tumor growth and significantly prolonged the survival of tumor‐bearing mice. Furthermore, when combined with checkpoint blockade using CD47 nanobody, the biohybrid bacteria successfully eradicated the tumors and considerably extended survival of the mice.

## 2 Results

### 2.1 CDH17 Nanobody Isolation and Characterization

**Figure 1:**
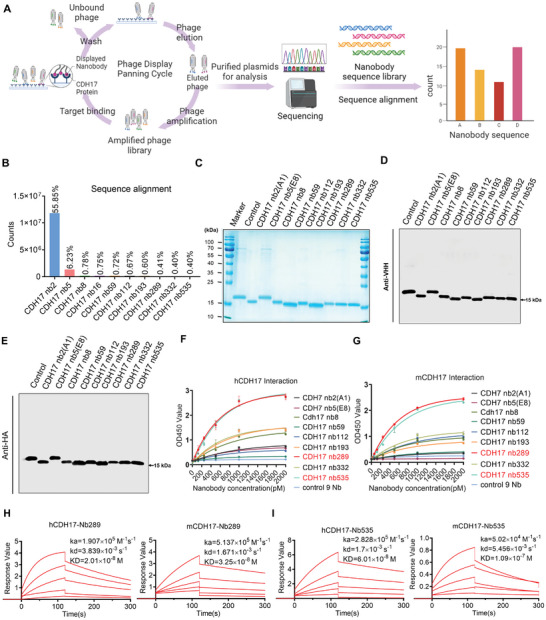
Phage display combined with deep sequencing technology to screen potential high‐affinity CDH17 nanobodies. A) The flow chart of phage display biopanning combined with deep sequencing analysis to screen CDH17 nanobodies. B) Ten CDH17 nanobody candidate sequences obtained by deep sequencing; the vertical axis represents the sequence counts, and the percentage of nanobody sequence frequencies is marked on the bar graph. C) SDS‐PAGE analysis of nanobodies after purification. D) Western blot of nanobodies detected with anti‐VHH antibody; the size of the nanobodies is ≈15 kDa. E) Western blot of nanobodies detected with anti‐HA tag antibody. HA tag was integrated at the C‐terminal of nanobody sequences. F) Analysis of the binding activity of candidate nanobodies to human CDH17 protein using ELISA (n = 4). G) Analysis of the binding activity of candidate nanobodies to murine CDH17 protein with ELISA (n = 4). H) Affinity determination of Nb289 against human and murine CDH17 proteins using surface plasmon resonance (SPR) assay. I) Affinity determination of Nb535 against human and murine CDH17 proteins using SPR assay.

**Figure 2:**
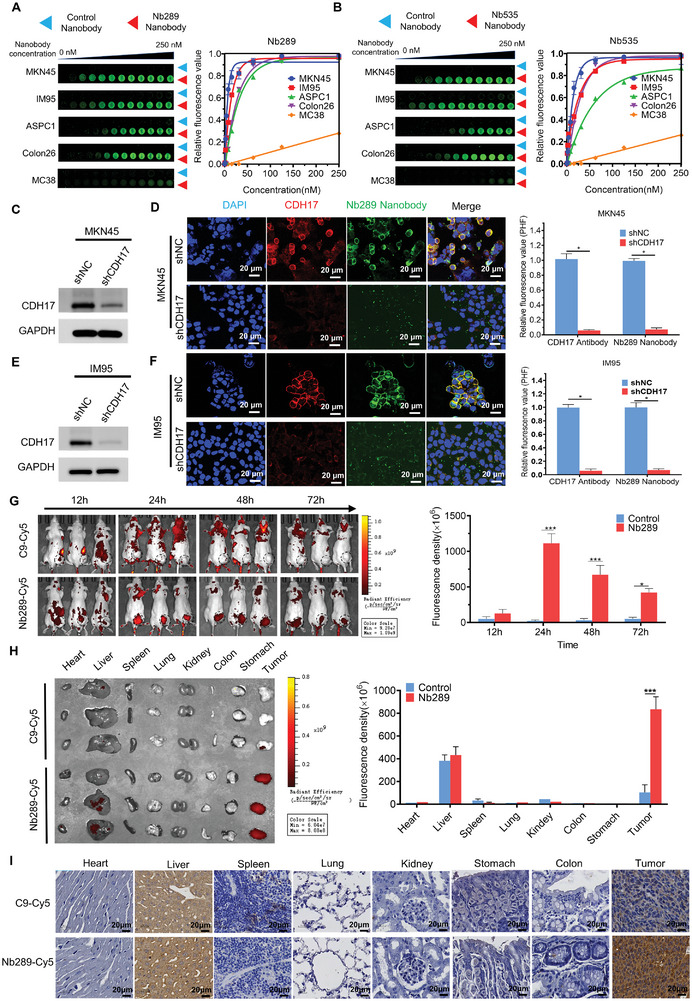
Nb289 nanobody can target gastric, colorectal, and pancreatic cancers with CDH17 expression. A) Cell ELISA to detect the binding of Nb289 to various cancer cells. Nb289 and control nanobody C9 were serially diluted from 250 nM and incubated with CDH17 positive and negative cells (n = 3). Data are representative of three duplicates. B) Cell ELISA to detect the binding of Nb535 to each cell as described in A (n = 3). C) Western blot determination of CDH17 knockdown in MKN45 cells. D) Immunofluorescence staining (left) and quantification (right) for the specificity determination of Nb289 against CDH17 in MKN45 cells with/without CDH17 knockdown. Scale bars, 20 µm. E) Western blot determination of CDH17 knockdown in IM95 cells. F) Immunofluorescence staining (left) and quantification (right) for the specificity determination of Nb289 against CDH17 in IM95 cells with/without CDH17 knockdown. Scale bars, 20 µm. G) In vivo imaging analysis of Nb289 in MKN45 tumor model. C9 and Nb289 labeled with Cy5 fluorescent molecules, imaging was performed at different time points (12, 24, 48, and 72 h) after nanobody injection (1 mg kg^−1^ body weight, n = 3 per group). H) *Ex vivo* imaging analysis of the major organs of mice that received nanobody injection. The organs were collected from mice for in vivo imaging analysis after 72 h circulation (n = 3 per group). I) Immunohistochemical detection of nanobody distribution in major organs from H by anti‐VHH antibody (n = 3 per group). Scale bars, 20 µm. *P < 0.05, **P < 0.01, and ***P < 0.001.

Validating new biomarkers for tumor diagnosis and therapy is crucial, as current targets such as EGFR, HER2, and VEGFR2 are insufficient for treating various solid tumors and dealing with emerging drug resistance. CDH17 has been extensively explored as a diagnostic and potential therapeutic marker in digestive system cancers, including gastric, colorectal, and pancreatic cancers.^[^ ^11^ ^]^ Various modalities targeting CDH17 have been applied for imaging diagnosis and therapy in CDH17‐positive cancers, confirming CDH17 as a reliable, valuable, and promising biomarker for potential clinical translation.^[^ ^11^, ^12^ ^]^ To further confirm the value of CDH17 as a targeted protein, the expression of CDH17 protein were recapitulated by immunohistochemical staining on tissue microarrays including gastric, colorectal, and pancreatic cancers. Our results aligned with previous studies, revealing high CDH17 expression in these three cancer types, with expression levels positively associated with malignancy (Figures S1–S3; and Tables S1–S3, Supporting Information). The positive rates for CDH17 were 77.4%, 75.9%, and 63.8% in gastric, colorectal, and pancreatic cancers, respectively, indicating CDH17 as a potential target for cancer therapy. Flow cytometry and immunofluorescent staining of several gastric (IM95, MKN45, TMK1, and AGS), colorectal (HCT116 and Colon26), and pancreatic (ASPC1 and PancO2) cancer cell lines confirmed that CDH17 was predominantly expressed on the cell membrane surface (Figures S4 and S5, Supporting Information). In a previous study, we identified two nanobodies (E8 and A1) that targeted human CDH17 using phage display biopanning and phage enzyme‐linked immunosorbent assay (ELISA). We demonstrated that the E8 nanobody exhibited excellent targeting ability for gastric cancer imaging and displayed anti‐tumor activity in vivo when fused with the truncated toxin PE38.^[^ ^11c^ ^]^ However, these nanobodies were unable to bind to murine CDH17 protein, making them unsuitable for testing related drugs in immunocompetent mice inoculated with murine CDH17‐positive tumor cells, such as Colon26, CT26, or PancO2. As CDH17 protein is highly conserved, with 79.3% sequence homology of between human and murine CDH17 proteins (Figure S6, Supporting Information), we speculated that the sub‐library collected from the last round of nanobody screening against human CDH17 should contain clones that recognize both human and murine CDH17 proteins. However, routine phage nanobody ELISA assay could only identify highly‐enriched clones from a limited number of phage plaques picked from the library (≈200 clones). Deep sequencing enables the analysis of millions of sequences from the library in a short time, and these could be ranked according to their frequencies using bioinformatic analysis to identify rare clones with high affinity. After deep sequencing analysis of the CDH17 nanobody phage library from our previous study (**Figure** **1**A),^[^ ^11c^ ^]^ the top 10 nanobodies based on their frequencies were selected for further assessment (Figure 1B), with the top two nanobodies corresponding to A1 and E8. This is consistent with the phage ELISA results from our previous study.^[^ ^11c^ ^]^ To further characterize these nanobodies, all 10 nanobodies and a control nanobody (C9) were cloned into a prokaryotic vector (pColdII, TAKARA), and the nanobody proteins were purified. Most of the nanobodies were obtained with high purity, except for Nb16, due to its poor solubility (Figure 1C). The nanobodies were confirmed using an anti‐variable domain of heavy chain‐only (VHH) antibody, which recognizes the frame sequences of nanobodies (Figure 1D), or an anti‐HA‐tag antibody, which was integrated at the C‐terminal of nanobodies (Figure 1E). ELISA results revealed that nanobodies Nb289 and Nb535 exhibited the highest affinity for human and murine CDH17 proteins among all the nanobodies, whereas the top two nanobodies, A1 and E8, showed much lower binding activity to human CDH17 compared to Nb289 and Nb535 and almost no binding to murine CDH17 (**Figure** **2**F,G). This result indicates that deep sequencing analysis can be leveraged to search for rare nanobody clones missed by traditional phage ELISA methods.

Considering the excellent performance of Nb289 and Nb535 against human and murine CDH17 proteins in ELISA, further validation of their antigen‐binding specificity was conducted using GFP protein as a negative control. The results demonstrated that both nanobodies did not react with GFP (Figure S7, Supporting Information). The affinity constants (KD values) of Nb289 against human and murine CDH17 proteins, as determined using surface plasmon resonance (SPR) analysis, were 20.1 and 32.5 nM, respectively (Figure 1H). For Nb535, the affinity constants were 60.1 and 109 nM for human and murine CDH17 proteins, respectively (Figure 1I). Interestingly, sequence alignment revealed only one amino acid difference between Nb289 and A1, with a replacement of threonine by lysine at amino acid position 44 of A1 (Figure S8, Supporting Information). Further studies are needed to understand why Nb289 binds to murine CDH17 but not A1. Overall, these findings suggest that the nanobodies Nb289 and Nb535, identified through deep sequencing, hold potential for use in tumor models expressing both human and murine CDH17 antigens, thereby extending the applications of CDH17 nanobodies to a certain extent.

### 2.2 Nb289 Nanobody can Target CDH17‐Positive Cancers In Vitro and In Vivo

Due to the superior binding activity of Nb289 and Nb535 among all the nanobodies to human and murine CDH17 proteins, they were further investigated for in vitro cancer cell studies. Cell ELISA data indicated that both Nb289 and Nb535 nanobodies effectively recognized MKN45, IM95, ASPC1, and Colon26 cells, all of which expressed CDH17 protein (Figures S5 and S6, Supporting Information). In contrast, the control nanobody C9 was undetectable in these cells, and the CDH17‐negative cancer cell line MC38 showed no signal for all three nanobodies. This suggests that Nb289 and Nb535 nanobodies have strong specificity for CDH17 (Figure 2A,B). Given relatively better binding affinity and in vitro cell ELISA performance of Nb289 for human and murine CDH17, Nb289 was selected for subsequent studies. CDH17 knockdown stable cell lines were established in MKN45 and IM95 gastric cancer cells to confirm the specificity of Nb289 nanobody to CDH17 (Figure 2C,E). Immunofluorescence staining results revealed that the knockdown of CDH17 significantly attenuated the binding activity of Nb289 nanobody compared to wild‐type cells (Figure 2D,F), indicating that Nb289 nanobody specifically interacts with CDH17 on the cell membranes. To further validate the in vivo targeting ability of Nb289, we labeled the nanobody with the fluorescent dye Cy5 (Figure S9, Supporting Information) and assessed its ability to image CDH17‐positive tumors in vivo. A gastric tumor model induced by MKN45 cells was exploited for in vivo imaging evaluation at different time points (Figure 2G; Figure S9, Supporting Information). A lower dose of Cy5‐labeled Nb289 nanobody administered at 1 mg kg^−1^ showed gradual accumulation at tumor sites 12 h after intravenous injection, reaching its peak at 24 h. The signal at tumor sites then faded away from 48–72 h, whereas the control nanobody did not exhibit noticeable enrichment signals in the tumor sites throughout the entire period (72 h). Ex vivo tissue imaging after 72 h further confirmed that Nb289 nanobody specifically accumulated in tumor tissues, with a minimal signal detected in most control organs such as the heart, spleen, lung, kidney, stomach, and colon, except for the liver. The liver, as the main metabolic organ, may be responsible for nanobody metabolism or phagocytosis through the mononuclear phagocyte system. Tissue staining of various organs also revealed detectable levels of Nb289 nanobody in tumor tissues, with no apparent signal found in the aforementioned control organs except for the liver (Figure 2J). In contrast, the control nanobody was only detectable in the liver tissues due to phagocytosis, with minimal staining observed in tumor tissues, possibly attributed to the enhanced permeability and retention (EPR) effect but not in other organs (Figure 2J). Previous studies have reported high expression of CDH17 in normal colon tissues, suggesting that it may not be a suitable target for GI tumors.^[^ ^11^, ^13^ ^]^ However, in our study, we did not observe enrichment of Nb289 nanobody in colon tissues, despite Nb289 exhibiting good affinity to murine CDH17. To investigate this further, we performed CDH17 immunofluorescence staining on normal colorectal tissues and unveiled that CDH17 protein was primarily distributed in the tight junctions between colorectal epithelial cells, with a lower expression on the luminal surface and basal layer of the intestine. This implies that nanobodies or other drugs may be difficult to reach CDH17 expressed on the cell membranes of normal intestine tissues (Figure S11, Supporting Information). These findings align with the previous report.^[^ ^14^ ^]^

In addition, conjugated nanobodies with image contrasts such as various isotopes and fluorescent dyes facilitate to conduct the same‐day tumor imaging and detection due to their rapid clearance and high permeability to tumors, which is difficult for full‐length monoclonal antibodies.^[^ ^15^ ^]^ Thus, we further investigated the imaging performance for Cy5‐labeled Nb289 in MKN45 tumors with a higher dose (5 mg kg^−1^). The data indeed suggested that Cy5‐labeled Nb289 nanobody peaked at the tumor site within 2 h and then gradually faded out (Figure S11A,B, Supporting Information); compared with control nanobody, Cy5‐labeled Nb289 nanobody produced stronger fluorescent signals in tumors during the detection period (0.5–10 h). Ex vivo organ image data showed that both of Cy5‐labeled nanobodies (Nb289 and control Nb) could be observed in liver and kidney tissues besides tumors in which strongest signals could be identified for Nb289 (Figure S11C,D, Supporting Information).

Taken together, these results demonstrate that the nanobody Nb289 can be employed for imaging detection of CDH17‐positive cancers and has the potential for delivering various payloads for therapy against CDH17‐expressing tumors.

### 2.3 Construction and Characterization of Nb289 Nanobody‐Engineered Bacteria

**Figure 3:**
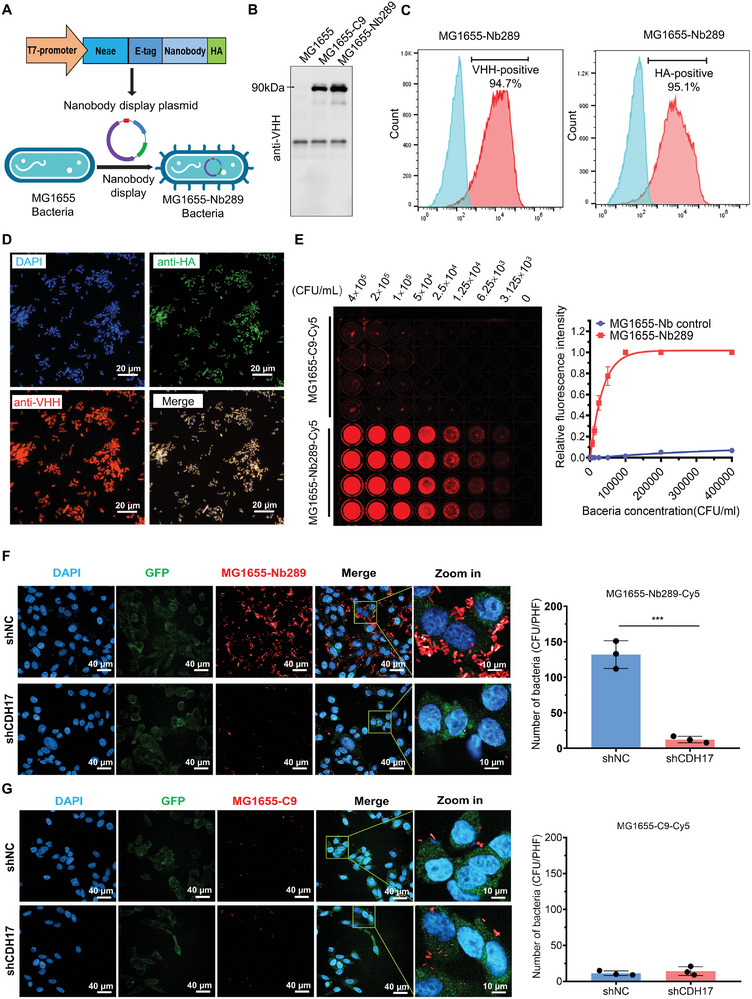
Construction of nanobody‐engineered bacteria and assessment in vitro. A) Schematic diagram of nanobody‐displayed bacteria. B) Western blot detection of the nanobody expression in the engineered bacteria MG1655. The nanobody fused to the C‐terminal fragment of EHEC intimin protein was ≈90 kDa, and the lower band was a non‐specific signal. C) Bacterial flow cytometry assay to determine nanobody display efficiency on the bacterial outer membrane surface with anti‐VHH and HA tag antibodies. Unmodified MG1655 bacteria were used as a negative control. D) Immunofluorescence staining for detection of the nanobody displayed on the bacterial outer membrane. The antibodies targeting nanobody frames and HA tags were used for nanobody identification. Scale bars, 20 µm. E) ELISA assay with nanobody‐engineered bacteria MG1655 to examine the binding activity to human CDH17 protein. Engineered bacteria were labeled with Cy5 dye and co‐incubated with purified human CDH17 with serial diluted MG1655 (n = 4 per concentration). F) Cell immunostaining and quantification of MKN45 cells with or without CDH17 knockdown through incubation with Nb289 nanobody‐engineered bacteria labeled by Cy5. The green fluorescence was derived from the lentiviral vector expressed GFP, and the red fluorescence was produced from the engineered bacteria labeled with Cy5. Scale bars left 40 µm, right 10 µm. G) Cell immunostaining (left) and quantification (right) of MKN45 cells with or without CDH17 knockdown through incubation with control nanobody‐engineered bacteria labeled by Cy5. Scale bars, 40 µm (left), 10 µm (right). * *P* < 0.05, **P < 0.01, and ***P < 0.001.

To establish a bacteria delivery strategy targeting tumor tissues with CDH17 nanobody, we engineered the nanobody Nb289 onto the surface of non‐pathogenic *E. coli* MG1655. This particular strain of *E. coli* has been extensively utilized to develop a variety of modalities for cancer therapy and detection.^[^ ^9^, ^16^ ^]^ The gene sequences encoding the nanobody were inserted into the pNeae2‐HA plasmid, as previously described, to fuse nanobody with the C‐terminal fragment of EHEC intimin (Neae; residues 1–654) on the bacterial outer membrane (**Figure** **3**A; Figure S12, Supporting Information).^[^ ^17^ ^]^

**Figure 4:**
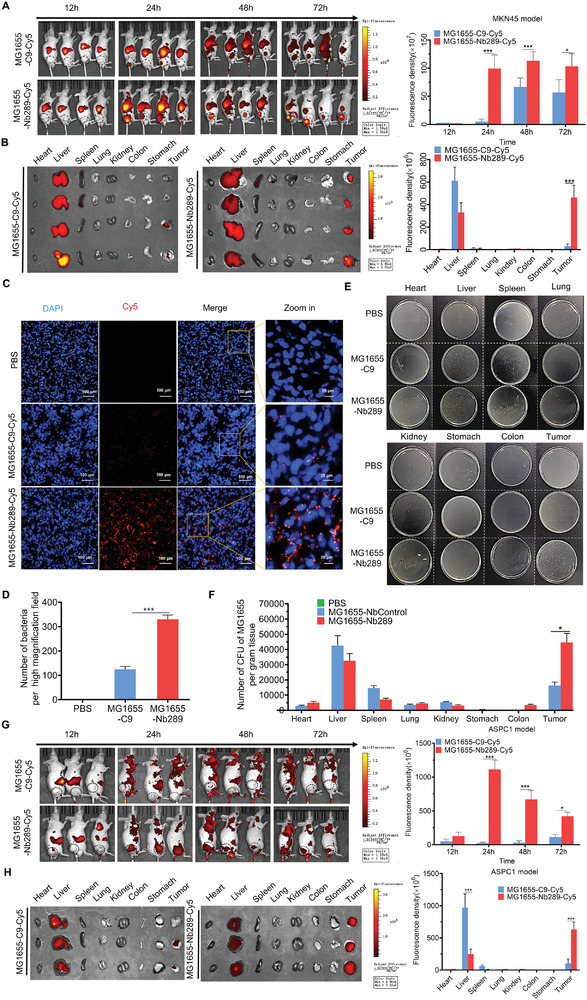
Nb289 nanobody enhances the homing ability of bacteria to tumors with high CDH17 expression in vivo. A) In vivo imaging analysis of Nb289‐engineered bacteria in MKN45 gastric cancer model. C9‐ and Nb289‐engineered bacteria were labeled with Cy5 dye; imaging was performed at different time points (12, 24, 48, and 72 h) after bacterial injection (5 × 10^6^ CFU per mouse, n = 4 per group). B) Ex vivo organ imaging analysis after administration of C9‐ and Nb289‐engineered bacteria. Organs were collected from in vivo imaged mice 72 h after circulation (n = 4 per group). C) Immunofluorescence staining analysis of bacteria enriched in tumors. Tumors were dissected from in vivo imaged mice bearing MKN45 gastric cancer (n = 4 per group). Scale bars, 100 µm (left), 20 µm (right). D) Quantification analysis of bacterial staining in tumor tissues; nine high magnification fields of each sample were analyzed. E) Bacterial colony analysis in various tissues after treatment with engineered bacteria. The tissues were collected from mice receiving bacteria administration for 72 (n = 3 per group). F) Quantification of bacterial colony number in various tissues from E (n = 3 per group); three duplicates were conducted for each sample to calculate the average colony number. G) In vivo imaging analysis of Nb289‐engineered bacteria in ASPC1 pancreatic cancer model. C9‐ and Nb289‐engineered bacteria were labeled with Cy5 dye; imaging was performed at different time points (12, 24, 48, and 72 h) after bacterial injection (5 × 10^6^ CFU per mouse, n = 3 per group). H) Ex vivo imaging analysis of the major organs 72 h after treatment with C9‐ and Nb289‐engineered bacteria. The organs were dissected from in vivo imaged mice from G (n = 3 per group), *P < 0.05, **P < 0.01, and ***P < 0.001.

Fusion proteins were confirmed with anti‐VHH antibody by western blotting, which showed an ≈90 kDa protein for Nb289 and C9 control (Figure 3B). Flow cytometry data also uncovered that both nanobodies (Nb289 and C9 control) could be displayed on the surface of the bacterial outer membrane in more than 90% of the bacteria under suitable induction conditions (Figure 3C; Figure S13, Supporting Information). Surface staining on engineered bacteria yielded results similar to those of flow cytometry analysis, suggesting that almost all bacteria could be identified with HA and VHH antibodies (Figure 3D). To confirm the multivalent nanobody display in bacteria, we further designed the experiment and determined that ≈23 000 nanobodies were displayed onto the surface of each engineered bacterium and similar numbers were observed in both of MG1655‐Nb289 and MG1655‐C9 (Figure S14, Supporting Information). It has been found that bacteria expressing over 5000 nanobodies show excellent targeting effect^[^ ^18^ ^]^ implying that our engineered bacterial system holds great potential for targeting CDH17‐positive tumors. Direct ELISA assays were performed to verify whether the engineered bacteria could also recognize the purified CDH17 protein, validating that fluorescent dye Cy5‐labeled Nb289‐engineered MG1655 could significantly bind to CDH17 antigen in a concentration‐dependent manner when compared with the control C9‐engineered MG1655 (Figure 3E). Similarly, cell staining data with engineered bacteria also disclosed that Nb289‐engineered MG1655 could specifically recognize CDH17 expressed on MKN45 cancer cells since CDH17 knockdown completely abolished the binding activity of Nb289‐engineered bacteria; C9 bacteria showed negative binding activity to MKN45 cells unrelated to CDH17 expression (Figure 4f,g). Collectively, we successfully constructed nanobody‐engineered bacteria and demonstrated that Nb289‐engineered MG1655 could specifically interact with CDH17 protein in vitro, implying the enormous potential of Nb289‐engineered MG1655 for in vivo applications.

### 2.4 Nanobody Nb289 Enables More Bacteria to Efficiently Home to Tumors with High CDH17 Expression In Vivo

Given the strong in vitro binding activity of Nb289‐engineered MG1655 to CDH17, we further examined whether the engineered bacteria could effectively target tumor mass in a tumor‐bearing mouse model after systemic administration. Using Cy5‐labeled engineered bacteria, we observed that Nb289‐engineered MG1655 efficiently homed to MKN45 gastric tumor tissue within 24 h after tail vein injection, exhibiting faster accumulation compared to C9‐engineered bacteria. The signals in tumors treated with Nb289‐engineered bacteria remained consistently high for 24–72 h after injection. In contrast, the accumulation of control C9‐engineered bacteria in tumors was much lower during the 72 h period, even though the bacteria reached a relatively higher level at 48 h after circulation (**Figure** **4**A). Ex vivo organ imaging after 72 h of circulation further revealed that Nb289‐engineered bacteria were primarily found in the liver and tumor tissues. Importantly, the accumulation of Nb289‐engineered bacteria in liver tissues was lower than that of the C9 group although it was unavoidable for the most of drugs including biomaterials and bacteria to accumulate into liver tissues, suggesting that nanobody‐mediated targeting modification effectively inhibited the enrichment of bacteria in non‐tumor tissues (Figure 4B). Tumor tissue immunofluorescence staining further confirmed a significantly higher presence of Nb289 engineered bacteria in tumors compared to C9 engineered bacteria 72 h after bacteria injection (Figure 4C,D). To support this conclusion, various organs collected from bacteria‐injected mice were homogenized and cultured on plates for colony growth. The colony number recovered from tumor tissues in the Nb289‐engineered bacteria‐treated group was much higher than that from most control organs, such as the heart, spleen, lung, kidney, stomach, and colon, as well as higher than tumors treated with C9‐engineered bacteria (Figure 4E,F). Consistent with ex vivo organ imaging, the colony number in liver tissues from the Nb289 group was slightly lower than that in the C9‐treated liver tissues (Figure 4E,F). Notably, no significant enrichment of Nb289‐engineered bacteria was observed in colon tissues, which have high CDH17 expression, consistent with the tissue distribution results for Cy5‐labeled Nb289 nanobody. Immunohistochemical staining using an anti‐VHH antibody confirmed the presence of bacteria modified with nanobodies in liver and tumor tissues in both groups, with much stronger signals observed in tumors receiving Nb289‐engineered bacteria than C9‐treated tumors (Figure S15, Supporting Information). Slight staining of bacteria was also observed in the spleen tissues of both groups, likely due to phagocytosis by macrophages. Minimal bacteria were detectable in the other control organs for both groups (Figure S15, Supporting Information). Since CDH17 in cancer cell membrane could mediate the internalization of the corresponding nanobodies and nanobody‐modified extracellular vesicles,^[^ ^11^, ^19^ ^]^ we also demonstrated here that Nb289‐engineered bacteria MG1655 could be efficiently internalized by CDH17‐positive cancer cells in vitro and in vivo (Figure S16A,B, Supporting Information). These results clearly demonstrate that MG1655 bacteria modified with targeted nanobodies had significantly enhanced selectivity for tumor tissues and improved bacterial homing activity. CDH17 is also highly expressed in pancreatic cancer (Figure S1, Supporting Information), and pancreatic cancer is characterized by atypical hyperplastic glands in a stroma‐enriched microenvironment (connective tissue hyperplasia), which is hypovascularized and highly hypoxic.^[^ ^20^ ^]^ This unique property of pancreatic cancer renders it difficult for drug penetration.^[^ ^21^ ^]^ Therefore, we further examined whether Nb289‐engineered bacteria could efficiently home to pancreatic cancer. Interestingly, we observed that C9‐engineered bacteria had difficulty reaching pancreatic tumor tissues even after 72 h of circulation in the ASPC1‐induced pancreatic cancer model. In contrast, Nb289‐engineered bacteria exhibited excellent tumor infiltration ability in pancreatic tumor tissues, with peak accumulation occurring as early as 24 h after injection and gradually decreasing thereafter (Figure 4G,H). Furthermore, ex vivo fluorescence imaging of dissected organs revealed that Nb289‐engineered bacteria were more enriched in tumor tissues than liver tissues, whereas C9‐engineered bacteria mainly accumulated in liver tissues (Figure 4H). These results indicate that unmodified bacteria cannot infiltrate pancreatic cancer with stiff tissue structure by their own intrinsic tropism, but targeted nanobody‐engineered bacteria remarkably improve the homing ability toward pancreatic cancer. Collectively, the results from both animal models demonstrate that the modification of Nb289 nanobodies onto the outer membrane of MG1655 bacteria significantly enhances the targeting ability in vivo, reflected not only in the speed of accumulation in tumor tissues but also in the quantity of enrichment. More importantly, Nb289 engineered bacteria MG1655 have significantly improved penetration into desmoplastic pancreatic cancer. Therefore, Nb289‐engineered MG1655 holds great promise for the treatment of CDH17‐overexpressing tumors, including pancreatic cancer.

### 2.5 Photothermal Agent Coupling in Engineered Bacteria and Characterization

**Figure 5:**
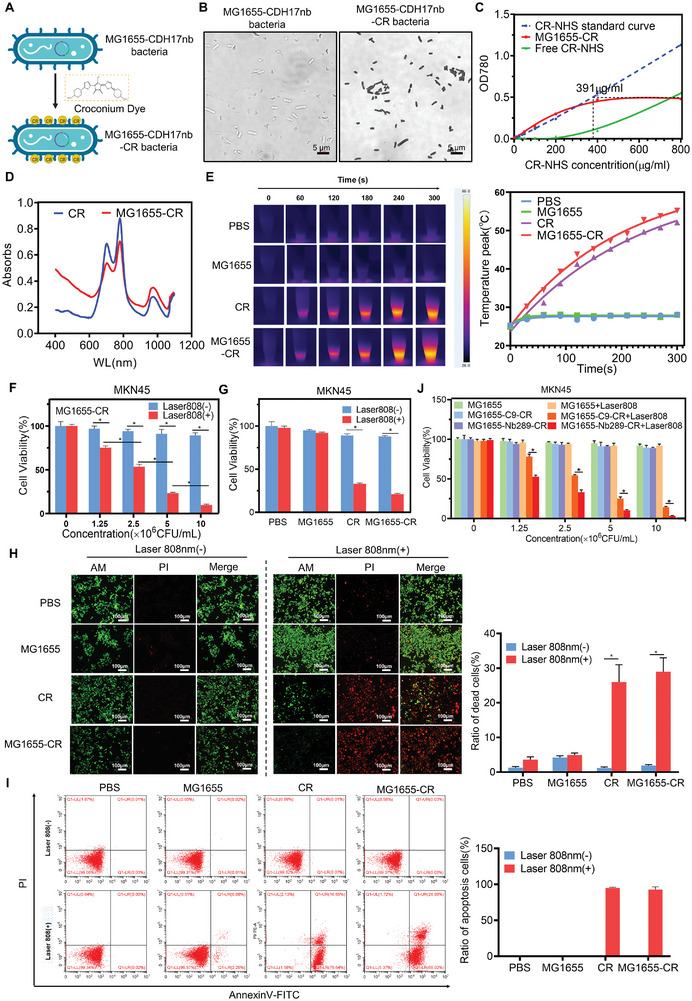
Construction and characterization of biohybrid bacteria through CR coupling with engineered bacteria. A) Schematic illustration of CR photothermal molecules conjugated with engineered bacteria MG1655. B) Images of MG1655‐CR visualized using an optical microscope. Scale bars, 5 µm. C) Concentration determination of CR molecules modified onto bacteria; 10^9^ CFU of bacteria were used in 1 ml PBS for the conjugation in different concentrations of CR‐NHS. Quantity of CR‐NHS was determined based on the absorption of the solution at 780 nm. D) UV–vis absorption spectra of CR and MG1655‐CR. E) Thermal images and temperature quantification of PBS, MG1655, CR, and MG1655‐CR under irradiation with 0.8 W/cm^2^ 808 nm laser for 5 min. F) Cell viability detection of MKN45 gastric cancer cells under treatment with different concentrations of MG1655‐CR with or without 808 nm laser irradiation for 5 min via CCK‐8 assay (n = 4). G) Cell viability examination of MKN45 gastric cancer cells under treatment with PBS, MG1655, CR, and MG1655‐CR with or without 808 nm laser irradiation for 5 min via CCK‐8 assay (n = 4). H) Live and dead cell staining for MKN45 gastric cancer cells after treatment with PBS, MG1655, CR, and MG1655‐CR with or without 808 nm laser irradiation for 5 min via calcein AM/PI staining (n = 3). Scale bars, 100 µm. I) Apoptosis detection of MKN45 gastric cancer cells following treatment with PBS, MG1655, CR, and MG1655‐CR with or without 808 nm laser irradiation for 5 min via Annexin V/PI staining and flow cytometry (n = 3). *P < 0.05. J) Cell viability detection of MKN45 gastric cancer cells following treatment with different concentrations of MG1655, MG1655‐C9‐CR, and MG1655‐Nb289‐CR with 808 nm laser irradiation for 5 min via CCK‐8 assay (n = 4). Bacteria unbound to cells were washed away before laser irradiation in the experiment.

PTT, a localized therapeutic modality that converts light into thermal energy, can effectively ablate tumors through direct thermal killing and modulating the TME.^[^ ^22^ ^]^ PTT directly kills tumor cells by local heating and promotes immune cell infiltration by triggering local inflammation, which favors anti‐tumor immunity.^[^ ^22^, ^23^ ^]^ However, efficient systemic delivery of photothermal sensitizers into tumor tissues remains a major challenge for PTT. In this study, to fully utilize the potent payload delivery capacity of engineered bacteria, we synthesized a CR photothermal agent with excellent photothermal efficiency and high photostability^[^ ^24^ ^]^ (Figure S17, Supporting Information) and conjugated it onto the surface of the engineered bacteria through amino coupling, as previously reported, to produce biohybrid bacteria modified with both nanobody and CR (**Figure** **5**A).^[^ ^25^ ^]^ The bacteria displayed a gray‐black color after CR modification (Figure 5B; Figure S18B, Supporting Information). As the maximum absorption peak of the CR molecule is at 783 nm, its concentration in solution can be quantified using a 780 nm spectrophotometer. We plotted the standard curve of CR at 780 nm absorbance, and the saturation concentration of CR‐modified bacteria was 391 µg 10^−9^ CFU bacteria (Figure 5C). The fluorescence absorption peak of the CR‐modified biohybrid bacteria was almost identical to that of free CR molecules (Figure 5D), and the 808 nm laser effectively irradiated the biohybrid bacteria or free CR to reach a temperature elevation of 50 °C or above within 5 min. MG1655‐CR performed slightly better than free CR in terms of temperature elevation upon irradiation, possibly due to the aggregation of CR on the bacterial surface. However, the irradiation did not affect PBS or unmodified natural bacteria MG1655 (Figure 5E). To further investigate the in vitro photothermal killing effect of the biohybrid bacteria, we conducted a photothermal treatment assay with MKN45 cells. The CCK‐8 assay showed that the CR‐modified natural MG1655 bacteria exhibited a significant concentration‐dependent killing effect on MKN45 gastric cancer cells under 0.5 W cm^−2^ of 808 nm laser irradiation for 5 min (Figure 5F). The photothermal killing effect of CR‐modified MG1655 bacteria was slightly stronger under these conditions compared to the same concentration of free CR molecules, consistent with the temperature elevation data after irradiation (Figure 5G). To confirm whether CR dye modified in bacteria could induce cell death and apoptosis, live and dead cell staining with PI (Propidium iodide) and calcein AM was performed. The staining results showed that both free CR and MG1655‐CR resulted in noticeable cancer cell death, with strong PI signals and weak AM staining upon irradiation (Figure 5H). Apoptosis analysis with Annexin V/PI also revealed that both free CR and MG1655‐CR induced early (Annexin V^+^/PI^−^) and late (Annexin V^+^/PI^+^) apoptosis upon irradiation with the 808 nm laser (Figure 5I). Furthermore, we compared the thermal killing effect between CR‐modified bacteria displaying Nb289 nanobody (Nb289‐MG1655‐CR) and the C9 control nanobody (C9‐MG1655‐CR) in CDH17‐overexpressing MKN45 cancer cells. The biohybrid bacteria Nb289‐MG1655‐CR exhibited a stronger killing effect on CDH17‐overexpressing MKN45 cancer cells than C9‐MG1655‐CR, indicating that CDH17 molecules on cancer cells captured more biohybrid bacteria Nb289‐MG1655‐CR (Figure 5J). Finally, we further investigated whether CR modification on bacteria affects the homing ability of engineered MG1655. We analyzed the colony number of bacteria enriched in tumor tissues after injection of MG1655‐Nb289 and MG1655‐Nb289‐CR (Figure S19A, Supporting Information). The results showed that the modification of CR dye did not affect targeting ability of engineered bacteria in tumors (Figure S19B, Supporting Information); both of bacteria produced similar colony numbers in tumors with a much higher enrichment than MG1655‐C9 control.

These results collectively demonstrate that the CR photothermal sensitizer can be efficiently coupled to bacteria and induce cancer cell apoptosis upon irradiation. Moreover, the targeted biohybrid bacteria Nb289‐MG1655‐CR can exert a more pronounced thermal killing effect on CDH17‐overexpressing cancer cells due to the selectivity of Nb289 to CDH17 molecules.

### 2.6 Nb289‐MG1655‐CR Mediated PTT can Effectively Inhibit Tumor Growth In Vivo

**Figure 6:**
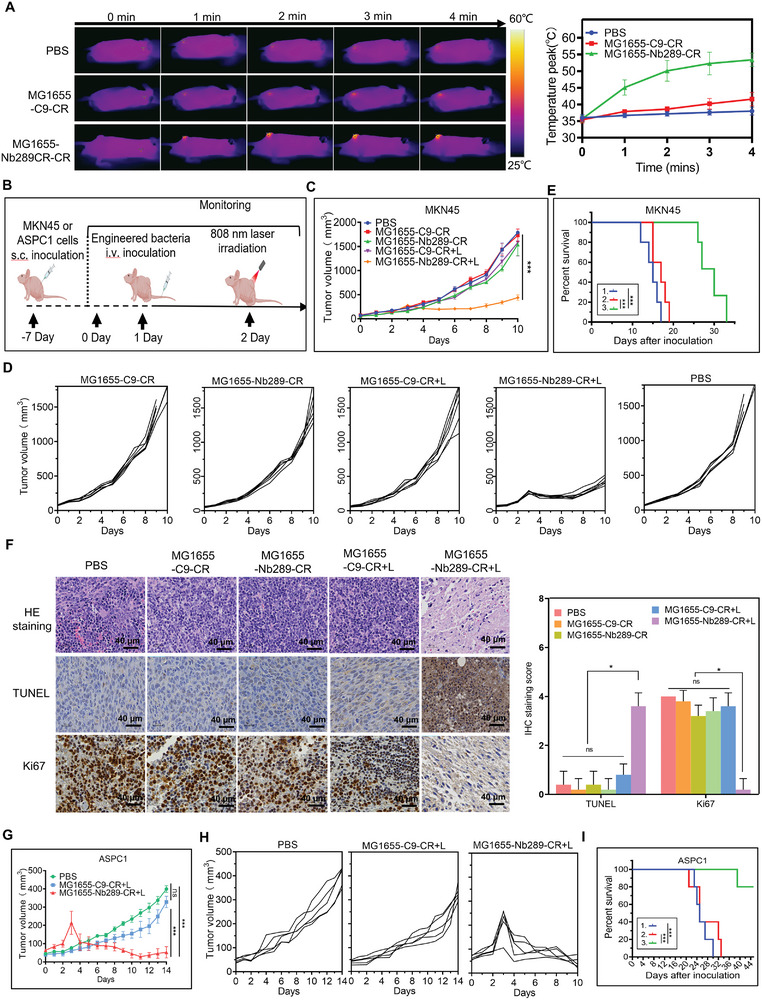
Nb289‐MG1655‐CR biohybrid bacteria‐mediated photothermal therapy effectively inhibits tumor growth in vivo. A) The temperature changes and NIR thermal images of tumors during laser irradiation after intravenous injection of PBS, MG1655‐C9‐CR, and MG1655‐Nb289‐CR (5 × 10^6^ CFU per mouse, n = 3 per group). B) Treatment schedule for MKN45 gastric and ASPC1 pancreatic subcutaneous tumors. C) Tumor growth curves in mice transplanted with MKN45 gastric cancer cells under various treatments, including PBS, C9‐MG1655‐CR alone, Nb289‐MG1655‐CR alone, MG1655‐C9‐CR plus irradiation (L: 808 nm laser irradiation), and Nb289‐MG1655‐CR plus irradiation (n = 6 per group). D) Individual tumor curves from C. E) Survival analysis of mice under various treatments, including 1, PBS, 2, MG1655‐C9‐CR plus irradiation (L: 808 nm laser irradiation), and 3, Nb289‐MG1655‐CR plus irradiation (n = 5 per group). F) Immunohistochemical staining (left) and quantification (right) of TUNEL and Ki67 in tumor tissues collected from (C). Scale bars, 40 µm. G) Tumor growth curves from mice with subcutaneous transplantation of ASPC1 pancreatic cancer cells under various treatments, including PBS, MG1655‐C9‐CR plus irradiation (L: 808 nm laser irradiation), and Nb289‐MG1655‐CR plus irradiation (n = 5 per group). H) Individual tumor growth curves of pancreatic tumors from g. I) Survival analysis of mice under various treatments in ASPC1 pancreatic cancer model related to g; 1, PBS, 2, MG1655‐C9‐CR plus irradiation (L: 808 nm laser irradiation), and 3, Nb289‐MG1655‐CR plus irradiation (n = 5 per group). *P < 0.05, **P < 0.01, and ***P < 0.001.

We have demonstrated that nanobody‐engineered MG1655 exhibits great tumor‐homing ability towards CDH17‐expressing cancer models, and the biohybrid bacteria (Nb289‐MG1655‐CR) exhibit an excellent killing effect on CDH17‐positive cancer cells. Further, we explored whether the biohybrid bacteria could also exert outstanding in vivo tumor suppression. Thermal imaging data collected 24 h after intravenous administration of 5 × 10^6^ CFU Nb289‐MG1655‐CR and C9‐MG1655‐CR into MKN45 gastric cancer‐bearing mice suggested that the local temperature in tumor tissues receiving treatment with Nb289‐MG1655‐CR rose to 50 °C within 4 min upon irradiation with an 808 nm laser (0.8 W cm^−2^). In contrast, the tumor temperature in mice treated with C9‐MG1655‐CR was only slightly elevated to 40 °C (**Figure** **6**A). This indicates that the Nb289‐MG1655‐CR biohybrid bacteria can abundantly accumulate in the tumor area through CDH17 binding to achieve effective temperature elevation upon irradiation. Furthermore, based on previous in vivo imaging results in MKN45 and ASPC1‐induced tumor models, we developed an experimental protocol for intravenous injection of biohybrid bacteria followed by a one‐time irradiation 24 h later (Figure 6B). We found no significant difference in tumor growth among the groups of Nb289‐MG1655‐CR alone, C9‐MG1655‐CR alone, C9‐MG1655‐CR plus irradiation, and PBS control. Only tumors in mice treated with Nb289‐MG1655‐CR plus irradiation were significantly suppressed after a single irradiation (Figure 6C–E). Subsequently, survival analysis also indicated that the biohybrid bacteria Nb289‐MG1655‐CR plus irradiation significantly prolonged the survival of mice compared to PBS and C9‐MG1655‐CR plus irradiation (Figure 6F). Immunohistochemical staining in tumor tissues after various treatments also indicated that treatment with MG1655‐Nb289‐CR plus irradiation induced abundant cell apoptosis compared to control groups, which is consistent with the results from H&E staining showing noticeable cell death and damage in tumor tissues (Figure 6G). In contrast, the expression of Ki67 was significantly decreased in the tumor tissues treated with Nb289‐MG1655‐CR plus irradiation compared to the other control groups (Figure 6G). These results suggest that targeted biohybrid bacteria MG1655‐Nb289‐CR plus irradiation can effectively induce tumor cell apoptosis and suppress tumor proliferation in vivo. We also did not observe any noticeable side effects of the treatment with MG1655‐Nb289‐CR plus irradiation based on mouse body weight during the entire therapeutic period, and on the blood biochemistry parameters and histological analysis of important control organs (Figures S20–S22, Supporting Information), indicating that PTT mediated by biohybrid bacteria is a safe and applicable therapeutic modality for cancers. As mentioned earlier, pancreatic cancer is refractory to most therapeutic modalities, including immunotherapy, due to its unique microenvironment.^[^ ^26^ ^]^ We showed that engineered bacteria Nb289‐MG1655 can effectively accumulate in ASPC1 pancreatic tumor tissues within 24 h compared to bacteria modified with an irrelevant nanobody C9. To further demonstrate the therapeutic effect of Nb289‐MG1655‐CR plus irradiation in pancreatic cancer, mice with ASPC1 pancreatic tumors were irradiated 24 h after the administration of biohybrid bacteria. Notably, Nb289‐MG1655‐CR plus irradiation showed promising efficacy in inhibiting ASPC1 pancreatic cancer and significantly extended the survival of mice compared to C9‐MG1655‐CR plus irradiation or vehicle control (Figure 6H–J), suggesting that CDH17 nanobody Nb289 markedly enhances the penetration of MG1655 bacteria conjugated with CR. These results demonstrate that the biohybrid bacteria engineered with CDH17 targeted nanobodies and the photothermal sensitizer CR can effectively home to tumor tissues highly expressing the target protein CDH17 and can significantly inhibit tumor growth, even in refractory pancreatic cancer.

### 2.7 Nb289‐MG1655‐CR Plus Irradiation Delays Colorectal Cancer Growth and Programs TME in Immunocompetent Mice

We have demonstrated that biohybrid bacteria Nb289‐MG1655‐CR can effectively penetrate CDH17‐expressing gastric and pancreatic tumors transplanted in immunocompromised mice. Further, considering that Nb289 could recognize both human and murine CDH17 proteins with similar affinity, we investigated whether Nb289‐MG1655‐CR could also accumulate in highly expressing murine CDH17 tumors in syngenetic and immunocompetent mice. Additionally, we aimed to determine if Nb289‐MG1655‐CR plus irradiation could have an impact on the TME by stimulating exogenous materials released from lysed bacteria upon heating.^[^ ^27^ ^]^ For this, we selected Colon26 colorectal cancer cells derived from BALB/c mice, which highly express murine CDH17, to establish a syngeneic tumor model in immunocompetent BALB/c mice for targeted imaging and therapy using Nb289‐MG1655‐CR bacteria.

**Figure 7:**
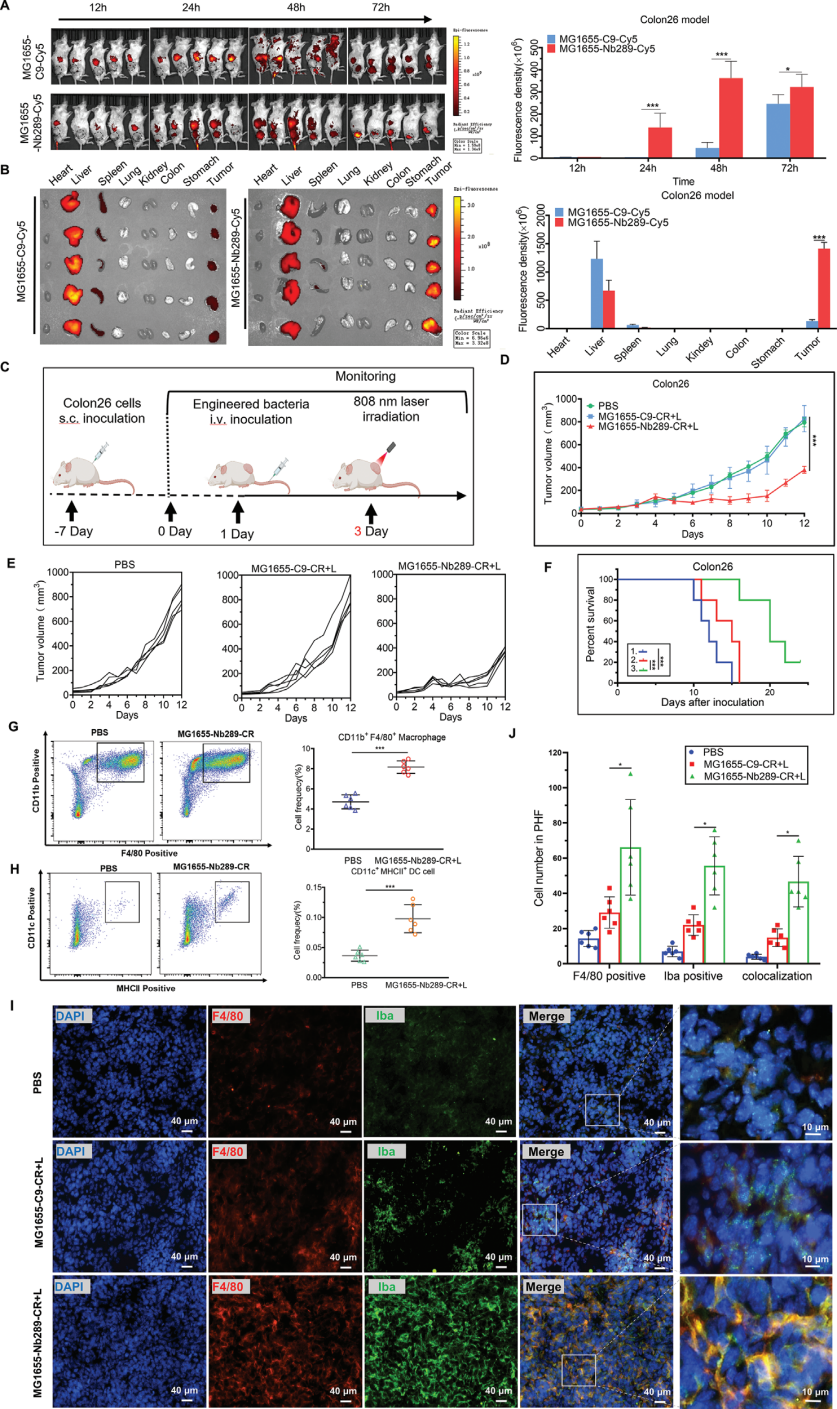
MG1655‐Nb289‐CR mediated photothermal therapy suppresses murine colorectal tumor growth and increases macrophage infiltration in immunocompetent mice. A) In vivo imaging analysis of the murine colorectal tumors with Cy5‐labeled Nb289‐MG1655 in Colon26 tumor‐bearing mice. C9‐ and Nb289‐engineered bacteria were labeled with Cy5 dye, and imaging was performed at different time points (12, 24, 48, and 72 h) after bacterial injection (5×10^6^ CFU per mouse, n = 4 per group). Immunocompetent BALB/c mice were used in this procedure. B) Ex vivo imaging analysis of the major organs collected from a after 72 h circulation (n = 4 per group). C) Treatment schedule for biohybrid bacteria plus irradiation in Colon26 tumor‐bearing mice. D) Tumor growth curves of Colon26 tumors under various treatments, including 1, PBS, 2, C9‐MG1655‐CR plus irradiation (L: 808 nm laser irradiation, 0.8 W cm^−2^, 5 min), and 3, Nb289‐MG1655‐CR plus irradiation (n = 5 per group). E) Individual tumor growth curves of Colon26 tumors from d. F) Survival analysis for Colon26 tumor‐bearing mice 1, 2 and 3 groups from d (n = 5 per group). G) Flow cytometry analysis of infiltrated macrophages in tumor tissues following treatment with PBS and Nb289‐MG1655‐CR plus irradiation. Tumor tissues were collected and analyzed on day 4 after laser irradiation (n = 6 per group). H) Flow cytometry analysis of infiltrated mature dendritic cells in tumor tissues following treatment in the PBS and Nb289‐MG1655‐CR plus irradiation groups (n = 6 per group). I and J) Immunofluorescence analysis and quantification of infiltrated macrophages and macrophage phagocytosis by F4/80 and Iba staining in tumor tissues from g. Scale bars, 40 µm (left), 10 µm (right). *P < 0.05, **P < 0.01, and ***P < 0.001.

First, we examined the targeting ability of Cy5‐labeled Nb289‐engineered MG1655 in Colon26 tumor‐bearing mice. The results showed that Cy5‐labeled MG1655‐Nb289 could be observed in the tumor sites 24 h after intravenous injection but took longer to reach the peak (48 h after circulation). This differs from gastric and pancreatic cancer models, where bacteria showed the highest accumulation 24 h after circulation (**Figure** **7**A). Ex vivo imaging of dissected organs after 72 h of circulation yielded similar results to those of immunodeficient nude mice with gastric and pancreatic tumors. The enrichment of Nb289‐MG1655 in tumors was much stronger than that of the C9‐MG1655 control group, and the accumulation of Nb289‐MG1655 in liver tissues was lower than in C9‐MG1655 (Figure 7B). These results indicate that Nb289‐MG1655 also exhibits excellent homing ability to Colon26 tumors with murine CDH17 expression but requires more time to reach the plateau compared to the other two tumor models. This difference might be due to variations in tumor heterogeneity or the effects of a normal immune system. Subsequently, to determine whether Nb289‐MG1655‐CR‐mediated PTT could also inhibit murine colorectal tumor growth in immunocompetent mice, Colon26 tumors were irradiated with an 808 nm laser at 48 h after the administration of biohybrid bacteria based on the imaging study (Figure 7C). Mild‐temperature PTT (≈45 °C) was performed to favor microenvironmental regulation and minimize damage to immune cells during tumor treatment.^[^ ^28^ ^]^ The treatment results showed that Nb289‐MG1655‐CR plus irradiation (45 °C) considerably inhibited tumor growth and prolonged the survival of mice in the Colon26 tumor model, similar to the effects observed in the MKN45 gastric and ASPC1 pancreatic cancer models. Importantly, no evident changes in body weight were observed during the entire therapeutic period (Figure 7D–F; Figure S23, Supporting Information). However, some tumors resumed growth at a later stage due to the intrinsic thermo‐resistance of cancer cells and the mild‐temperature photothermal treatment, unlike the 50 °C used in gastric and pancreatic cancers. Nonetheless, increasing evidence suggests that mild‐temperature PTT can improve anti‐tumor immunity. To investigate the effect of Nb289‐MG1655‐CR‐mediated PTT on the tumor immune microenvironment, immune cell analysis was conducted on the fourth day after photothermal treatment. The analysis revealed that the infiltration of dendritic cell (DC) cells (CD11c^+^/MHCII^+^) and macrophages (CD11b^+^/F4/80^+^) in tumor tissues treated with Nb289‐MG1655‐CR plus irradiation was significantly increased, especially of macrophages, which were abundantly induced from 4% to 8% (Figure 7G,H; Figure S24, Supporting Information). However, the infiltration of CD3^+^CD4^+^ and CD3^+^CD8^+^ T cells only showed a slight increase without significant differences compared to the control group (Figure S25, Supporting Information). The increased macrophage infiltration was further confirmed by F4/80 staining, and meanwhile, the enhancement of macrophage phagocytosis was also verified with Iba antibody immunofluorescence staining in tumor tissues (Figure 7I,J). Notably, we did not observe a clear phenotype change in macrophages, such as M2 to M1, despite the marked recruitment of macrophages in tumor tissues after treatment (data not shown). Collectively, these findings suggest that mild‐temperature PTT mediated by biohybrid bacteria targeting CDH17 can partially ablate murine tumor growth. Importantly, it can also prime the TME and significantly induce the infiltration and phagocytosis of macrophages, which could be pivotal targeting immune cells for checkpoint blockade therapy in the context of bacteria‐based PTT.

### 2.8 STING Pathway is Activated in Infiltrated Macrophages, and CD47 Nanobody Synergistically Potentiates the Anti‐Tumor Efficacy of MG1655‐Nb289‐CR Plus Irradiation

**Figure 8:**
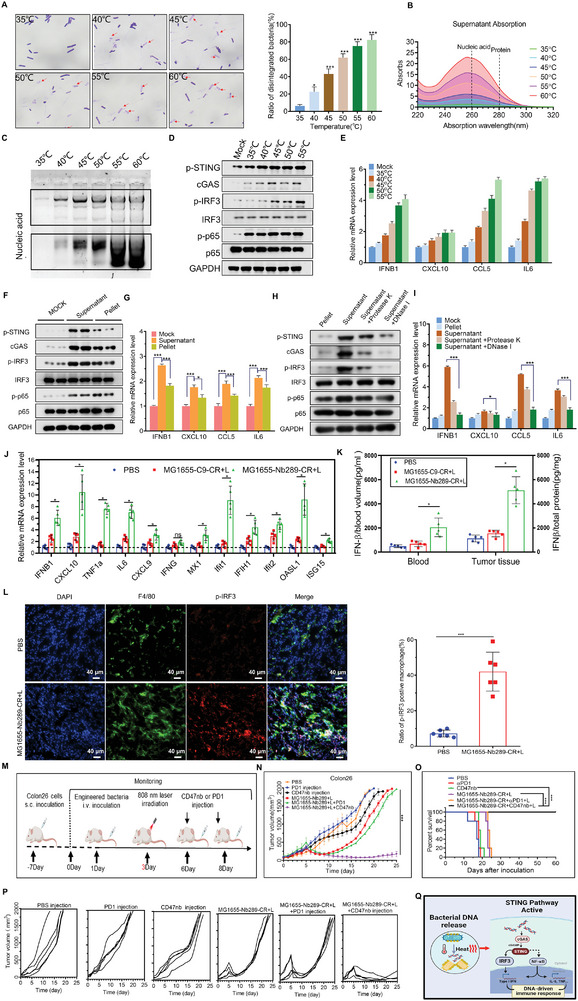
Nb289‐MG1655‐CR mediated PTT can activate the STING pathway in macrophages and facilitate immunotherapy with CD47 nanobody. A) Crystalline violet staining and quantification for engineered MG1655 after heating with different temperatures at 35–60 °C. Unstained bacteria indicated by red arrows were disintegrated bacteria (n = 6 per group). Scale bars, 10 µm. B) Nucleic acid and protein content in the supernatant detected using spectrophotometry. The supernatants collected from engineered bacteria treated with heat at different temperatures were assessed to detect UV absorption at wavelengths from 220–320 nm. The wavelengths indicated by the dashed lines were the absorption peaks of nucleic acids (260 nm) and proteins (280 nm). C) The nucleic acids in the supernatant from a were examined using agarose gel electrophoresis. D) The examination of the STING pathway in macrophages treated with supernatants collected from heated MG1655 using western blotting. E) Expression of target genes of the STING pathway in bacterial supernatant‐treated macrophages detected using real‐time quantitative PCR (n = 3 per group). F) Detection of the STING pathway after treatment with bacterial supernatant or pellet in isolated macrophages. Bacterial supernatant or pellet was collected from engineered MG1655 incubated at 45 °C for 5 min. G) Expression of target genes of the STING pathway in bacterial supernatant or pellet‐treated macrophages examined by real‐time quantitative PCR (n = 3 per group). H) Examination of the STING pathway in macrophages treated with preconditioned supernatants using western blotting. Bacterial supernatant was collected from engineered MG1655 incubated at 45 °C for 5 min and treated with or without DNase I or protease K to remove DNA or proteins. I) The expression of downstream genes of the STING pathway in bacterial supernatant or pellet‐treated macrophages detected by real‐time quantitative PCR (n = 3 per group). The bacterial supernatant was the same as in h. J) The expression of key transcription factors and downstream target genes involved in STING pathway activation of tumor tissue samples from animal model in (Figure 7C) detected by real‐time quantitative PCR (n = 5 per group). K) The IFNβ level of blood and tumor tissue samples from animal model in (Figure 7C) detected by ELISA. IFN‐γ concentration in tumor tissue was normalized to total protein (n = 5 per group). L) The expression of p‐IRF3 in macrophages (F4/80^+^) in tumor tissues from animal model in (Figure 7C) detected by immunofluorescence analysis (n = 5 per group). Scale bars, 40 µm. M) Treatment schedule for combinational therapy in Colon26 tumor‐bearing mice. N) Tumor growth curves under various treatments, including PBS, αPD1 (*iv*, 250 µg mouse^−1^), CD47nb (*iv*, 200 µg mouse^−1^), Nb289‐MG1655‐CR/irradiation (L: 808 nm laser irradiation), Nb289‐MG1655‐CR/irradiation plus αPD1, and Nb289‐MG1655‐CR/irradiation plus CD47nb (n = 5 per group). O) Survival analysis for tumor‐bearing mice from K. P) Individual tumor growth curves of Colon26 tumors from K. Q) Mechanism illustration of STING pathway activation in macrophages by released nucleic acids from biohybrid‐engineered bacteria after irradiation. *P < 0.05, **P < 0.01, and ***P < 0.001.

Given the finding that abundant macrophages were recruited, and phagocytosis was enhanced after biohybrid bacteria‐mediated PTT, we wondered what mechanisms were involved in anti‐tumor immunity of macrophages and whether checkpoint blockades targeting macrophages can further amplify the efficiency of bacteria‐mediated PTT. Macrophages, as an important component of innate immunity, play a crucial role in defending against bacterial invasion and can be activated through the interactions of PRRs with pathogen‐associated molecular patterns (PAMPs).^[^ ^29^ ^]^ The STING pathway has been shown to play a pivotal role in controlling bacterial infections and anti‐tumor immunity. Various STING agonists have been developed and tested in preclinical and clinical settings to treat cancers by activating the STING pathway.^[^ ^30^ ^]^ In macrophages, the STING pathway can be activated by cytosolic dsDNA stimulation, particularly DNA longer than 70 bp.^[^ ^31^ ^]^ In our study, it was evident that bacterial DNA could be released into the TME during bacteria‐mediated PTT. The elevated temperature not only killed cancer cells but also lysed the bacteria. We found that the number of disintegrated engineered bacteria increased gradually with temperature elevation from 35–60 °C (**Figure** **8**A). The concentration of nucleic acids and proteins in the supernatant from heat‐treated engineered bacteria also significantly increased with higher incubation temperatures (Figure 8B). Agarose gel electrophoresis further confirmed the increase in nucleic acids in the supernatant (Figure 8C). We wondered whether the exogenous DNA released from lysed bacteria could stimulate the STING pathway in macrophages in vitro and activate the STING pathway in tumor‐associated macrophages in the TME to exert anti‐tumor immunity.

To investigate this, macrophages isolated from the peritoneal cavity of thioglycolate‐stimulated mice (Figure S26) were treated with supernatants collected from bacteria heated at different temperatures. The levels of key proteins involved in STING activation, such as phosphorylated STING and IRF3, and cGAS, increased with increasing incubation temperatures of the bacteria. Additionally, the phosphorylation of the p65 protein, which is an overlapping signal molecule (NF‐κB) between the STING pathway and other pathways related to PRRs, such as Toll‐like receptors (TLRs), also increased but not in a temperature‐dependent manner. This suggests that the supernatants from lysed bacteria specifically stimulate the STING‐IRF3 axis responsible for the production of type I interferons (IFNs) (Figure 8D). The expression levels of targeted downstream genes associated with STING activation and the subsequent production of type I IFNs, such as IFNB1, CCL5, CXCL10, and IL6, also increased in a temperature‐dependent manner (Figure 8E). The translocation of the transcription factor IRF3 from the cytoplasm to the nucleus after phosphorylation, a crucial step for the production of type I IFNs after STING pathway activation, was observed, with gradual nuclear accumulation of IRF3 corresponding to increasing temperatures (Figure S27, Supporting Information). Notably, the translocation of the p65 protein to the nucleus could be observed after treatment with bacterial supernatants, but no gradual accumulation in the nucleus was observed with temperature changes, which is consistent with the phosphorylation data of p65 (Figure S28, Supporting Information). To confirm whether supernatant from lysed bacteria could activate the STING pathway to a greater extent than the pellet collected from bacteria after heat treatment, macrophages were treated with supernatant, and pellet isolated from bacteria after heat treatment at 45 °C. The results showed that the supernatant could activate the STING pathway in macrophages more effectively than the pellet based on the expression of marker proteins and targeted downstream genes (Figure 8F,G). To confirm that DNA but not proteins in the supernatants was mainly responsible for STING activation, we treated the supernatants with proteinase K and DNase before incubation with macrophages. The results showed that DNase treatment almost abolished the activation of the STING pathway and reduced the expression levels of related genes, whereas proteinase K only partially inhibited STING pathway activation and related gene expression (Figure 8H,I). This indicates that DNA is more important than proteins in the supernatant, and specific proteins may facilitate STING activation by forming complexes with DNA.^[^ ^32^ ^]^ To confirm these finding in tumor tissues, the STING pathway‐associated gene panel was determined in the mouse tumor tissues from PTT‐treated animals in Figure 7C, showing that Nb289‐MG1655‐CR mediated PTT led to significant upregulation of key transcription factors involved in STING pathway activation and downstream target genes when compared with PBS or C9‐MG1655‐CR (Figure 8J). Additionally, the levels of IFNβ in blood and tumor tissues detected by ELISA also significantly increased in Nb289‐MG1655‐CR‐treated tumors (Figure 8K). Consistently, tumor tissue staining revealed that p‐IRF3 was significantly upregulated in tumor‐associated macrophages in tumors treated with Nb289‐MG1655‐CR‐mediated PTT (Figure 8I), indicating the activation of the STING pathway in macrophages. Since the elevated temperature in tumor site induced by engineered bacteria‐mediate PTT not only resulted in the release of bacterial DNA but also destroyed the cancer cells which might also unleash cellular DNA for STING activation, we further explored whether cellular DNA released from destroyed cancer cells can stimulate STING pathway and compared the activation extent for both types of DNAs. We found that the supernatants from heated bacteria could more prominently activate STING pathway in macrophages than those from the same number of heated cells (Figure S31, Supporting Information) although the latter could also obviously stimulate STING pathway, suggesting that bacteria are mainly responsible for the activation of STING pathway in bacteria‐mediated PTT, but cellular DNA from destroyed cancer cells might also partially contribute to the activation. Collectively, these results suggest that biohybrid‐engineered bacteria, under photothermal irradiation, can activate the STING pathway in macrophages by released nucleic acids from bacteria and possibly, in part from destroyed cancer cells, into the TME. This ultimately promotes the infiltration and activation of macrophages and the maturation of DC cells, contributing to anti‐tumor immunity.

CD47 blockade can enhance the anti‐tumor efficacy of STING agonists.^[^ ^33^ ^]^ Based on our findings of macrophage infiltration and STING activation in tumor‐associated macrophages (TAMs) after treatment, we further investigated whether combining biohybrid bacteria‐mediated PTT with CD47 nanobody could synergistically inhibit tumor growth. We utilized a well‐documented CD47 nanobody^[^ ^34^ ^]^ (Figure S32, Supporting Information), which is reportedly ineffective as a monotherapy for tumor growth, for the combination therapy (Figure 8M). As a control, we also designed a combination therapy with PD‐1 antibody to compare with the CD47 nanobody combination treatment. Consistent with previous studies, CD47 nanobody alone or PD‐1 antibody alone did not inhibit Colon26 tumor growth compared to the vehicle control.^[^ ^35^ ^]^ Although PTT alone significantly delayed tumor growth, tumors eventually regrew (Figure 8N–P). The combination therapy of PD‐1 antibody with PTT showed similar anti‐tumor efficacy to PTT alone, indicating that T‐cell infiltration is a prerequisite for the tumor inhibition of PD‐1 antibody since we did not observe significant T‐cell recruitment after PTT with biohybrid bacteria (Figure 8N; Figure S25, Supporting Information). Expectedly, the addition of CD47 nanobody to the PTT regimen significantly suppressed tumor development; all tumors in this group were eradicated and did not regrow until the end of the experiment (60 days) (Figure 8N–P). Survival analysis also confirmed these results, as all mice treated with biohybrid bacteria‐mediated PTT plus CD47 nanobody survived until the end of the experiment. The addition of PD‐1 antibody did not noticeably extend the survival of mice compared to PTT alone, which aligns with the lack of T‐cell infiltration in the TME after PTT (Figure 8O; Figure S25, Supporting Information). To further verify that STING pathway activation plays a determinant role in the tumor suppression resulted from the photothermally engineered bacteria therapy combined with CD47 nanobody, we cotreated the Colon26 tumor bearing mice with a STING pathway inhibitor H151 on the basis of bacteria‐mediated PTT combined with CD47 nanobody (Figure S33, Supporting Information). H151 alone could significantly suppress STING activation in vitro, and it did not produce any inhibitory effect on tumor growth (Figure S33A,B, Supporting Information). Whereas H151 treatment could significantly reverse the tumor suppressive effect of engineered bacteria‐mediated PTT plus CD47 nanobody (Figure S33C, Supporting Information), which clearly demonstrates the critical role of the STING pathway in this combinational therapy regimen. Collectively, these results demonstrate that our CDH17 nanobody‐engineered bacteria MG1655, conjugated with the photothermal sensitizer CR, can activate the STING pathway in TAMs by releasing bacterial DNA upon irradiation at the tumor site. This process leads to increased infiltration of macrophages in the TME. Additionally, the combination of checkpoint blockade using CD47 nanobody can synergistically enhance the anti‐tumor effect of PTT mediated by biohybrid‐engineered bacteria (Figure 8Q).

## 3 Discussion

In this study, we developed a biohybrid bacterial system for PTT in GI cancers with high expression of CDH17. This system is involved in modifying the non‐pathogenic bacteria MG1655 with CDH17 nanobodies on the surface and conjugating them with the photothermal sensitizer CR. The resultant biohybrid bacteria exhibited effective targeting toward gastric, pancreatic, and colorectal cancers, all of which highly expressed CDH17 to varying degrees. Photothermal irradiation rapidly increased the temperature at the tumor sites and inhibited tumor progression, as described earlier. Importantly, PTT mediated by this biohybrid bacterial system also influenced the TME in a syngeneic colorectal cancer model in immunocompetent mice. The STING pathway was activated in TAMs by releasing nucleic acids from lysed bacteria, leading to increased macrophage recruitment in the TME without significant T‐cell infiltration. Furthermore, the addition of CD47 nanobody to the bacterial PTT regimen showed maximal anti‐tumor efficacy and prolonged survival.

CDH17 has been extensively studied as a promising biomarker and therapeutic target for digestive system cancers, including gastric, pancreatic, and colorectal cancers.^[^ ^11^, ^12^ ^]^ However, concerns have been raised regarding potential off‐target effects on normal intestinal epithelia, which also express CDH17 protein. Our data, along with another report,^[^ ^12c^ ^]^ reveal that nanobody‐based approaches, including nanobodies, nanobody‐engineered bacteria, or nanobody‐based CAR T cells targeting CDH17, do not reach intestinal epithelia with high CDH17 expression (Figures 1, and 3; Figures S11 and S15, Supporting Information). CDH17 staining in a normal intestinal tract showed high expression at the lateral tight junction between epithelial cells, whereas expression in the luminal and basal layers was rare. This limited accessibility of CDH17 outside of tumor cells, which exhibit high CDH17 expression throughout their cell surface, may explain why drugs have difficulty accessing CDH17 in normal intestinal epithelia (Figure S11, Supporting Information).^[^ ^12c^ ^]^ Although CDH17 targeting has gained attention in recent years, most studies have focused on conventional monoclonal antibodies, Fab or scFv fragments for drug development.^[^ ^12^, ^36^ ^]^ The exploration of nanobodies specifically targeting CDH17 has been limited. Nanobodies, or VHHs, are the smallest naturally occurring antibody fragments engineered from heavy‐chain‐only antibodies found in camelids and cartilaginous fishes.^[^ ^37^ ^]^ Owing to their small molecular mass (≈15 kDa) and high binding affinity for targets, nanobodies are easily expressed and purified in bacteria, rendering them ideal for drug development.^[^ ^38^ ^]^ Since the approval of the first nanobody drug, caplacizumab, by the FDA in 2019 for the treatment of acquired thrombotic thrombocytopenic purpura,^[^ ^39^ ^]^ various nanobodies for cancer, autoimmune diseases, respiratory diseases, hematologic diseases, and more have entered clinical trials.^[^ ^40^ ^]^ In our previous study, we identified two nanobodies (A1 and E8) that bind to human CDH17 protein through nanobody phage library screening followed by phage ELISA. However, they unfortunately did not recognize murine‐derived CDH17, limiting their applications in syngeneic mouse tumors. Considering the high homology between human and murine CDH17 proteins (Figure S6, Supporting Information), we hypothesized that the nanobody phage sub‐library against human CDH17 might contain clones capable of binding to murine CDH17. However, they were not identified using phage ELISA, likely due to the limited number of clones analyzed (≈200). In this study, we employed deep sequencing technology to analyze millions of nanobody sequences and ranked them based on frequency (Figure 1).^[^ ^41^ ^]^ Interestingly, the top two sequences were identical to A1 and E8, which were initially identified using phage ELISA, confirming the reliability of our approach. We further purified nine nanobodies from the top ten sequences. ELISA data showed that these nanobodies could bind to both human and murine CDH17, and their activity surpassed that of the primary A1 and E8 nanobodies. Notably, Nb289 and Nb535, which exhibited high homology with A1, not only bound to human and murine CDH17 proteins but also displayed higher binding affinity than A1 for human CDH17 (Figure 1; Figure S8, Supporting Information).^[^ ^11c^ ^]^ Subsequent applications of Nb289 confirmed its recognition of murine CDH17 highly expressed in the syngeneic colorectal cancer cell line, Colon26, both in vitro and in vivo. Collectively, we demonstrate that combining nanobody phage library screening with deep sequencing appears to be an efficient strategy for identifying rare positive clones that might be missed by conventional phage ELISA.

Based on of bacterial intrinsic tropism toward hypoxic/necrotic zone in tumor mass and their potent capacity for payload delivery (or biological molecular synthesis), non‐pathogenic or probiotic bacteria‐mediated therapy in cancers is attracting increasing interest.^[^ ^2^, ^6^, ^10^, ^42^ ^]^ Although bacteria could preferentially colonize and proliferate in hypoxic and necrotic areas of tumors, ample evidence has suggested that bacteria typically require 72 h to reach peak density in tumors and can be readily phagocytosed by the liver and spleen after intravenous administration. Furthermore, bacteria administered intravenously can transiently accumulate in normal organs.^[^ ^2^, ^8^, ^43^ ^]^ Facultative anaerobic bacteria, on the other hand, can not only colonize tumor masses and but also proliferate in oxygen‐enriched normal organs.^[^ ^4^ ^]^ Therefore, unmodified natural or attenuated bacteria pose potential risks for cancer therapy due to non‐specific uptake by the liver and spleen, as well as transient accumulation in normal organs. In this study, we engineered the non‐pathogenic bacteria MG1655 by introducing CDH17 nanobodies onto their surface through fusion with the C‐terminal fragment of EHEC intimin to enhance bacterial targeting efficiency to tumors.^[^ ^17^ ^]^ Our data demonstrated that, in three tumor models, MG1655 engineered with Nb289 could rapidly and abundantly home to CDH17‐positive tumors, at least 24 h earlier than unmodified MG1655. This targeting efficiency was observed even in highly fibrotic pancreatic cancer, where engineered MG1655 colonized the tumors within 24 h, whereas unmodified MG1655 was almost undetectable. Moreover, targeted MG1655 showed significantly reduced accumulation in normal liver tissues. These results indicate that engineered bacteria with targeting ligands significantly improve their selectivity for tumor tissues, allowing for more effective payload delivery and drug synthesis. Additionally, bacteria endowed with tumor‐homing ability can significantly reduce non‐specific uptake by normal organs, thereby improving biosafety for in vivo applications of bacteria and increasing the potential for clinical translation.

Non‐pathogenic or attenuated bacteria used as monotherapy have shown limited anti‐tumor effects, whether administered systemically or intratumorally.^[^ ^6b,c^ ^]^ To enhance their therapeutic potential, various components have been combined with bacteria for payload delivery or genetic manipulation to produce anti‐tumor agents. Bacteria have been conjugated with a wide range of payloads, demonstrating excellent anti‐tumor performance. Moreover, advancements in synthetic biology have facilitated the engineering of bacteria to produce diverse anti‐tumor biological agents, such as nanobodies, cytokines, chemokines, and small molecules.^[^ ^4^, ^9^, ^42^, ^43^ ^]^ In our study, we conjugated nanobody‐engineered MG1655 bacteria with the CR dye, efficiently generating a photothermal effect upon irradiation. We demonstrated that the biohybrid bacteria (Nb289‐MG1655‐CR) specifically accumulated in CDH17‐positive tumors, including fibrotic pancreatic cancer, and significantly inhibited tumor growth without evident side effects after a single administration and one‐time irradiation (Figures 4 and 6). Biomaterial‐mediated mild PTT has been shown to shape the TME primarily by activating adaptive immunity and enhancing the efficacy of immunotherapies using PD‐1 and PD‐L1 antibodies.^[^ ^28^, ^44^ ^]^ Interestingly, we found that Nb289‐MG1655‐CR combined with irradiation remarkably induced the infiltration of macrophages and promoted DC maturation, without a significant increase in T‐cell infiltration in a syngeneic murine Colon 26 tumor model (Figure 7). This differs from other studies on PTT for TME reprogramming. The key factor in our study is the introduction of engineered MG1655 bacteria that can be lysed upon irradiation. The release of abundant exogenous materials, including nucleic acids and tumor antigens, from the heated bacteria and cancer cells may synergistically activate macrophages, possibly through the STING pathway,^[^ ^32^ ^]^ thereby recruiting more macrophages to the TME and promoting DC maturation. Previously, Chowdhury et al. constructed an *E. coli* synchronized circuit system (eSLC) in which probiotic *E. coli* (EcN 1917) could colonize tumors and automatically break down through quorum lysis to simultaneously release CD47 nanobodies.^[^ ^2a^ ^]^ However, eSLC treatment alone, without nanobody release, did not significantly increase macrophage infiltration and the phenotype alteration in macrophage was only observed when CD47 nanobody was incorporated into this system.^[^ ^2a^ ^]^ These observations differ between our system and theirs, where the disintegrated materials from lysed bacteria seem unable to activate the innate response, possibly due to different bacterial strains or the absence of tumor antigens released upon irradiation in our system. Therefore, engineered bacteria‐mediated PTT may represent a robust modality for modulating the TME, particularly by enhancing innate immunity for cancer therapy.

STING agonists, which activate the innate immune pathway, have been extensively studied in various preclinical and clinical settings, often combined with other therapeutic strategies, yielding promising results in preclinical studies. The STING pathway plays a crucial role in immune defense against bacterial/viral infections and tumors by inducing the production of type I IFNs and other cytokines/chemokines, such as TNFα and CXCL9/10.^[^ ^45^ ^]^ In our study, we revealed that bacterial DNA released from heat‐treated bacteria could activate the STING‐IRF3 axis in macrophages in vitro and in vivo, leading to the production of type I IFN‐associated factors and enhanced phagocytosis of macrophages (Figures 7 and 8). TLRs, such as TLR4/TLR5, are typically activated by bacterial components, including LPS and flagellin, to exert anti‐tumor immunity. However, non‐pathogenic attenuated MG1655 bacteria, genetically modified in lethal genes, may only exhibit basal activation of the TLR system, as indicated by the phosphorylation of p65, which was not increased in the stimulation of supernatants collected from bacteria at higher temperatures. Conversely, STING pathway activation showed temperature‐dependent increase. These findings suggest that STING activation may be the primary trigger for TME reprogramming. STING activation in macrophages has been shown to prime antigen‐presenting cells, such as DCs, for maturation and enhance macrophage phagocytosis,^[^ ^46^ ^]^ which is consistent with our findings. Kosaka et al. reported that CD47 inhibition greatly enhances the anti‐tumor effect of a STING agonist through macrophage phagocytosis of tumor cells.^[^ ^33^ ^]^ This finding is in line with our results. In our study, the abundant macrophage recruitment after Nb289‐MG1655‐CR‐mediated PTT, coupled with the addition of CD47 nanobody, led to almost complete eradication of tumors, indicating a significant enhancement of macrophage function. However, the PD‐1 antibody did not show obvious therapeutic benefits, confirming the lack of T‐cell infiltration. The syngeneic Colon26 colorectal cancer model used in our study has low immunogenicity and few infiltrating T cells under normal conditions, resulting in a limited response to PD‐1/PD‐L1 checkpoint blockade. Therefore, enhancing macrophage infiltration through bacteria‐mediated PTT in tumors with a cold TME and low immunogenicity, combined with CD47‐SIRPα axis immune checkpoint blockade in macrophages, may serve as an alternative to T‐cell checkpoint blockade.

Notably, although we tested the biohybrid‐engineered bacteria in three tumor models with high CDH17 expression, and examined their anti‐tumor performance and mechanisms, we only conducted TME reprogramming experiments in a syngeneic colorectal murine tumor model in immunocompetent mice. Further studies are needed to determine if these findings are applicable to other syngeneic models. For example, it would be valuable to investigate whether this therapeutic strategy can produce similar benefits and operate through the same mechanisms in lung and liver metastasis models derived from colorectal cancer. Additionally, it would be worthwhile to evaluate the response of a pancreatic cancer model with high CDH17 expression in immunocompetent mice to bacteria‐mediated PTT combined with CD47 nanobody. Furthermore, in this study, we primarily focused on macrophages since they are abundant in the TME and significantly recruited after treatment. It is possible that the STING pathway in DCs, which were also induced after treatment, could be activated and synergistically enhance anti‐tumor innate immunity; nevertheless, this requires further exploration.

## 4 Experimental Section

### Phage Display Combined with Deep Sequencing for the Discovery of CDH17 Nanobodies

The phage display screening process was described in detail in this previously.^[^ ^11c^ ^]^ Here, the phage mixture obtained after third‐round screening was applied for further deep sequencing. Genomic DNA was extracted from the phage mixture by Genome Extraction Kit (TIANGEN, China), and the nanobody sequences were amplified with the primers (forward primer: 5′ ATGGCGGTGCAGCTGGTGGAGTCT 3′, reverse primer: 5 TTGGCCTCCCGGGCCGCGTGCGCC3'). The amplified products were run on an agarose gel and the target bands were purified by DNA gel purification kit (TIANGEN, China) and the purified DNA products were used for high‐throughput sequencing. The reverse reads were complemented and merged with the forward reads. Reads containing both primers were extracted to translate into protein sequences. The sequences that terminate prematurely and cannot be translated into protein were removed, and the correct sequences were then matched according to the protein sequences corresponding to the primers. The number of reads corresponding to the same nanobody protein sequence was statistically analyzed.

### Cell Lines and Cell Culture

In this study, a variety of gastric, colon and pancreatic cancer cells were used. The cell lines were obtained from the American Type Culture Collection (ATCC, Manassas, VA); Cell Bank of Chinese Academy of Sciences (Shanghai, China) and Fuheng Biology (Shanghai, China). The culture conditions were used as follows. MKN45, TMK1, ASPC1, Colon26, HCT116, MC38 and AGS cells were cultured in RPMI1640 (Gibco, USA) supplemented with 10% fetal bovine serum (FBS), 2 mM L‐glutamine. IM95 and Panc02 were maintained in high‐glucose Dulbecco's Modified Eagle's Medium (DMEM) supplemented with 10% FBS, 2 mM L‐glutamine. All the cell lines were incubated at 37 °C in a humidified atmosphere containing 5% CO_2_.

### Mouse Xenograft Models and Treatments

All experiments on mice in the present study were done following the approved protocol by Institutional Animal Care and Use Committee (IACUC) of the Shenzhen People's hospital (Accreditation number: AUP‐220501‐LZJ‐0595‐01) and were carried out in accordance with relevant institutional and national guidelines and regulations. BALB/c and BALB/c nude mice at 8 weeks old were purchased from Gempharmatech (Guangzhou, China) and were maintained under pathogen‐free conditions in the animal center of the Shenzhen People's hospital. Mice were euthanized when showed obvious signs of discomfort or when maximal tumor size reached 2000 mm^3^.

Gastric cancer cells MKN45 (4 × 10^6^ cells), pancreatic cancer cells ASPC1(5× 10^6^ cells) or murine colon cancer cells Colon26(1 × 10^6^ cells) were suspended in 100 µL PBS and injected subcutaneously into the right flank of mice. Tumor size was measured with vernier calipers and calculated using the following formula: (length × width^2^)/2.

To determine the therapeutic effect of photothermally engineered bacteria, MKN45 tumor‐bearing mice were divided into 6 groups and injected with 100 µl of PBS, MG1655‐C9(0.5×10^7^ CFU) and MG1655‐NB289 (0.5×10^7^ CFU) in the tail vein at a tumor size equal to 100 mm^3^. The mice were then received light or dark treatment with 0.8 W cm^−2^ 808 nm laser for 10 min after 24 h. During treatment, tumor size and body weight in mice were monitored. For survival study, mice were killed when tumor size reached 2000 mm^3^ after treatment, the major organs including heart, liver, spleen, lung, kidney, and tumor were collected. Frozen sections were prepared and analyzed by H&E staining, Ki67 and TUNEL staining. In the ASPC1 and Colon26 tumor models, tumor‐bearing mice were randomly divided into 3 groups. Mice were then intravenously injected with PBS, C9‐MG1655‐CR (0.5×10^7^ CFU) and Nb289‐MG1655‐CR (0.5×10^7^ CFU) at a tumor size equal to 100 mm3 and treated with 0.8 W cm^−2^ 808 nm laser light treatment for 10 min after 24 or 48 h. During treatment, tumor size and body weight in mice were monitored. For survival study, mice were killed when tumor size reached 2000 mm^3^ after treatment.

### Protein Purification

The CDH17 protein fragments and various CDH17 nanobodies were purified as described previously.^[^ ^11^, ^19^ ^]^ For the expression and purification of target protein, the recombinant plasmids pET‐14B‐CDH17‐domain 1–3(human and mouse) were transformed into BL21(DE3) and then the bacterial clones were incubated at 37 °C and 225 rpm until reaching 0.6 of OD600 value. The cultures were induced with 0.2 mM IPTG at 16 °C and 225 rpm overnight. The cultures were then pelleted by centrifugation at 8000 g for 15 min at 4 °C. Cell pellets were dissolved in lysis buffer (300 mM NaCl, 50 mM NaH_2_PO_4_, 10 mM imidazole, pH 8.0, 1 mM PMSF) and crushed at low temperature and high pressure for 3 times. The lysate was spun down for 45 min at 12 000 × g, and the supernatants were loaded on a gravity column containing 1 ml Ni‐NTA agarose resin (Qiagen, Germany). The protein–bound resin was washed with 50 ml Wash Buffer I (300 mM NaCl, 50 mM NaH_2_PO_4_, 20 mM imidazole, pH 8.0, 1 mM PMSF),50 mL Wash Buffer II (300 mM NaCl, 50 mM NaH_2_PO_4_, 40 mM imidazole, pH 8.0, 1 mM PMSF) and then eluted with 25 ml Elution Buffer (300 mM NaCl, 50 mM NaH_2_PO_4_, 250 mM imidazole, pH 8.0, 1 mM PMSF). Finally, the eluate was fractionated by Superdex‐150 gel with AKTA Pure System (GE Healthcare Life Sciences, USA) in 1×PBS. The purified proteins were identified by SDS‐PAGE, and then quickly frozen in liquid nitrogen and restored in −80 °C until used. The nanobodies were purified by Ni‐NTA agarose resin with the same procedures. The purified nanobodies were analyzed and identified by western blot with 6×His tag, HA tag and anti‐VHH antibodies.

### Tumor Imaging In Vivo and Vitro

In this study, it was employed in vivo and in vitro imaging techniques to investigate the distribution of CDH17 nanobodies or engineered bacteria in tumor‐bearing mice, as described in the previous studies.^[^ ^19^ ^]^

To investigate the distribution of nanobodies in vivo, the MKN45 tumor‐bearing mice were randomly divided into two groups (n = 3) and injected intravenously 100 µg Cy5‐coupled Nb289 and Con Nb respectively once tumors reached ≈500 mm^3^. At different time intervals, the mice were subjected to fluorescence scanning using an IVIS Spectrum imaging system (PerkinElmer, USA) to track the distribution of nanobodies in live mice. The mice were sacrificed at the end of the experiment, and major organs (heart, liver, spleen, lung, kidney, stomach, colon) and tumors were harvested for ex vivo fluorescence scanning.

Similarly, to investigate the distribution of engineering bacteria in vivo, the MKN45, ASPC1 or Colon26 tumor‐bearing mice were randomly divided into two groups (n = 5) and injected 0.5×10^7^ CFU Cy5‐modified engineered bacterium Nb289‐MG1655‐CR and control engineered bacterium C9‐MG1655‐CR respectively once tumors reached ≈500 mm^3^. Fluorescence scanning using the IVIS Spectrum imaging system was performed at different time intervals to monitor the distribution of bacteria in live mice. Upon completion of the experiment, the mice were sacrificed, and major organs (heart, liver, spleen, lung, kidney, stomach, and colon) and tumors were collected. A portion of tissues was used for immunohistochemical staining of anti‐VHH to further analyze the distribution of bacteria in the tissues, while the remaining portion was homogenized with 1 mL of PBS for equal amounts of wet tissue weight. Enumeration of the engineered bacteria in each tissue was performed by plating the homogenized samples.

These experiments allowed us to visualize and quantify the distribution of CDH17 nanobodies and engineered bacteria in tumor‐bearing mice, providing valuable insights into their localization within major organs and tumors.

### Flow Analysis of Immune Cell Infiltration in Tumors

To analyze the effect of engineered bacterial photothermal therapy on the immune microenvironment in tumors, the infiltration of immune cells in the tumor tissue of the Colon26 murine tumor‐bearing mouse model using spectral flow analysis was analyzed. After 96 h of photothermal treatment with engineered bacteria, the suspensions of single cells were prepared by enzymatic digestion in the tumor tissues collected from the treatment and control groups, respectively. After lysis of the cell suspension with ACK buffer, the live cells were counted by Tissue Blue staining, and 4×10^6^ cells per sample were resuspended in 100 µl FACS buffer. CD16/32 antibody was added and incubated at 4 °C for 30 min to block the Fc sites on the cell surface. Fluorescent antibodies were added in the samples of according to immune cell types and incubate for 30 min on ice. Macrophages were detected by the following surface markers F4/80, CD11b, CD80, MHCII, CD206, DC cells were detected by CD11c and MHCII). NK cells were detected with NKP46., T cells were detected by CD3, CD4, CD8. Detection and analysis of single‐stained tubes and sample tubes by spectral flow analyzer (Cytek, USA).

### RT‐PCR

Total RNA was extracted from tissues or cells using Invitrogen TRIzol reagent (Thermo Fisher Scientific, Waltham, MA, USA), and the purity and integrity of total RNA were tested by observing the ratio of the 28S and 18S bands by agarose gel electrophoresis. Approximately 2 µg total RNA was reverse transcribed to obtain the complementary DNA. Reverse transcription PCR (RT‐PCR) was performed using SYBR Green RT‐PCR Master Mix (BioRad, Hercules, CA, USA) on a CFX 96 RT‐PCR system with specific primers (Table S4, Supporting Information). The RT‐PCR conditions were as follows: 95 °C for 10 min, followed by 40 cycles of 95 °C for 15 s and 60 °C for 1 min. The levels of the target genes were normalized using the 2^−ΔΔCt^ method with β‐actin as reference gene. Each experiment was repeated three times.

### Cell ELISA

To analyze the binding activity of Nb289 with gastric, colon and pancreatic cancer cell lines, cell ELISA was performed as follows. Briefly, gastric cancer cell lines (MKN45 and IM95), pancreatic cancer ASPC1 cell line and colon cancer Colon26 cell line were cultured overnight in 96‐well plates at a density of 5×10^4^/well, and MC38 colon cancer cells with low CDH17 expression were used as a negative cell control. Cells were fixed with 4% paraformaldehyde for 5 min and then incubated with 4% donkey serum solution at RT for 1 h. Nanobodies were diluted from 4000 to 62.5 nM with 1× phosphate‐buffered saline with Tween 20 (0.1%) (PBST) and incubated for 1 h at RT. The plates were then washed three times with PBST, and mouse anti‐HA antibody (Creative Biomart, USA) was added and incubated at RT for 1 h. Next, the plates were washed and incubated with Alexa Fluro 488 linked donkey anti‐mouse IgG antibody (Invitrogen, USA) for 1 h at RT. The plates were washed three times with PBST, and the fluorescence intensity was measured with a Sapphire Capture System (Sapphire, USA).

### Nanobody Labeling with Cy5 Dye

The Cy5 dye labeled nanobody was used for in vivo imaging. Briefly, Nb289 or Control nanobody was diluted to 1 mg mL^−1^ in PBS (pH 6.5). Cy5‐maleimide (Cy5‐mal) was added and the solution was incubated for 2 h at RT in the dark. Unconjugated dye was removed using a 10 K molecular‐weight cutoff (MWCO) spin desalting column. Concentrations of the nanobody were determined using a NanoDrop One spectrophotometer (Thermo Scientific, USA). The final labeling products were analyzed by SDS‐PAGE Komas Brilliant Blue staining and fluorescence intensity was measured with a Sapphire Capture System (Sapphire, USA).

### Engineered Bacterial Labeling with Cy5 or CR

The Cy5 dye labeled engineered bacterial was used for in vivo imaging and the CR labeled engineered bacterial was utilized for photothermal therapy. Briefly, Nb289 or Control nanobody displayed engineered bacterial was diluted to 10^9^ colony forming units (CFU)/mL in PBS (pH 6.5). 100 µg Cy5‐NHS dye or 200 µg CR‐NHS were added, and the solution was incubated for 30 min at RT in the dark. Unconjugated dye was removed by centrifugation. The final labeling products were analyzed by fluorescence microscopy and flow cytometry.

### Macrophage Treatment with Bacterial Lysate

To investigate whether the STING pathway was activated by DNA in the supernatant released by bacteria, the supernatant was treated with DNase I and proteinase K as follows. One ml of 10^7^ bacteria was placed in a 45 °C water bath for 10 min and centrifuged at 12 000 g for 5 min to separate the supernatant and precipitate. 2 U ml^−1^ of DNase I or 0.05 mg ml^−1^ proteinase K was added to the supernatant and incubated at 37 °C for 30 min. 5 µl EDTA (1 mM) was added to the supernatant and incubated at 95 °C for 10 min to inactivate the DNase I or proteinase K. The treated supernatant was added to macrophages and the changes in the expression levels of key proteins of STING pathway were detected by western blot.

### Statistical Analysis

All the data were present as mean ± standard error of the mean (SEM) unless otherwise specified. GraphPad Prism software was used for all statistical analysis. An unpaired two‐tailed student's t‐test was utilized to analyze the difference between two samples. Tumor weight and various toxicological parameters among four groups were analyzed with one‐way ANOVA. Two‐way ANOVA was used to analyze in vitro cell viability and tumor growth curves. Survival curves between groups were compared with a log‐rank test. A p‐value less than 0.05 was considered statistically significant. Asterisks indicate the significant difference (*p < 0.05, **p < 0.01, ***p < 0.001, ****p < 0.0001).

## Conflict of Interest

The authors declare no conflict of interest.

## Supporting information

Supporting Information

## Data Availability

The data that support the findings of this study are available in the supplementary material of this article.

## References

[1] a) E. F. McCarthy , Iowa Orthop. J. 2006, 26, 154; 16789469 PMC1888599

[2] a) S. Chowdhury , S. Castro , C. Coker , T. E. Hinchliffe , N. Arpaia , T. Danino , Nat. Med. 2019, 25, 1057; 31270504 10.1038/s41591-019-0498-z PMC6688650

[3] S. Zhou , C. Gravekamp , D. Bermudes , K. Liu , Nat. Rev. Cancer 2018, 18, 727. 30405213 10.1038/s41568-018-0070-z PMC6902869

[4] X. Lou , Z. Chen , Z. He , M. Sun , J. Sun , Nanomicro. Lett. 2021, 13, 37. 34138211 10.1007/s40820-020-00560-9 PMC8187705

[5] X. Huang , J. Pan , F. Xu , B. Shao , Y. Wang , X. Guo , S. Zhou , Adv. Sci. (Weinh) 2021, 8, 2003572. 33854892 10.1002/advs.202003572 PMC8025040

[6] a) D. S. Leventhal , A. Sokolovska , N. Li , C. Plescia , S. A. Kolodziej , C. W. Gallant , R. Christmas , J. R. Gao , M. J. James , A. Abin‐Fuentes , M. Momin , C. Bergeron , A. Fisher , P. F. Miller , K. A. West , J. M. Lora , Nat. Commun. 2020, 11, 2739; 32483165 10.1038/s41467-020-16602-0 PMC7264239

[7] a) S. H. Park , J. H. Zheng , V. H. Nguyen , S. N. Jiang , D. Y. Kim , M. Szardenings , J. H. Min , Y. Hong , H. E. Choy , J. J. Min , Theranostics 2016, 6, 1672; 27446500 10.7150/thno.16135 PMC4955065

[8] V. Raman , N. Van Dessel , C. L. Hall , V. E. Wetherby , S. A. Whitney , E. L. Kolewe , S. M. K. Bloom , A. Sharma , J. A. Hardy , M. Bollen , A. Van Eynde , N. S. Forbes , Nat. Commun. 2021, 12, 6116. 34675204 10.1038/s41467-021-26367-9 PMC8531320

[9] a) W. Wu , Y. Pu , S. Gao , Y. Shen , M. Zhou , H. Yao , J. Shi , Nanomicro. Lett. 2022, 14, 220; 36367591 10.1007/s40820-022-00951-0 PMC9652197

[10] Y. E. Chen , D. Bousbaine , A. Veinbachs , K. Atabakhsh , A. Dimas , V. K. Yu , A. Zhao , N. J. Enright , K. Nagashima , Y. Belkaid , M. A. Fischbach , Science 2023, 380, 203. 37053311 10.1126/science.abp9563 PMC12356174

[11] a) N. G. Ordonez , Adv. Anat. Pathol. 2014, 21, 131; 24508695 10.1097/PAP.0000000000000008

[12] a) K. Fujiwara , H. Akiba , A. B. Tsuji , H. Sudo , A. Sugyo , K. Nagatsu , M. R. Zhang , H. Iwanari , O. Kusano‐Arai , S. Kudo , C. Kikuchi , K. Tsumoto , T. Momose , T. Hamakubo , T. Higashi , Nucl. Med. Commun. 2020, 41, 688; 32371673 10.1097/MNM.0000000000001203

[13] F. Jacobsen , R. Pushpadevan , F. Viehweger , M. Freytag , R. Schlichter , N. Gorbokon , F. Buscheck , A. M. Luebke , D. Putri , M. Kluth , C. Hube‐Magg , A. Hinsch , D. Hoflmayer , C. Fraune , C. Bernreuther , P. Lebok , G. Sauter , S. Minner , S. Steurer , R. Simon , E. Burandt , D. Dum , F. Lutz , A. H. Marx , T. Krech , T. S. Clauditz , Pathol. Res. Pract. 2024, 256, 155175. 38452580 10.1016/j.prp.2024.155175

[14] W. Tian , J. Zhao , W. Wang , LUNG 2023, 201, 489. 37823901 10.1007/s00408-023-00648-0

[15] T. J. Harmand , A. Islam , N. Pishesha , H. L. Ploegh , RSC Chem. Biol. 2021, 2, 685. 34212147 10.1039/d1cb00023c PMC8190910

[16] a) S. N. Jiang , T. X. Phan , T. K. Nam , V. H. Nguyen , H. S. Kim , H. S. Bom , H. E. Choy , Y. Hong , J. J. Min , Mol. Ther. 2010, 18, 635; 20051939 10.1038/mt.2009.295 PMC2839435

[17] V. Salema , E. Marin , R. Martinez‐Arteaga , D. Ruano‐Gallego , S. Fraile , Y. Margolles , X. Teira , C. Gutierrez , G. Bodelon , L. A. Fernandez , PLoS One 2013, 8, e75126. 24086454 10.1371/journal.pone.0075126 PMC3781032

[18] a) E. Csibra , M. Renders , V. B. Pinheiro , ChemBioChem 2020, 21, 2844; 32413179 10.1002/cbic.202000203 PMC7586821

[19] P. Xia , H. Yuan , M. Tian , T. Zhong , R. Hou , X. Xu , J. Ma , H. Wang , Z. Li , D. Huang , C. Qu , L. Dai , C. Xu , C. Yang , H. Jiang , Y. He , F. Rückert , Z. Li , Y. Yuan , J. Wang , Adv. Funct. Mater. 2022, 33, 1793.

[20] a) L. D. Wood , M. I. Canto , E. M. Jaffee , D. M. Simeone , Gastroenterology 2022, 163, 386; 35398344 10.1053/j.gastro.2022.03.056 PMC9516440

[21] L. H. Truong , S. Pauklin , Cancers 2021, 13, 5028. 34638513 10.3390/cancers13195028 PMC8507722

[22] a) T. Shang , X. Yu , S. Han , B. Yang , Biomater. Sci. 2020, 8, 5241; 32996922 10.1039/d0bm01158d

[23] X. Hou , Y. Tao , Y. Pang , X. Li , G. Jiang , Y. Liu , Int. J. Cancer 2018, 143, 3050. 29981170 10.1002/ijc.31717

[24] X. Gao , S. Jiang , C. Li , Y. Chen , Y. Zhang , P. Huang , J. Lin , Biomaterials 2021, 267, 120454. 33160122 10.1016/j.biomaterials.2020.120454

[25] A. Shahrivarkevishahi , M. A. Luzuriaga , F. C. Herbert , A. C. Tumac , O. R. Brohlin , Y. H. Wijesundara , A. V. Adlooru , C. Benjamin , H. Lee , P. Parsamian , J. Gadhvi , N. J. De Nisco , J. J. Gassensmith , J. Am. Chem. Soc. 2021, 143, 16428. 34551259 10.1021/jacs.1c05090

[26] a) R. Gupta , I. Amanam , V. Chung , J. Surg. Oncol. 2017, 116, 25; 28591939 10.1002/jso.24623

[27] a) T. Yin , Z. Diao , N. T. Blum , L. Qiu , A. Ma , P. Huang , Small 2022, 18, e2104643; 34908239 10.1002/smll.202104643

[28] L. Huang , Y. Li , Y. Du , Y. Zhang , X. Wang , Y. Ding , X. Yang , F. Meng , J. Tu , L. Luo , C. Sun , Nat. Commun. 2019, 10, 4871. 31653838 10.1038/s41467-019-12771-9 PMC6814770

[29] a) S. Akira , S. Uematsu , O. Takeuchi , Cell 2006, 124, 783; 16497588 10.1016/j.cell.2006.02.015

[30] T. Reislander , F. J. Groelly , M. Tarsounas , Mol. Cell 2020, 80, 21. 32810436 10.1016/j.molcel.2020.07.026

[31] M. Motwani , S. Pesiridis , K. A. Fitzgerald , Nat. Rev. Genet. 2019, 20, 657. 31358977 10.1038/s41576-019-0151-1

[32] A. de Mingo Pulido , K. Hanggi , D. P. Celias , A. Gardner , J. Li , B. Batista‐Bittencourt , E. Mohamed , J. Trillo‐Tinoco , O. Osunmakinde , R. Pena , A. Onimus , T. Kaisho , J. Kaufmann , K. McEachern , H. Soliman , V. C. Luca , P. C. Rodriguez , X. Yu , B. Ruffell , Immunity 2021, 54, 1154. 33979578 10.1016/j.immuni.2021.04.019 PMC8192496

[33] A. Kosaka , K. Ishibashi , T. Nagato , H. Kitamura , Y. Fujiwara , S. Yasuda , M. Nagata , S. Harabuchi , R. Hayashi , Y. Yajima , K. Ohara , T. Kumai , N. Aoki , Y. Komohara , K. Oikawa , Y. Harabuchi , M. Kitada , H. Kobayashi , T. Ohkuri , J. Exp. Med. 2021, 218. 10.1084/jem.20200792 PMC8480673 34559187

[34] J. T. Sockolosky , M. Dougan , J. R. Ingram , C. C. Ho , M. J. Kauke , S. C. Almo , H. L. Ploegh , K. C. Garcia , Proc. Natl. Acad. Sci. U S A 2016, 113, E2646. 27091975 10.1073/pnas.1604268113 PMC4868409

[35] a) A. C. Bloom , L. H. Bender , S. Tiwary , L. Pasquet , K. Clark , T. Jiang , Z. Xia , A. Morales‐Kastresana , J. C. Jones , I. Walters , M. Terabe , J. A. Berzofsky , OncoImmunology 2019, 8, e1625687; 31646070 10.1080/2162402X.2019.1625687 PMC6791426

[36] a) K. Fujiwara , A. B. Tsuji , H. Sudo , A. Sugyo , H. Akiba , H. Iwanari , O. Kusano‐Arai , K. Tsumoto , T. Momose , T. Hamakubo , T. Higashi , Ann. Nucl. Med. 2020, 34, 13; 31605356 10.1007/s12149-019-01408-y PMC6970965

[37] S. Muyldermans , Annu. Rev. Biochem. 2013, 82, 775. 23495938 10.1146/annurev-biochem-063011-092449

[38] S. Sun , Z. Ding , X. Yang , X. Zhao , M. Zhao , L. Gao , Q. Chen , S. Xie , A. Liu , S. Yin , Z. Xu , X. Lu , Int. J. Nanomedicine 2021, 16, 2337. 33790553 10.2147/IJN.S297631 PMC7997558

[39] M. le Besnerais , A. Veyradier , Y. Benhamou , P. Coppo , Expert Opin. Biol. Ther. 2019, 19, 1127. 31359806 10.1080/14712598.2019.1650908

[40] a) S. De Munter , J. Ingels , G. Goetgeluk , S. Bonte , M. Pille , K. Weening , T. Kerre , H. Abken , B. Vandekerckhove , Int. J. Mol. Sci. 2018, 19, 403; 29385713 10.3390/ijms19020403 PMC5855625

[41] M. Feng , H. Bian , X. Wu , T. Fu , Y. Fu , J. Hong , B. D. Fleming , M. F. Flajnik , M. Ho , Antib. Ther. 2019, 2, 1. 10.1093/abt/tby011 PMC6312525 30627698

[42] a) M. J. Bender , A. C. McPherson , C. M. Phelps , S. P. Pandey , C. R. Laughlin , J. H. Shapira , L. M. Sanchez , M. Rana , T. G. Richie , T. S. Mims , A. M. Gocher‐Demske , L. Cervantes‐Barragan , S. J. Mullett , S. L. Gelhaus , T. C. Bruno , N. Cannon , J. A. McCulloch , D. A. A. Vignali , R. Hinterleitner , A. V. Joglekar , J. F. Pierre , S. T. M. Lee , D. Davar , H. M. Zarour , M. Meisel , Cell 2023, 186, 1846; 37028428 10.1016/j.cell.2023.03.011 PMC10148916

[43] S. Xie , L. Zhao , X. Song , M. Tang , C. Mo , X. Li , J. Control Release 2017, 268, 390. 29101053 10.1016/j.jconrel.2017.10.041

[44] W. Zeng , Z. Li , H. Chen , X. Zeng , L. Mei , Cell Reports Phys. Sci. 2022, 3, 100663.

[45] B. S. Pan , S. A. Perera , J. A. Piesvaux , J. P. Presland , G. K. Schroeder , J. N. Cumming , B. W. Trotter , M. D. Altman , A. V. Buevich , B. Cash , S. Cemerski , W. Chang , Y. Chen , P. J. Dandliker , G. Feng , A. Haidle , T. Henderson , J. Jewell , I. Kariv , I. Knemeyer , J. Kopinja , B. M. Lacey , J. Laskey , C. A. Lesburg , R. Liang , B. J. Long , M. Lu , Y. Ma , E. C. Minnihan , G. O'Donnell , et al., Science 2020, 369, eaba6098.32820094

[46] J. Ahn , T. Xia , A. Rabasa Capote , D. Betancourt , G. N. Barber , Cancer Cell 2018, 33, 862. 29706455 10.1016/j.ccell.2018.03.027 PMC6177226

