# Supplementary material for: Nanobody‐Engineered Biohybrid Bacteria Targeting Gastrointestinal Cancers Induce Robust STING‐Mediated Anti‐Tumor Immunity
Source: Adv Sci (Weinh). 2024 Jun 18;11(31):2401905. doi: 10.1002/advs.202401905 (PMC11336900; doi:10.1002/advs.202401905)
Supplement: Supplementary file 1 — Supporting Information [file ADVS-11-2401905-s001.pdf]

ADVANCED SCIENCE
Open Access

Supporting Information

for *Adv. Sci.*, DOI 10.1002/advs.202401905

Nanobody-Engineered Biohybrid Bacteria Targeting Gastrointestinal Cancers Induce Robust STING-Mediated Anti-Tumor Immunity

Xiaolong Xu, Youbin Ding, Yafang Dong, Haitao Yuan, Peng Xia, Chengming Qu, Jingbo Ma, Huifang Wang, Xiaodong Zhang, Liang Zhao, Zhijie Li, Zhen Liang* and Jigang Wang**

Nanobody-engineered biohybrid bacteria targeting gastrointestinal cancers induce robust STING-mediated anti-tumor immunity

Xiaolong Xu[1, 2 #], Youbin Ding[3, #], Yafang Dong[1], Haitao Yuan[1], Peng Xia[4], Chengming Qu[4], Jingbo Ma[1], Huifang Wang[1], Xiaodong Zhang[3], Liang Zhao[5, 6], Zhijie Li[1, *] Zhen Liang[1, *], Jigang Wang[1, 7, 8, 9, 10, *]

[1]Department of Geriatrics and Shenzhen Clinical Research Centre for Geriatrics, Shenzhen People's Hospital (The First Affiliated Hospital, Southern University of Science and Technology; The Second Clinical Medical College, Jinan University,), Shenzhen, Guangdong 518020, China

[2]Integrated Chinese and Western Medicine Postdoctoral Research Station, Jinan University, Guangzhou 510632, China.

[3]Department of Medical Imaging, The Third Affiliated Hospital, Southern Medical University (Academy of Orthopedics Guangdong Province), Guangzhou, 510515, China

[4]Department of Hepatobiliary & Pancreatic Surgery, Zhongnan Hospital of Wuhan University, Wuhan, Hubei 430071, China

[5]Department of Pathology, Shunde Hospital, Southern Medical University (The First People's Hospital of Shunde), Foshan, 528308, China

[6]Department of Pathology & Guangdong Province Key Laboratory of Molecular Tumor Pathology, School of Basic Medical Sciences, Southern Medical University, Guangzhou, 510515, China

[7]Department of Oncology, The Affiliated Hospital of Southwest Medical University, Luzhou, Sichuan, 646000, China.

[8]Department of Traditional Chinese Medicine and School of Pharmaceutical Sciences, Southern Medical University, Guangzhou, 510515, China.

[9]State Key Laboratory for Quality Ensurance and Sustainable Use of Dao-di Herbs, Artemisinin Research Center, and Institute of Chinese Materia Medica, China Academy of Chinese Medical Sciences, Beijing, 100700, China.

[10]State Key Laboratory of Antiviral Drugs, School of Pharmacy, Henan University, Kaifeng, 475004, China.

[#]These authors equally contribute to this work.

*Corresponding authors:

Jigang Wang, email: wangjigang@u.nus.edu

Zhen Liang, email: Liang.zhen@szhospital.com

Zhijie Li, email: li.zhijie@szhospital.com

Supporting information

Method

Synthesis of Croconium Dye: Croconium dye was synthesized as described previously [1].1 The synthetic route was shown in **Figure S17**. All chemicals were commercially available and used as received without further purification unless otherwise specified. Reaction progress was monitored by thin-layer chromatography (TLC) on silica plates (250 µmol/L thickness, bought from Qingdao Haiyang Chemical Co.) and spots were visualized by UV254 and 365 fluorescent indicators. Flash column chromatography was carried out using silica gel (100 mesh) bought from Aladdin. The 1H NMR and 13C NMR spectra were collected on a Bruker ARX 400 and 600 MHz spectrometer.

Peritoneal macrophage extraction: Mouse peritoneal macrophage induction and extraction were performed as reported previously [2]. 4% Thioglycolate solution was prepared and deionized. 6–8-week-old BALB/c mice were used and injected intraperitoneally with 1 mL of the prepared 4% Thioglycolate solution. After 48 hours, mice were sacrificed and the peritoneal cavity was lavaged with 5 ml of DMEM medium. The collected media were centrifugated at 400 × g for 5 min to pellet the cells in the lavage solution. The cells were resuspended in DMEM containing 10% fetal bovine serum. The cells were then counted and adjusted to a concentration of 3 million cells/mL and added to culture dishes at 37 °C in an incubator with 5% CO2. After 48 h, macrophages were confirmed with flow cytometry with F4/80 and CD11b antibodies. The subsequent analysis was conducted with the isolated macrophages.

Immunohistochemical staining: To analyze and evaluate the expression level of CDH17 in cancer tissues, CDH17 was stained in 3 tissue microarrays (TMA). These TMA chips including gastric cancer (93 cases), pancreatic cancer (129 cases) and colorectal cancer (116 cases) with clinical information were purchased from company (OUTDO BIOTECH, Shanghai, China). The sections were stained with a rabbit polyclonal antibody against CDH17 (Abclonal, USA). CDH17 detection was performed by biotin-conjugated goat anti-rabbit IgG secondary antibody and ABC kit (Vector, USA) followed by colorimetric detection using diaminobenzidine (DAB; Vector, USA). The images were obtained using an inverted light microscope (Leica, Wetzlar, Germany). The procedure involving human samples was approved by the medical ethical committee of the Shenzhen People's Hospital and OUTDO BIOTECH. The clinical information is described in Table S1-S3. Other staining: IBA, P-IRF3, P65

Induction and characterization of nanobodies engineered onto bacterial: To make the nanobody fusion protein efficiently expressed on the outer membrane of engineered bacteria, the conditions of protein expression induction were optimized as follows. The nanobody fusion protein expression plasmid was transformed into MG1655 bacteria, and then the single clone was picked into 5 ml of LB medium containing 50 μg/ml chloramphenicol and incubated at 37 ℃ and 200 rpm until the OD600 ≈ 0.6.

Then the culture medium containing the bacteria was ice-bathed for 30 min, and then 0.5 mM IPTG was added to induce protein expression with the incubation at 15 ℃ and 200 rpm for 16 h. 1mL of bacteria was taken and stained with HA-Tag Mouse mAb (Alexa Fluor® 488 Conjugate) antibody, and the percentage of positive bacteria was analyzed by flow cytometry.

Quantitative analysis of nanobodies expressed by engineered bacteria: To determine the average number of nanobodies expressed on each engineered bacterium, Fluorescent western blot (FWB) was first used to establish a standard curve with known nanobody (Nb289) quantity (1000 ng, 500 ng, 250 ng, 125 ng, 62.5 ng) and the corresponding fluorescence intensity, and Rabbit Anti-VHH Antibody was used as a primary antibody and IR680-coupled sheep anti-rabbit antibody as a secondary antibody in the FWB analysis. The bacterial number was quantified with OD600 absorbance. Based on the standard curve, the copy numbers of nanobodies in engineered bacteria were calculated through the analysis of fluorescence density for the known number of engineered bacteria in FWB.

Affinity Measurement: To determine the binding properties of Nb289 and Nb535 nanobody to human and murine CDH17, the SPRanalysis was performed on Biacore T200 (GE Healthcare) according to the manufacturer's instructions. The CM5 sensor chip (GE Healthcare) was used for capturing purified 1-3 domain of CDH17 protein at a concentration of about 10 μg/mL and a flow rate of 10 μl/min for 1 min. This resulted in coupling of ~300 RU CDH17 D1-3 to the sensor chip surface. Meanwhile, one flow cell of the sensor chip was left without nanobody captured to be used a reference control. Nanobodies were prepared at different concentrations starting from 2000 nM to 7.81 nM with a 2-fold serial dilution in running buffer (1×PBS, 0.005% Tween-20, pH 7.4).
For each Nb construct, sensorgrams were recorded for different analyte concentrations at a flow rate of 30 μl/min and a data collection rate of 1 Hz. Analyte injections were performed with association and dissociation phases of 180 s and 300 s, respectively. Prior to data analysis, reference and zero concentration data were subtracted from the sensorgrams. The collected data were fitted according to a 1:1 Langmuir binding model (one Nb to one monomer of CDH17 D1-3) from the experimental flow cells of a single biosensor chip. All experimental data were treated with Biacore T200 Evaluation Software to calculate the kinetic k_{on} and k_{off} rates, and the corresponding equilibrium dissociation constant (K_D).

Detection of IFN-β in Blood and Tumor Tissue: Enzyme-linked immunosorbent assays of IFNβ detection in bood and tumor tissue were performed following previous study[3]. For the detection of IFNγ in blood, we took 300 microliters of anticoagulated blood added to 700 μl of 0.9% NaCl solution and centrifuged horizontally with a centrifugal force of 300 g for 10 minutes at 4 °C. 100 μl of supernatant were used for the detection of IFNβ by the IFNβ assay kit (Beyotime Biotechnology, China).To measure the levels of IFNβ in tumor tissues, approximately 200 mg of tumor tissue

was placed in 500 μl of lysis buffer[4] containing protease inhibitors (Beyotime Biotechnology, China) and homogenized on ice for 60 seconds. Samples were centrifuged (2000× g) at 4 °C for 5 minutes, and supernatant was used for IFNβ and tested for total protein by BCA Protein Assay Kits (Thermo, USA). IFN-γ concentration in tumor tissue was normalized to total protein.

STING pathway blocking assay with inhibitor H151: To analyze whether the tumor-suppressing effect of engineered bacteria-mediated PTT plus CD47 nanobody was dependent on the activation of the STING pathway, an animal experiment with the STING inhibitor was conducted as follows. H151 (10 mg/kg body weight, daily IP) was first used to pre-treat the mice with Colon26 tumors for two days and then cotreated the mice with engineered bacteria-mediated PPT plus CD47 nanobody (5×10^6 CFU and irradiation plus two injections of CD47 nanobody, 200 μg/mouse) as illustrated in Figure S33C. Mouse body weight and tumor volume were monitored daily during this treatment period. Statistical analysis was performed and the PBS group, and the group without H151 treatment as controls (n=6 per group).

Reference

[1] A. Shahrivarkevishahi, M. A. Luzuriaga, F. C. Herbert, A. C. Tumac, O. R. Brohlin, Y. H. Wijesundara, A. V. Adlooru, C. Benjamin, H. Lee, P. Parsamian, J. Gadhvi, N. J. De Nisco, J. J. Gassensmith, *J. Am. Chem. Soc.* **2021**, 143, 16428.

[2] A. R. Jang, M. J. Kang, J. I. Shin, S. W. Kwon, J. Y. Park, J. H. Ahn, T. S. Lee, D. Y. Kim, B. G. Choi, M. W. Seo, S. J. Yang, M. K. Shin, J. H. Park, *Front. Immunol.* **2020**, 11, 1121.

[3] S. A. Gerber, A. L. Sedlacek, K. R. Cron, S. P. Murphy, J. G. Frelinger, E. M. Lord, *Am. J. Pathol.* **2013**, 182, 2345.

[4] S. A. Gerber, J. S. Pober, *J. Immunol.* **2008**, 181, 1052.

[5] C. UniProt, *Nucleic Acids Res.* **2023**, 51, D523.

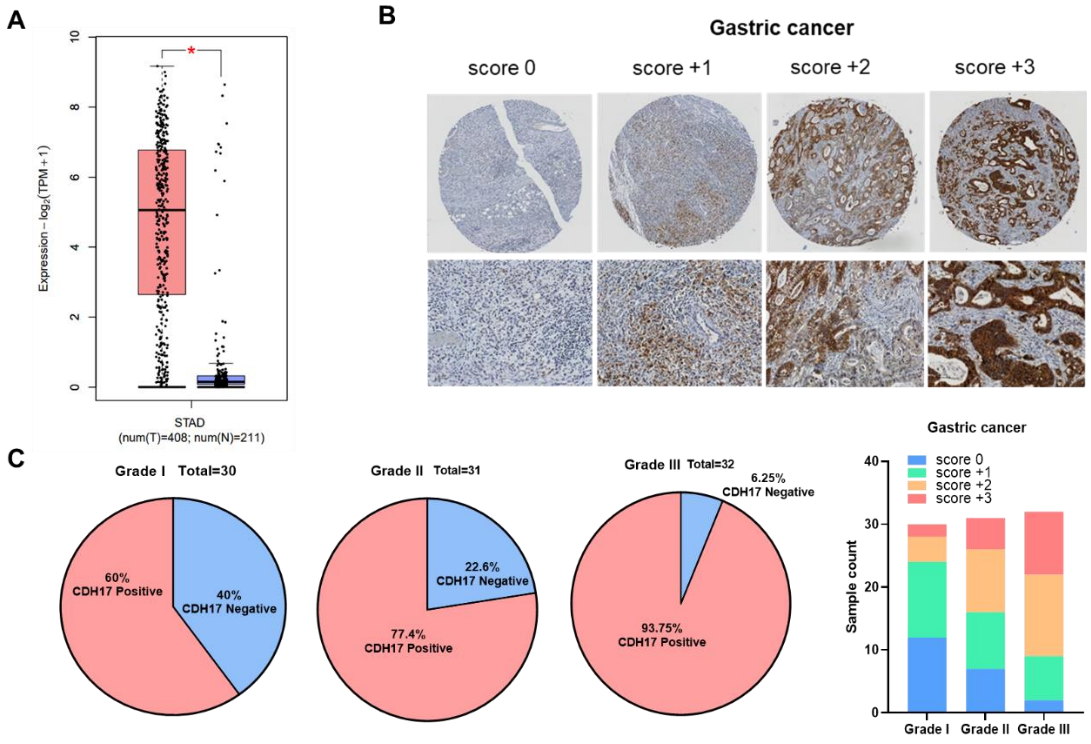

Figure S1. Analysis of the expression of CDH17 in gastric cancer tissues by TCGA database and immunohistochemical staining in a tissue microarray

A, Analysis of mRNA expression of CDH17 in gastric cancer in TCGA database, "*" represents $P<0.05$, tumor tissue n=408, normal tissue n=211. **B,** Immunohistochemical staining analysis of CDH17 expression in a gastric cancer tissue microarray containing 93 cases. Staining scoring in the tissue microarray was examined by Image J according to the degree of DAB staining. **C,** Statistics analysis of the expression of CDH17 combined with pathological grading. Left, the expression of CDH17 in different pathological grades; right, the score percentage of CDH17 expression in different pathological grades (n=93).

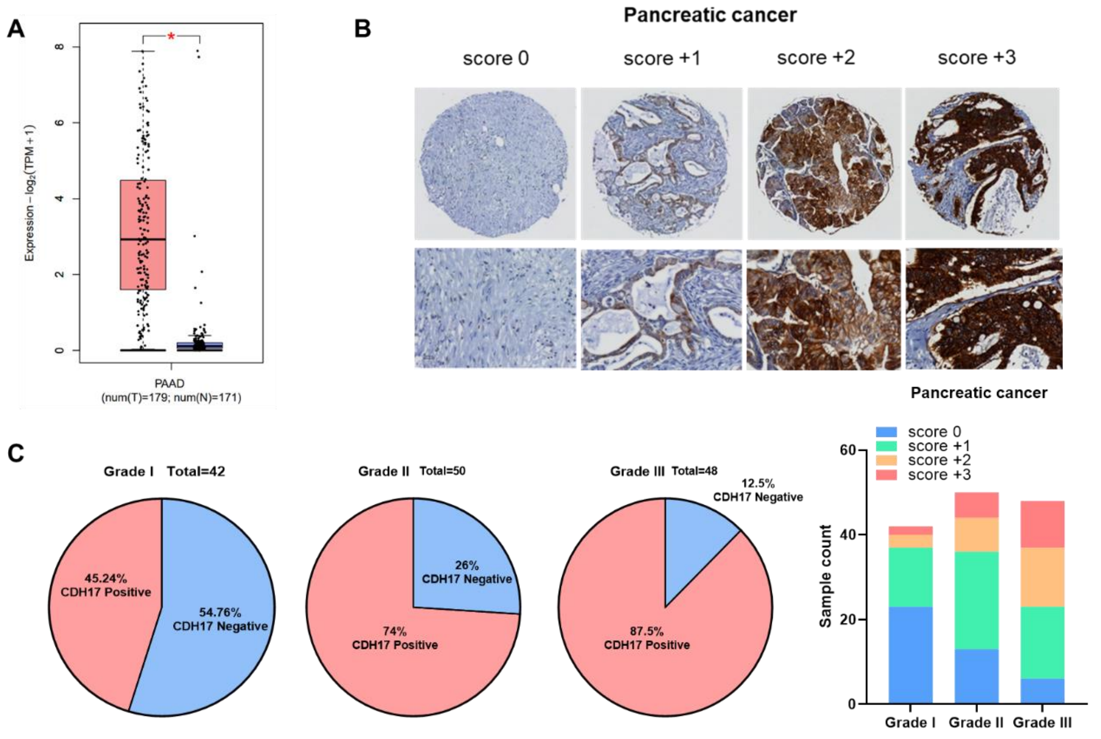

Figure S2. Analysis of the expression of CDH17 in pancreatic cancer tissues by TCGA database and immunohistochemical staining in a tissue microarray

A, Analysis of mRNA expression of CDH17 in pancreatic cancer in TCGA database, "*" represents *P*<0.05, tumor tissue n=179, normal tissue n=171. **C,** Immunohistochemical staining analysis of CDH17 expression in a pancreatic cancer tissue microarray containing 140 cases. Staining scoring in the tissue microarray was examined by Image J according to the degree of DAB staining. **B,** Combined statistics analysis of the expression of CDH17 with pathological grading. Left, the expression of CDH17 in different pathological grades; right, the score percentage of CDH17 expression in different pathological grades (n=140).

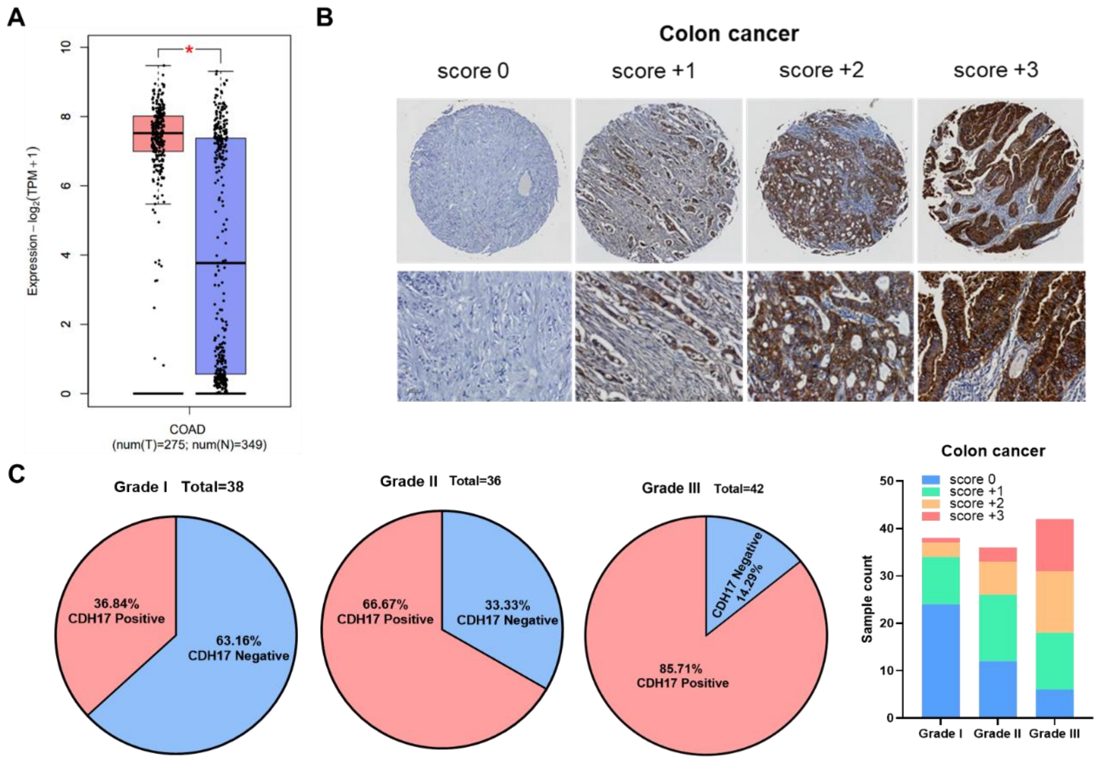

Figure S3. Analysis of the expression of CDH17 in colorectal cancer tissues by TCGA database and immunohistochemical staining in a tissue microarray

A, Analysis of mRNA expression of CDH17 in colon cancer in TCGA database, "*" represents $P<0.05$, tumor tissue n=275, normal tissue n=349. **B,** Immunohistochemical staining analysis of CDH17 expression in a colorectal cancer tissue microarray containing 116 cases. Staining scoring in the tissue microarray was examined by Image J according to the degree of DAB staining. **C,** Combined statistics analysis of the expression of CDH17 with pathological grading. Left, the expression of CDH17 in different pathological grades; right, the score percentage of CDH17 expression in different pathological grades (n=116).

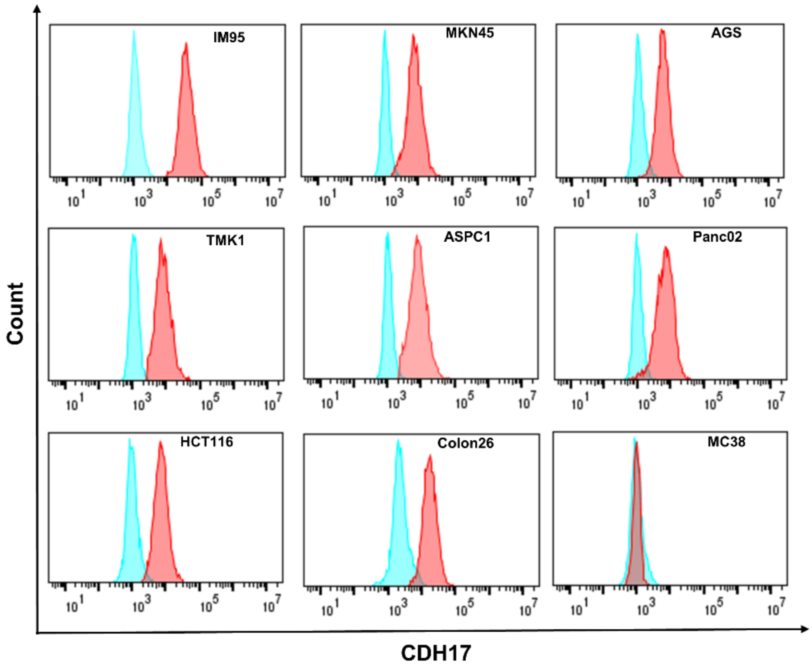

Figure S4. Flow cytometry analysis of CDH17 expression on membranes of cells from gastric, pancreatic and colon cancers

*Blue panel represents control cells, and red panel represents CDH17 antibody-stained cells.1×10^6 cells of each cell lines were harvested with 0.04%EDTA buffer. The CDH17 protein on cell membrane was probed by rabbit anti-CDH17 antibody (Abclonal, USA) with the unstained cells as control. The CHD17 antibody was labeled by Alexa488 conjugated donkey anti-Rabbit (Thermo, USA) antibody and detected by flow cytometry.

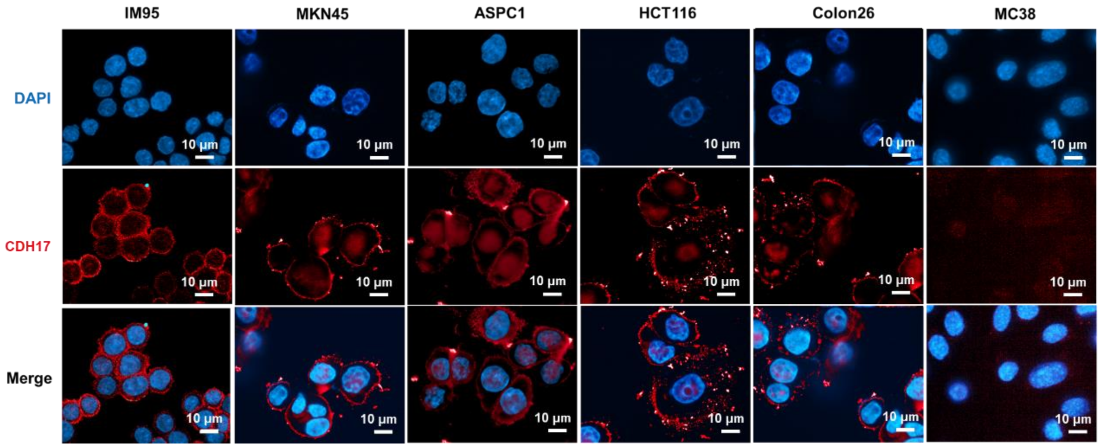

Figure S5. Immunofluorescence analysis of CDH17 expression on the membrane surface in a panel of cancer cells

5×10^3 cells of each cell lines were plated into PhenoPlate 96-well microplates (PerkinElmer, USA). The CDH17 protein on cell membrane was probed by rabbit anti-CDH17 antibody and the CHD17 antibody was labeled by Alexa594 conjugated donkey anti-Rabbit (Thermo, USA) antibody. Fluorescence signal was detected with the High-Content Screening System (PerkinElmer, USA), and CDH17 staining in the MC38 cell line with low CDH17 expression was used as a negative control.

***** Scale bars, 10 µm.

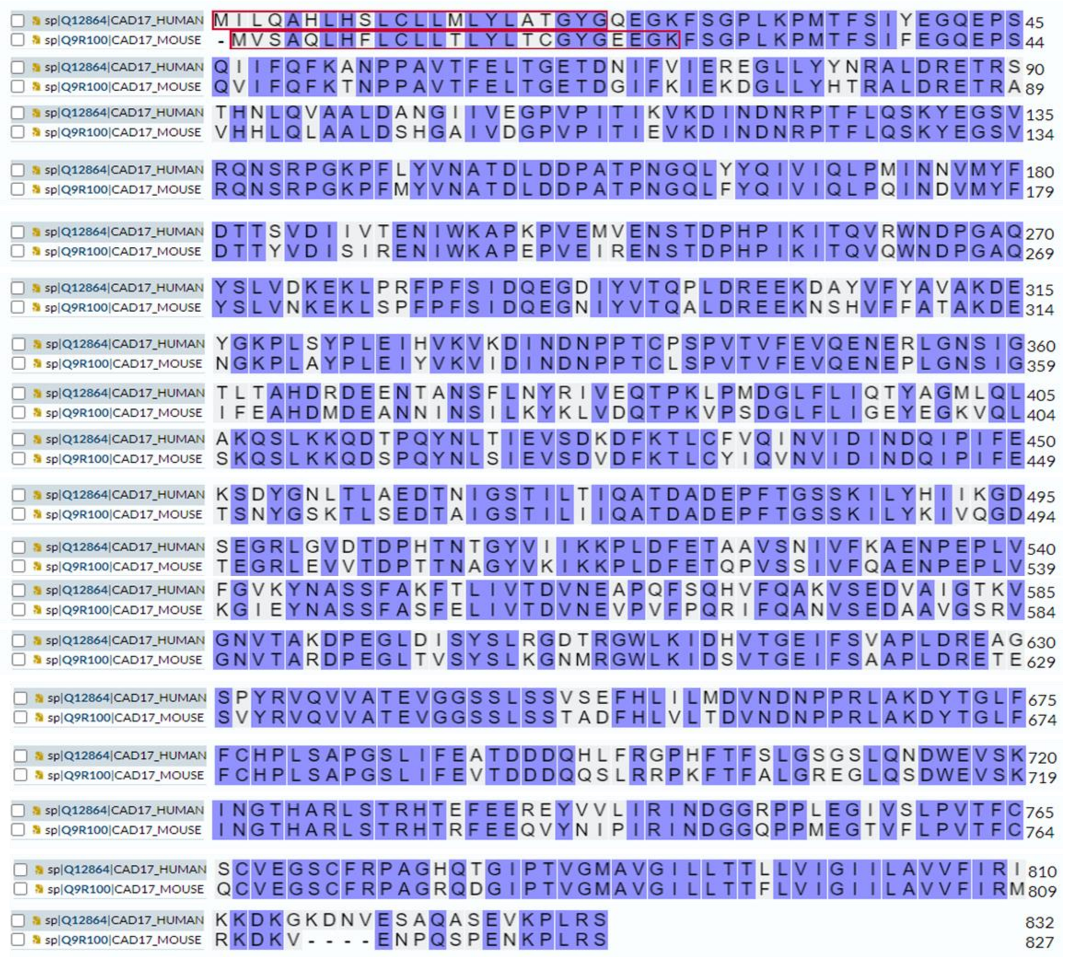

Figure S6. Amino acid sequence alignment analysis of human-derived and mouse-derived CDH17 proteins

The full-length amino acid sequences of CDH17 human and murine proteins were downloaded from the Universal Protein Knowledgebase (UNIPROT)[5] and analyzed by the sequence alignment software provided with the database. The CDH17 protein was highly conserved in human and mouse, with 79% sequence similarity between the two proteins. The blue region in the sequence highlights the exact same amino acids between two proteins.

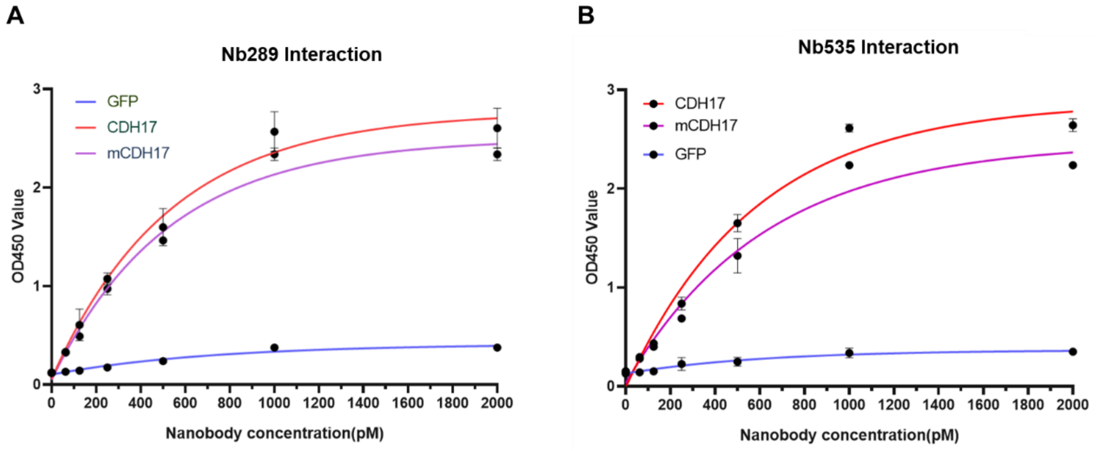

Figure S7. ELISA to detect the binding specificity of Nb535 and Nb289 nanobodies.

A, The binding activity of Nb289 nanobody were detected in the plates respectively coated with human-derived, mouse-derived CDH17 and green fluorescent protein GFP, indicating that Nb289 nanobody could specifically recognize CDH17 proteins.

B, The binding activity of Nb535 nanobody were detected in the plates respectively coated with human-derived, mouse-derived CDH17 and green fluorescent protein GFP, indicating that Nb535 nanobody could specifically recognize CDH17 proteins.

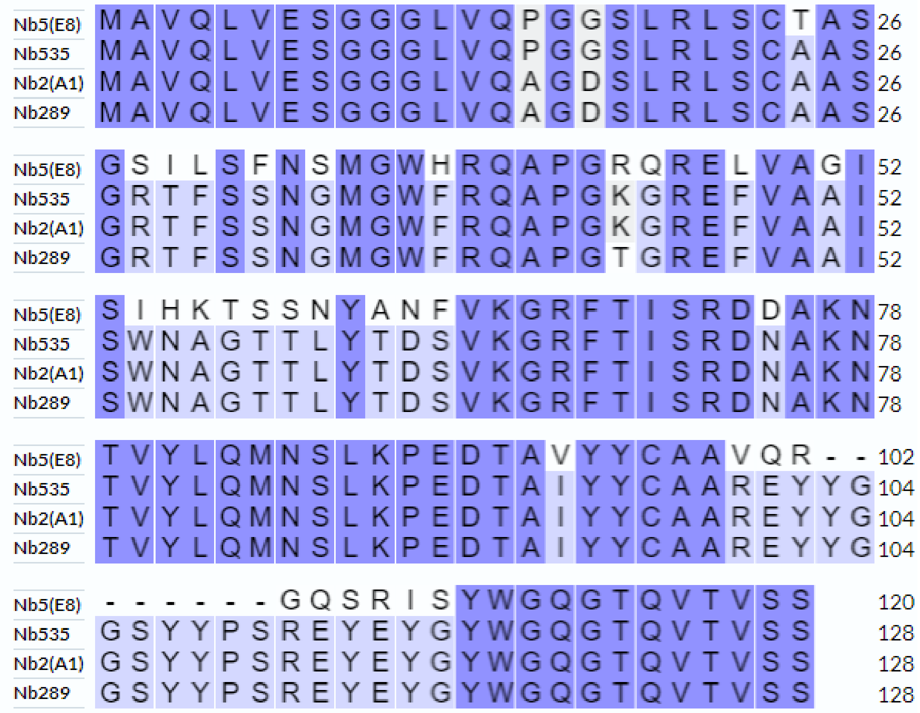

Figure S8. Amino acid sequence alignment analysis of four CDH17 nanobodies.

The amino acid sequences of the nanobodies Nb289, Nb535, Nb2 (A1) and Nb5 (E8) of CDH17 were compared by the comparison software from the UNIPROT database. The comparison results displayed that the sequences of Nb289, Nb535 and A1 were highly homologue with the difference only in a few amino acids. The highlighted region with blue in the sequences was the exact same amino acids among those nanobodies.

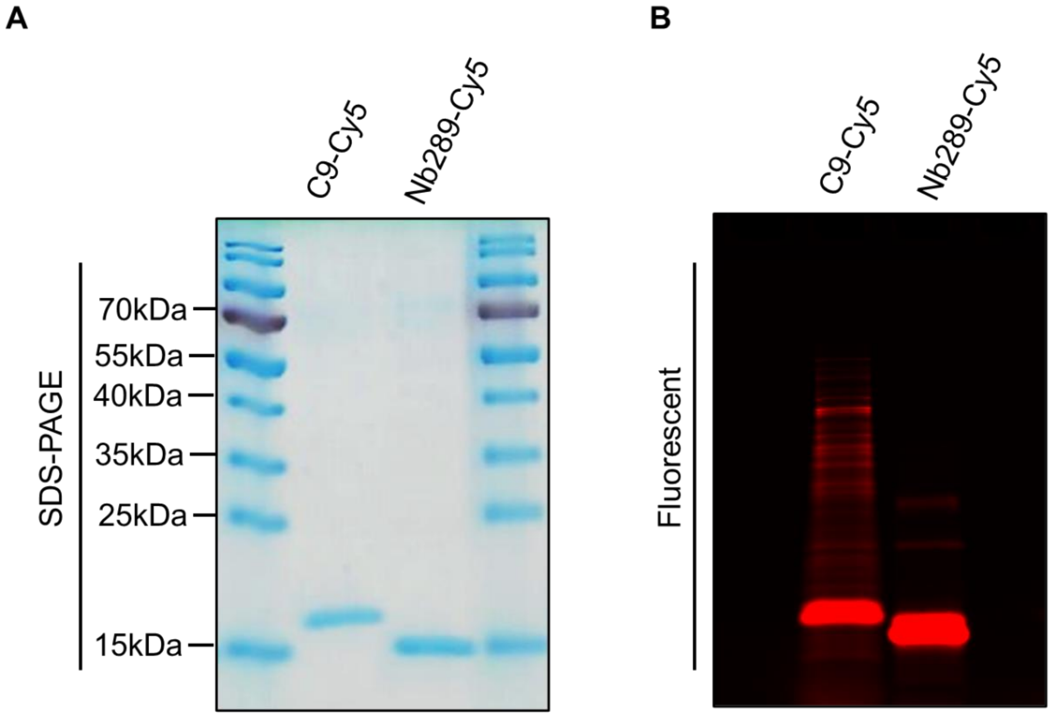

Figure S9. SDS-PAGE electrophoretic analysis of Cy5-labeled nanobodies.
A, Coomassie Brilliant Blue staining of Cy5-labeled nanobodies. **B,** Fluorescence detection of Cy5-labeled nanobodies.

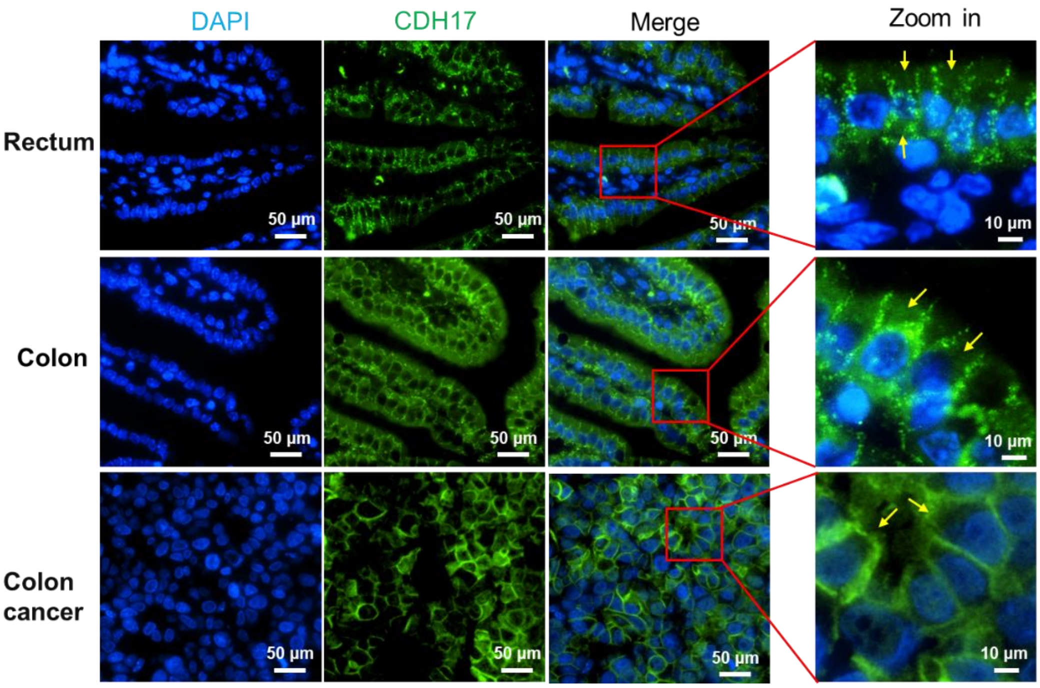

Figure S10. Immunofluorescence analysis of CDH17 expression in normal colorectal and colorectal cancer tissues.

The yellow arrows marked the luminal or basal surface of the tissue. Scale bars，50 μm (left), 10 μm (right).

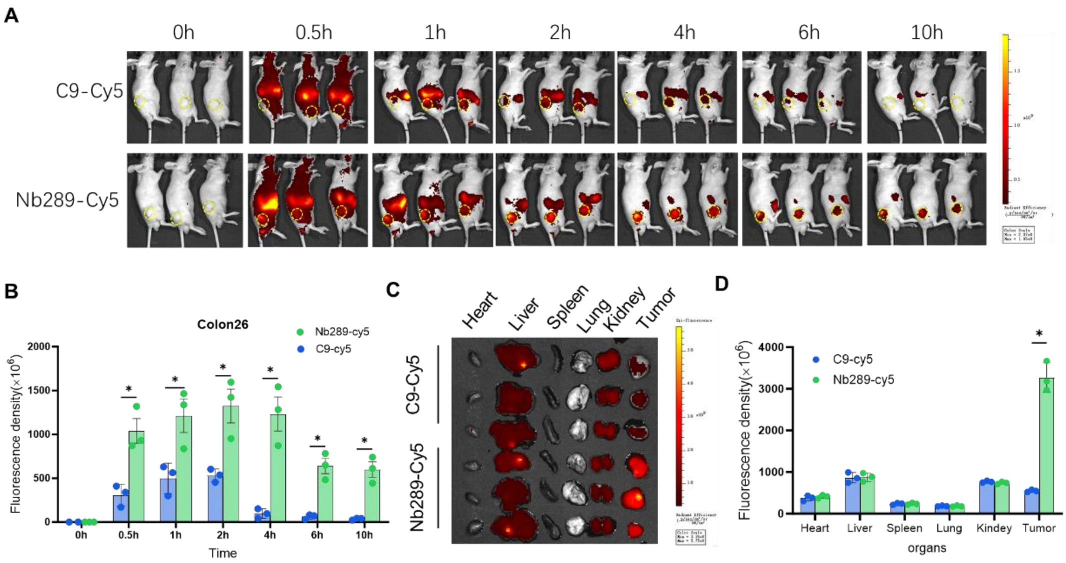

Figure S11. In vivo imaging analysis of Nb289 in MKN45 tumor model with a higher dose. **A**, In vivo imaging analysis of Nb289 in MKN45 tumor model. Nb289 and control Nb C9 were labeled with Cy5, and in vivo imaging was performed at different time points (0.5, 1, 2, 4, 6 and 10 h) after nanobody injection (5 mg/kg, n = 3 per group). **B**, Quantification analysis of fluorescence intensity in tumor tissues. **C**, Ex vivo imaging analysis of the major organs from tumor-bearing mice receiving nanobody injection. The organs were collected from mice for in vivo imaging analysis after 10h circulation (n = 3 per group). **D**, Quantification analysis of fluorescence intensity from various organs. *P<0.05.

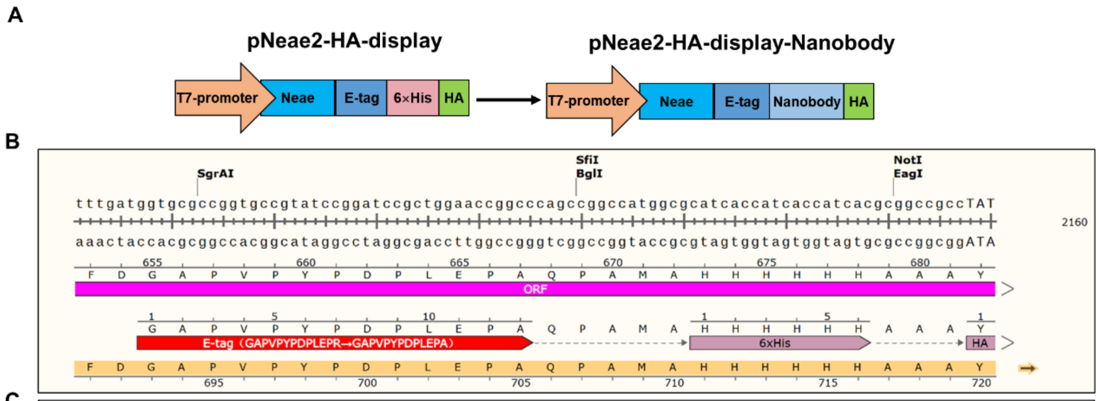

C

pNeae2-HA-display-Nb289
GGCCCAGCCGGCC (SfiI)
MAVQLVESGGGLVQAGDSLRLSCAASGRTFSSNGMGWFRQAPGTGREFVAAISWNAGTTLYTDSVKGRFTISRDNAKNTVYLQMNSLKPEDTAIYYCAARE
YYGGSYYPSREYEYGYWGQGTQVTVSS GCGGCCGC (NotI)
pNeae2-HA-display-Nb535
GGCCCAGCCGGCC (SfiI)
MAVQLVESGGGLVQPGGSLRLSCAASGRTFSSNGMGWFRQAPGKGREFVAAISWNAGTTLYTDSVKGRFTISRDNAKNTVYLQMNSLKPEDTAIYYCAARE
YYGGSYYPSREYEYGYWGQGTQVTVSS GCGGCCGC (NotI)

Figure S12. Construction of nanobody display plasmids.

A, The construction diagram of nanobody display, **B,** The detailed information of enzyme cleavage sites in the pNeae2 display vector ，**C,** The detailed information of Nb289 and Nb535 nanobodies in the display vector.

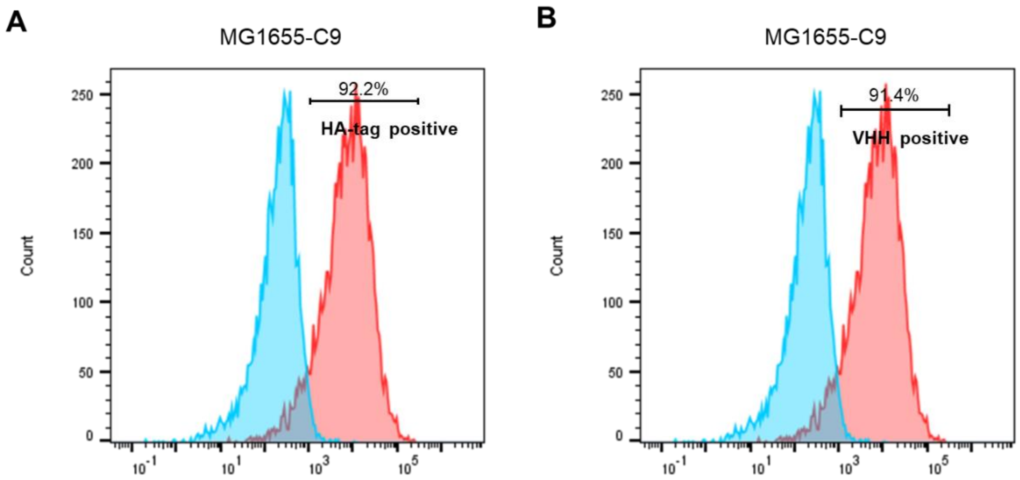

Figure S13. Flow cytometry detection of the display efficiency of control nanobodies on bacterial surface.

A, Bacteria staining by the anti-HA tag fluorescent antibody. **B,** Bacterial staining by the anti-VHH fluorescent antibody

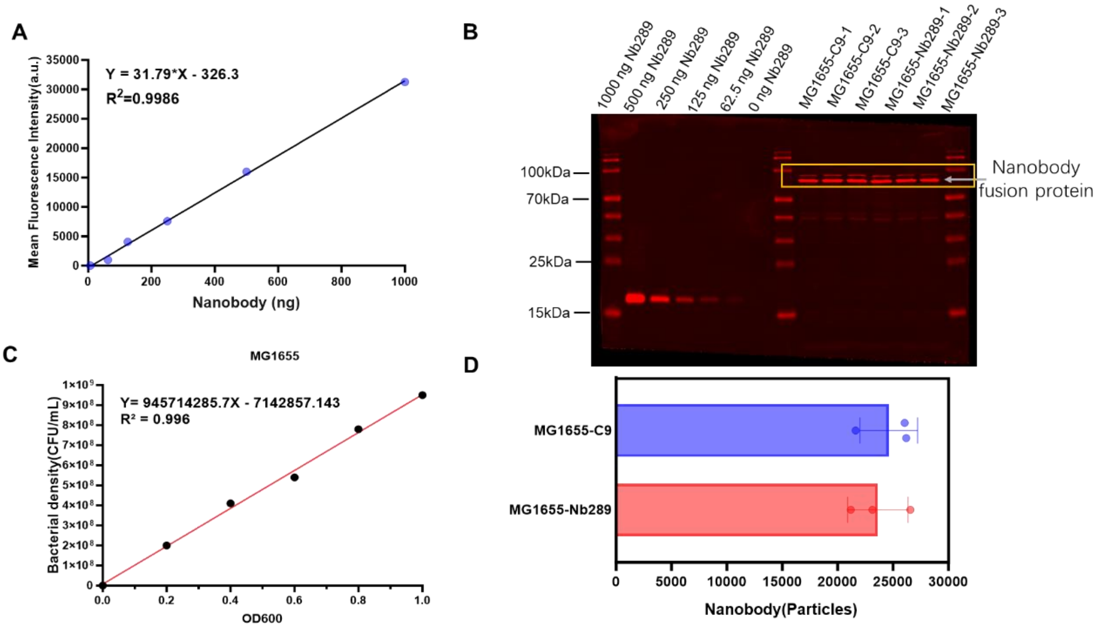

Figure S14. Quantitative analysis of the copy number of nanobody displayed onto a bacterium.

A, Standard curve of nanobody quantity and corresponding fluorescence intensity. **B**, Fluorescent western blot for nanobody standard curve (left) and engineered bacteria (right, yellow box). **C**, Standard curve of engineered bacteria established with OD600 absorbance values and the corresponding numbers of bacteria. **D**, The average copy numbers of nanobodies displayed on each MG1655-C9 and MG1655-Nb289 engineered bacterium.

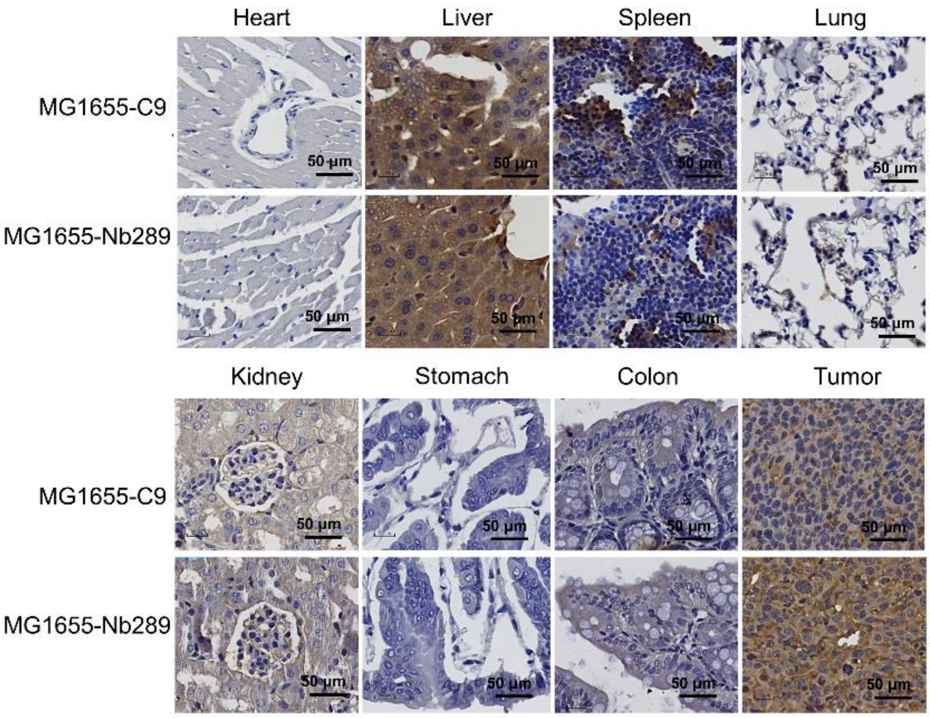

Figure S15. Immunohistochemical analysis of the distribution of nanobody-displayed bacteria in various tissues and organs.

Tissues were obtained from subcutaneously transplanted MKN45 gastric cancer mice 72h after engineered-bacteria injection. Various tissues were then subjected to the staining with an anti-VHH antibody. Scale bars, 50 μm.

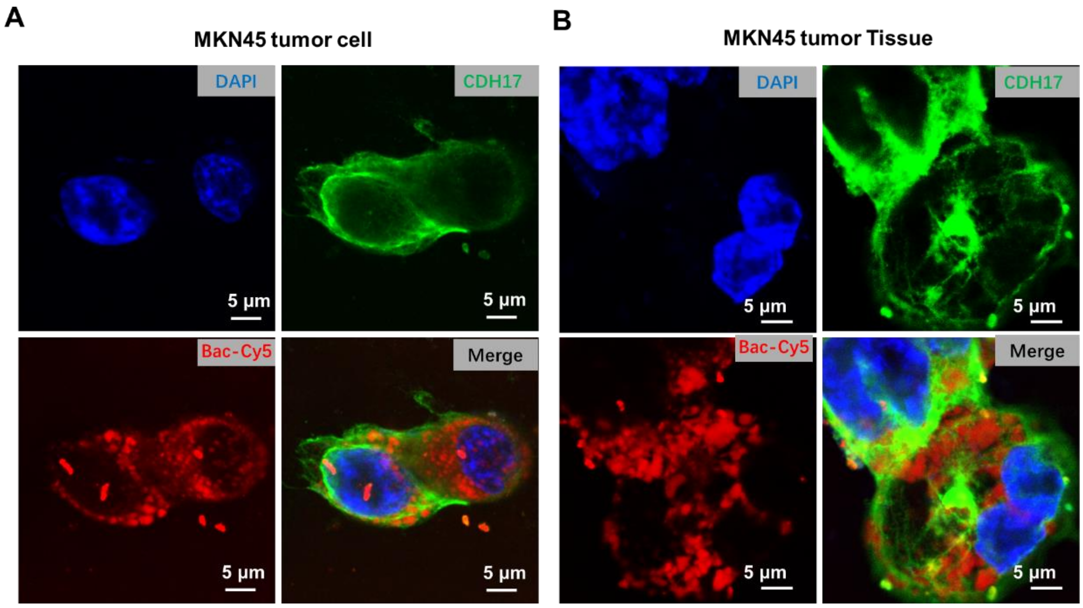

Figure S16. Internalization analysis of Nb289-engineered bacteria by tumor cells in vitro and in vivo.

A, Internalization of Nb289-engineered bacteria by CDH17-positive cancer cells in vitro after incubation with engineered bacteria. CDH17 was stained with a green fluorescent secondary antibody and bacteria were labeled with Cy5. **B,** Internalization of Nb289-engineered bacteria by CDH17-positive cancer cells in vivo after the administration of engineered bacteria in MKN45 tumor bearing mice. Tumor tissues were collected and sectioned after 24h circulation, and CDH17 was stained with a green fluorescent secondary antibody. Scale bars, 5 μm.

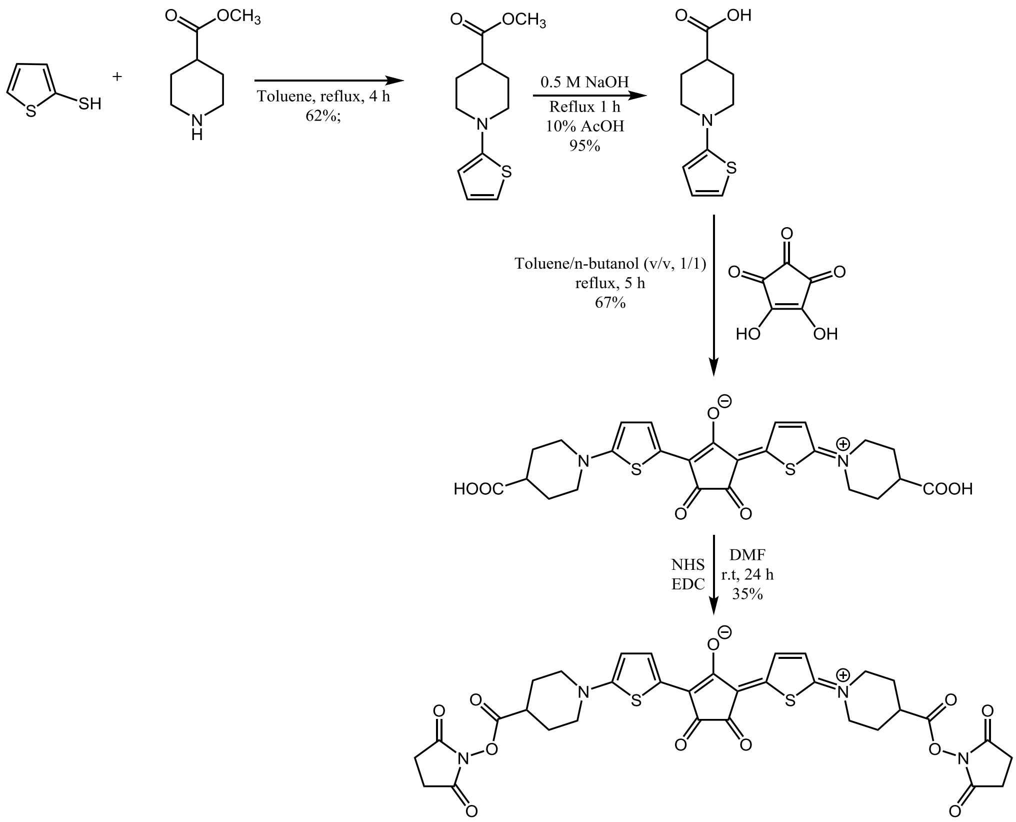

Figure S17. Flow chart of CR synthesis

Croconium dye was synthesized as described previously [1]. All chemicals were commercially available and used as received without further purification unless otherwise specified. Reaction progress was monitored by thin-layer chromatography (TLC) on silica plates (250 μmol/L thickness, bought from Qingdao Haiyang Chemical Co.) and spots were visualized by UV254 and 365 fluorescent indicators.

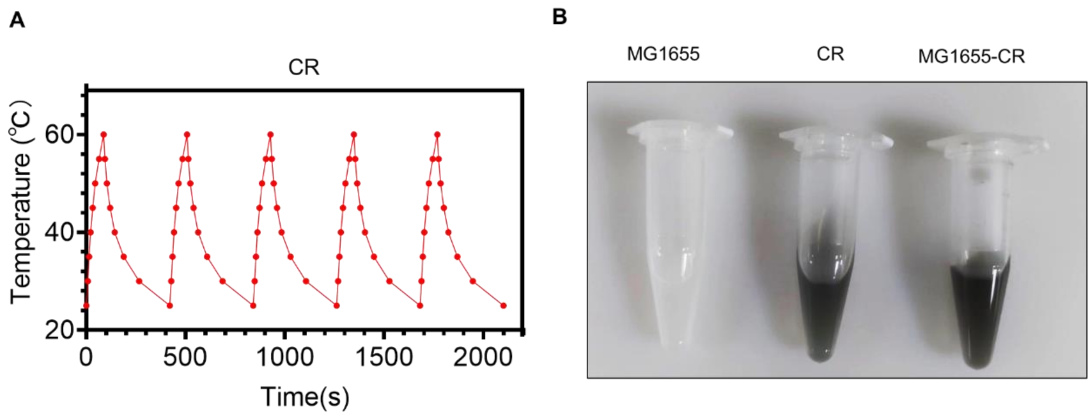

Figure S18. The conjugation of croconium (CR) photothermal molecules with engineered bacteria MG1655.

A, Photothermal stability analysis of CR photothermal molecules under five cycles of irradiations. **B,** The appearance of bacteria MG1655 conjugated with CR photothermal molecules.

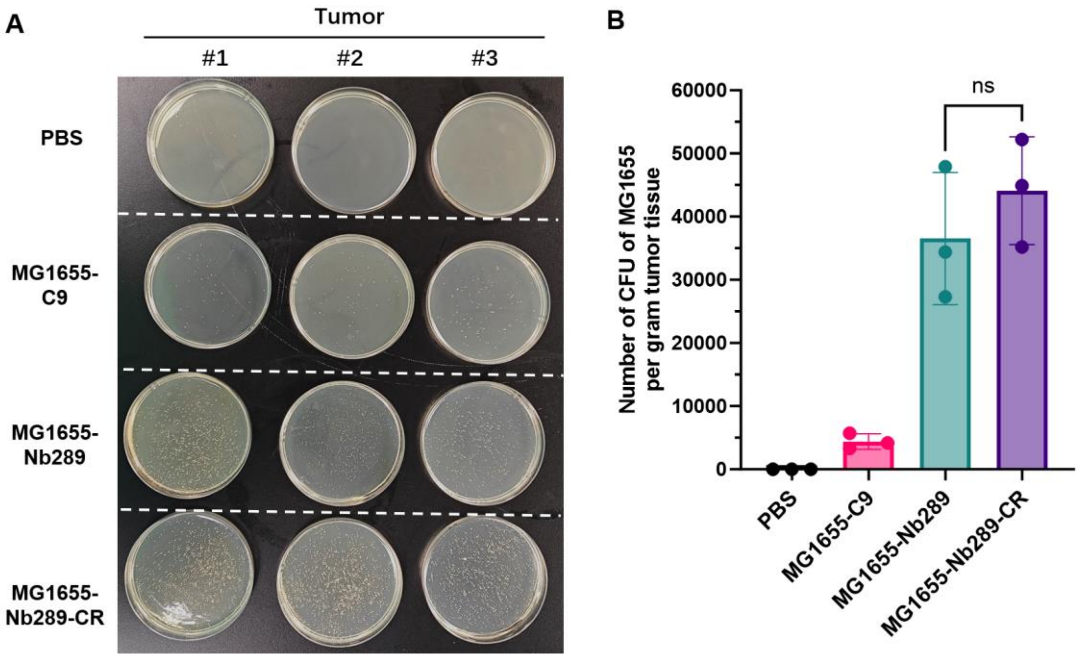

Figure S19. CR dye modification barely affects the homing ability of MG1655-Nb289 to CDH17-positive tumors

A. 5×10^6 CFU of MG1655-C9, MG1655-Nb289 or MG1655-Nb289-CR bacteria were injected into the MKN45 tumor-bearing mice via tail vein. Tumor tissues were weighed and homogenized for plate count of bacteria after 24 h circulation (n=3). **B.** Statistical analysis of the number of bacteria in tumors after various treatments. ns, no significance.

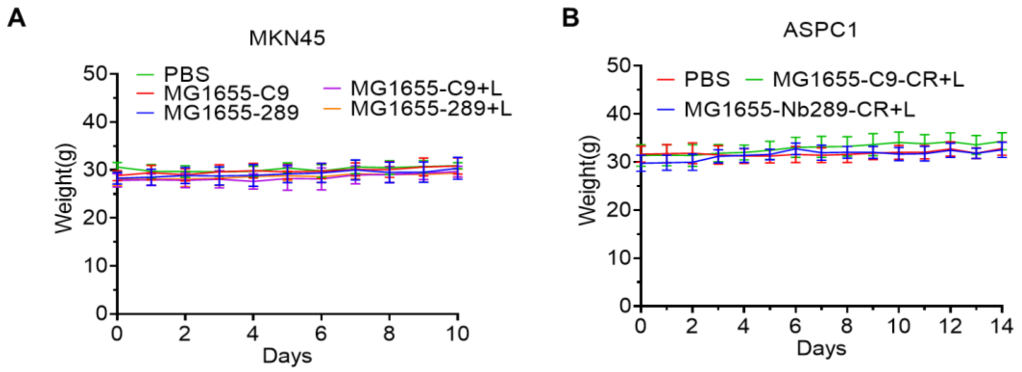

Figure S20. Mice body weight during biohybrid bacteria-mediated photothermal treatment.

A, Body weight changes in mice with subcutaneously transplanted MKN45 tumors treated with bacteria-mediated PTT (n=5); **B,** Body weight changes in mice with subcutaneously transplanted ASPC1 tumors treated with bacteria-mediated PTT (n=5).

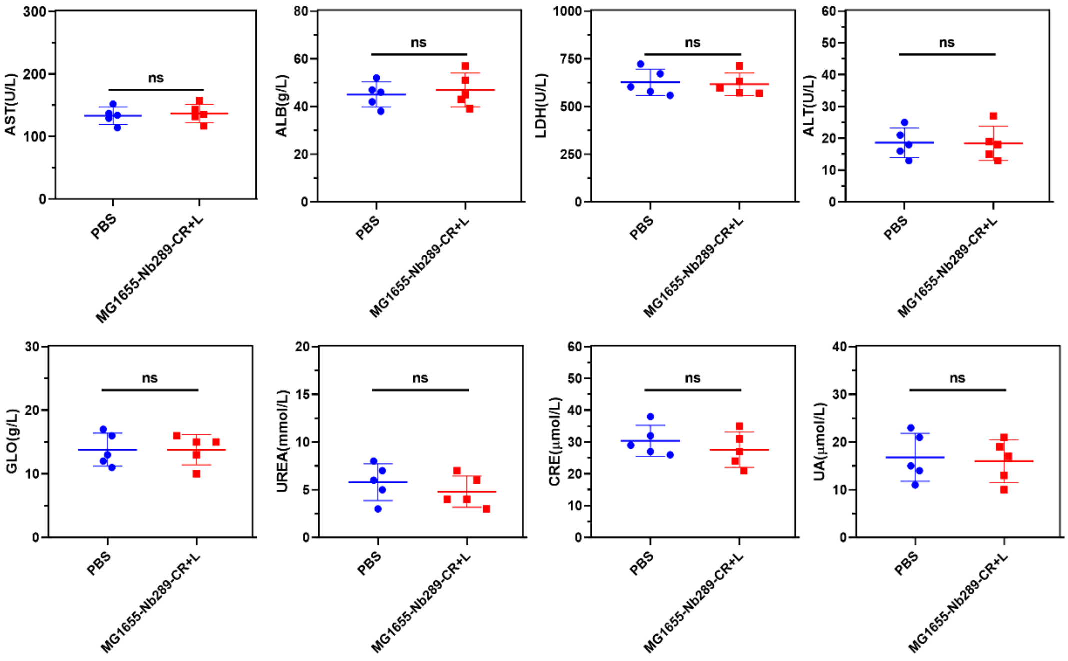

Figure S21. Blood biochemical analysis in mice after photothermal treatment with engineered bacteria (related to Figure 6B)

AST, aspartate aminotransferase; ALB. Albumin; LDH, Lactate Dehydrogenase; ALT, Alanine aminotransferase; GLO, Globulin; CRE, Creatinine; UA, Uric acid.

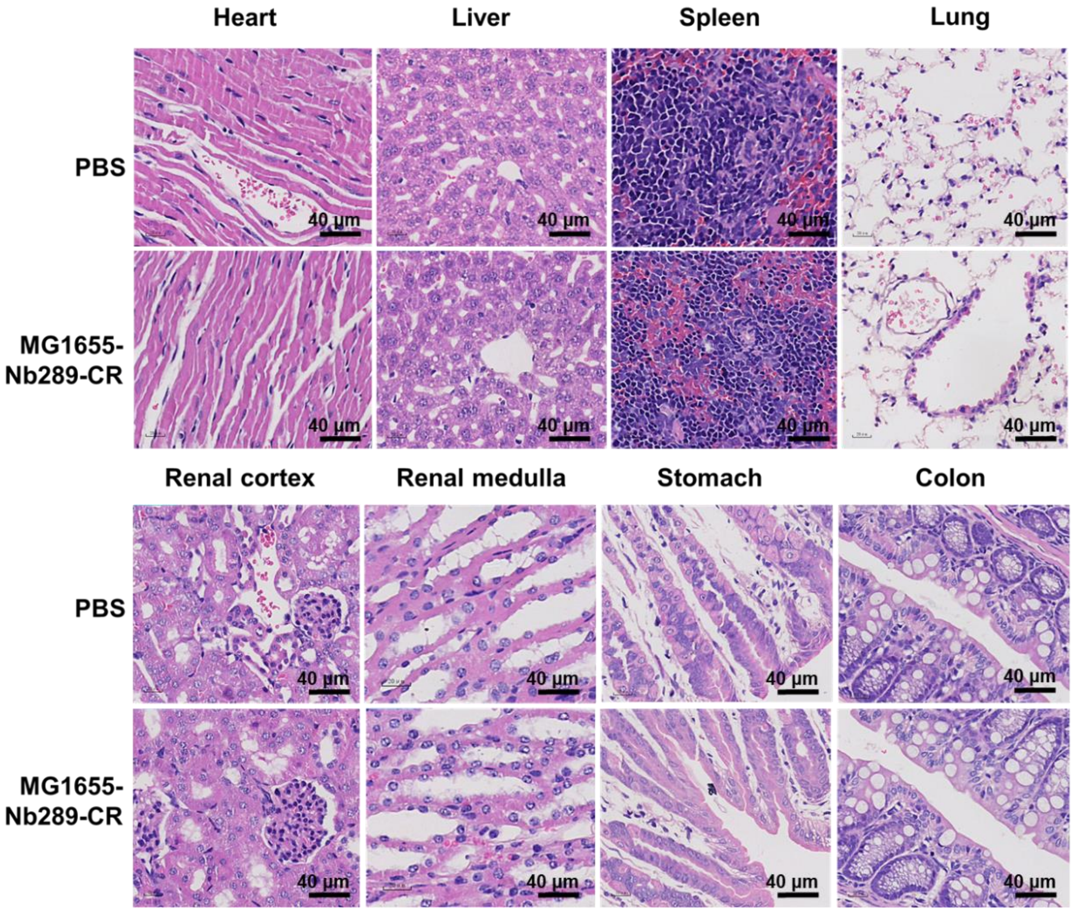

Figure S22. H&E staining for major organs from mice after photothermal treatment with engineered bacteria (related to Figure 6B)

Tissues were obtained from MKN45 gastric cancer-bearing mice 8 days after photothermal therapy. Scale bars: 40 μm.

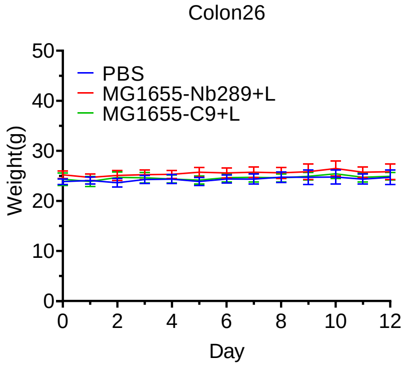

Figure S23. Body weight of mice received photothermal treatment with biohybrid bacteria in Colon26 tumor-bearing BALB/c mice (related to Figure 7C)

Body weight was monitored in Colon26 tumor-bearing mice treated with biohybrid bacteria-mediated PTT (n=5).

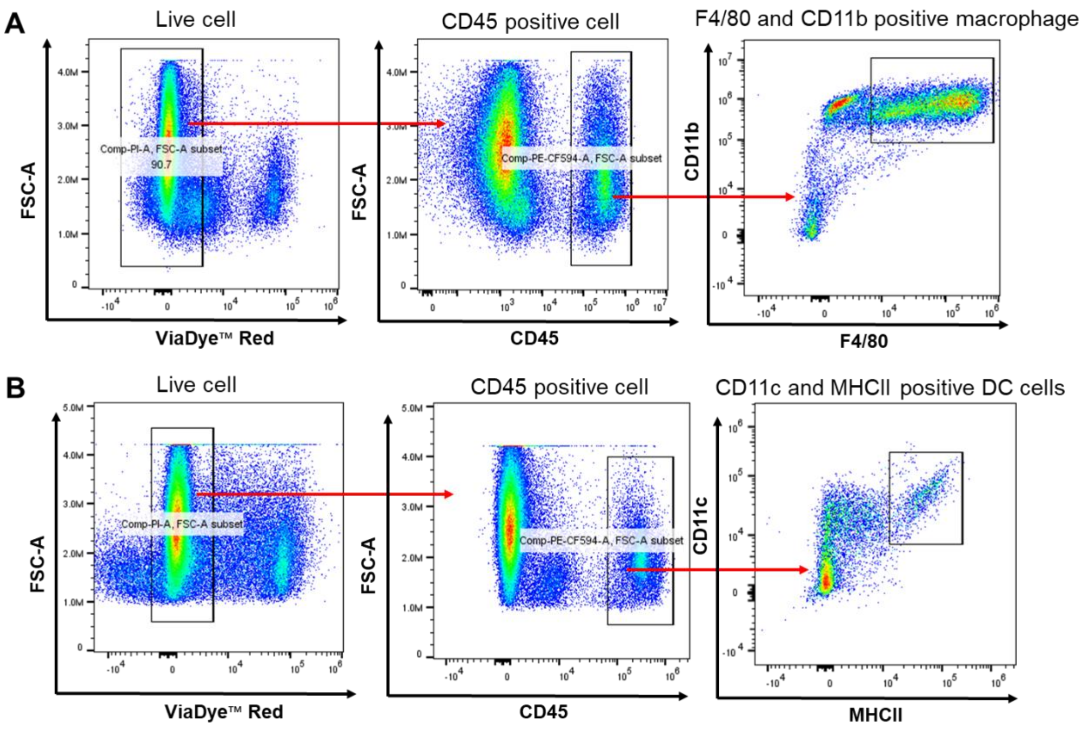

Figure S24. The gating strategy for infiltration of macrophages and DC cells in tumor tissue after photothermal treatment with engineered bacteria

A, The gating strategy for macrophages. **B,** The gating strategy for DCs.

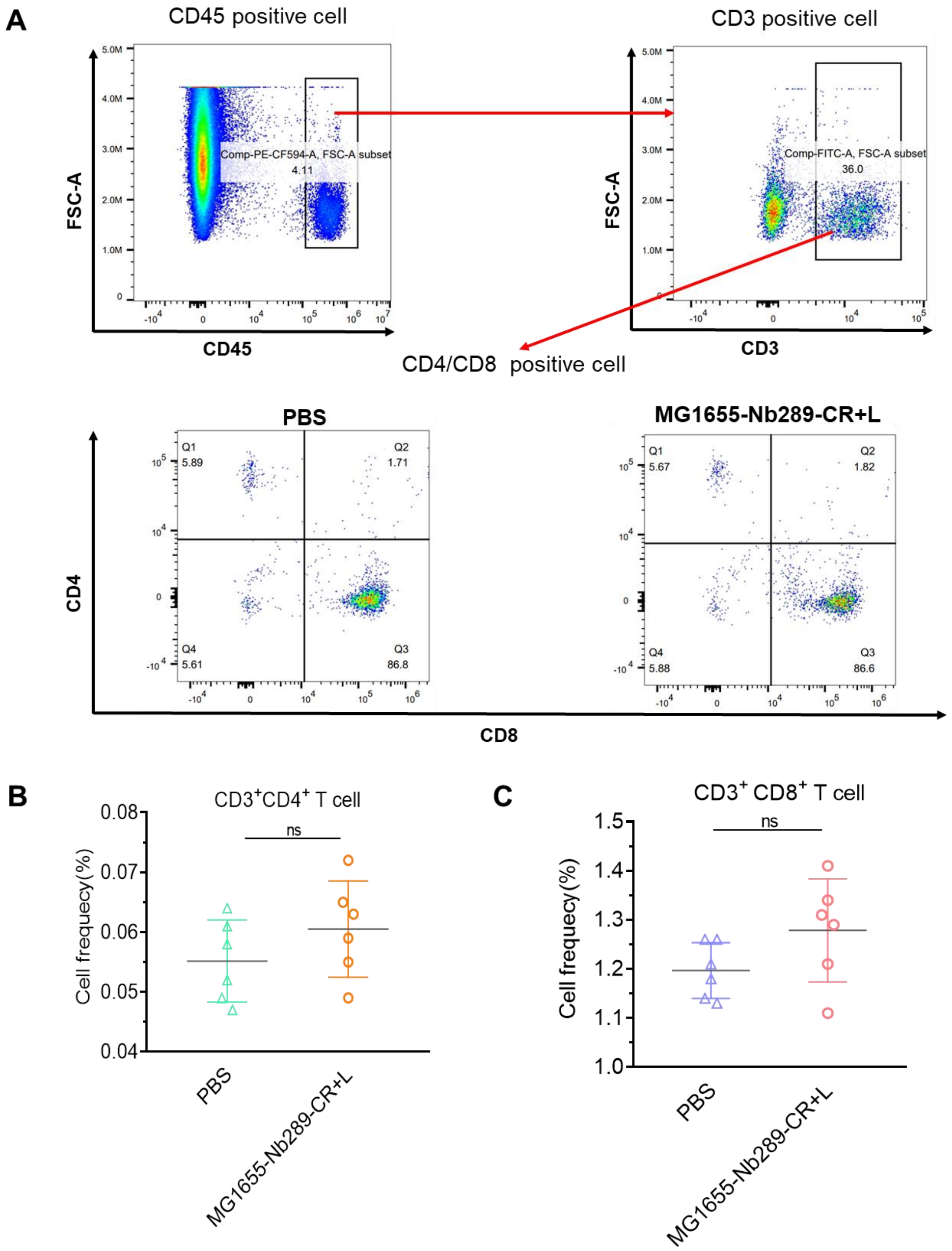

Figure S25. Flow cytometry analysis of infiltration of T cells in tumor tissues after photothermal treatment with engineered bacteria

A, The gating strategy for CD4 or CD8-positive T cells in tumors. **B,** Statistical analysis of $CD3^{+}CD4^{+}$ T cells (n=6). **C,** Statistical analysis of $CD3^{+}CD8^{+}$ T cells (n=6). ns, no significance.

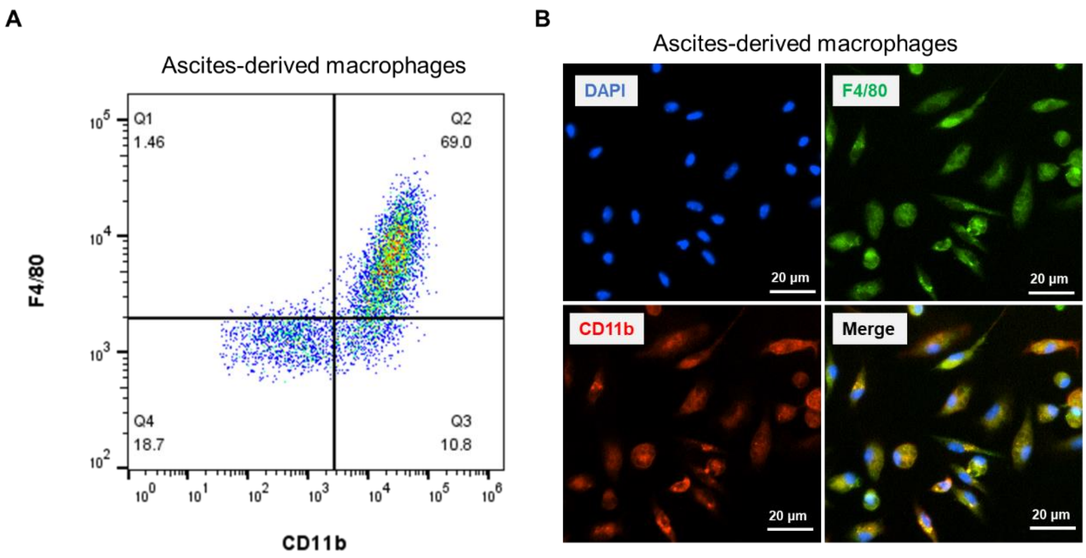

Figure S26. Identification of peritoneal cavity-derived macrophages.

A, Flow analysis perineal cavity-derived macrophages with F4/80 and CD11b antibodies; **B,** Immunofluorescence analysis of peritoneal cavity-derived macrophages with F4/80 and CD11b antibodies. Scale bars, 20 μm.

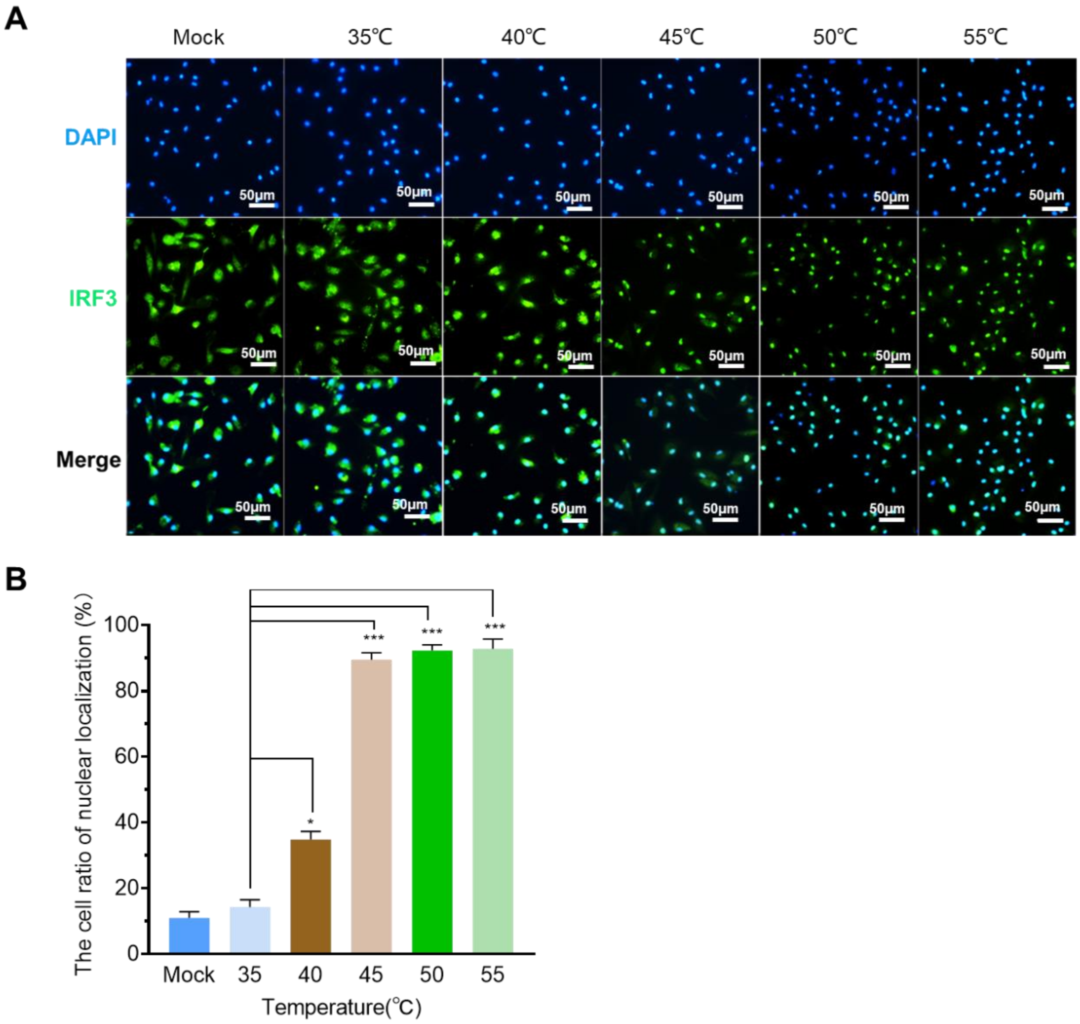

Figure S27. Immunofluorescence analysis of nuclear translocation of IRF3 in macrophages treated by supernatants of engineered bacteria.

Macrophages were treated with supernatants of engineered bacteria collected after heated with different temperatures and incubated for 24h. IRF3 was stained with the specific antibody against total IRF3. **A,** Immunofluorescence staining. **B,** The quantification of the percentage of nuclear expression of IRF3. Scale bars, 50 μm, $^{*}P < 0.05$, and $^{***}P < 0.001$..

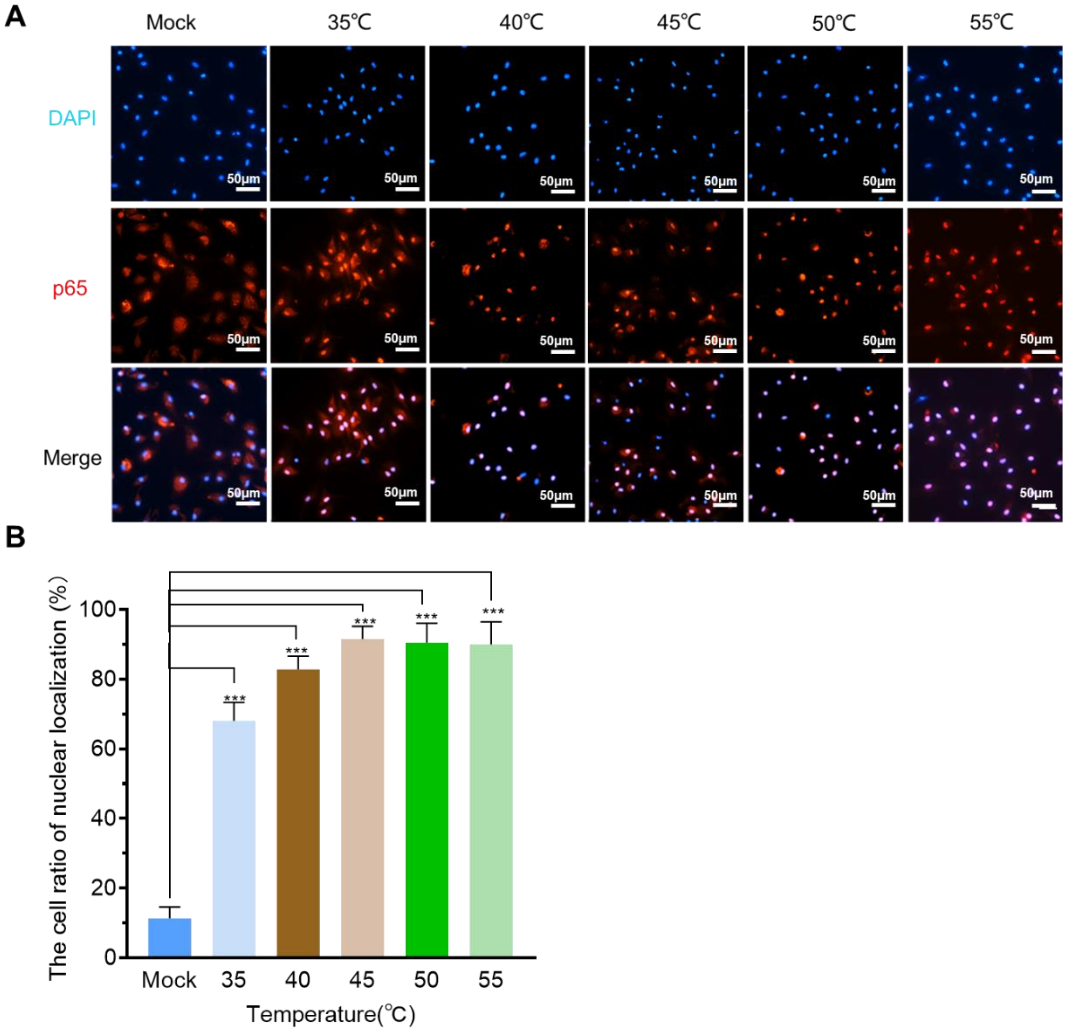

Figure S28. Immunofluorescence analysis of nuclear translocation of p65 in macrophages treated by supernatants of engineered bacteria.

Macrophages were treated with supernatants of engineered bacteria collected after heated with different temperatures and incubated for 24 h. p65 was stained with the specific antibody against total p65. **A,** Immunofluorescence staining. **B,** The quantification of the percentage of nuclear expression of p65. Scale bars, 50 μm,"***" means *P*<0.001.

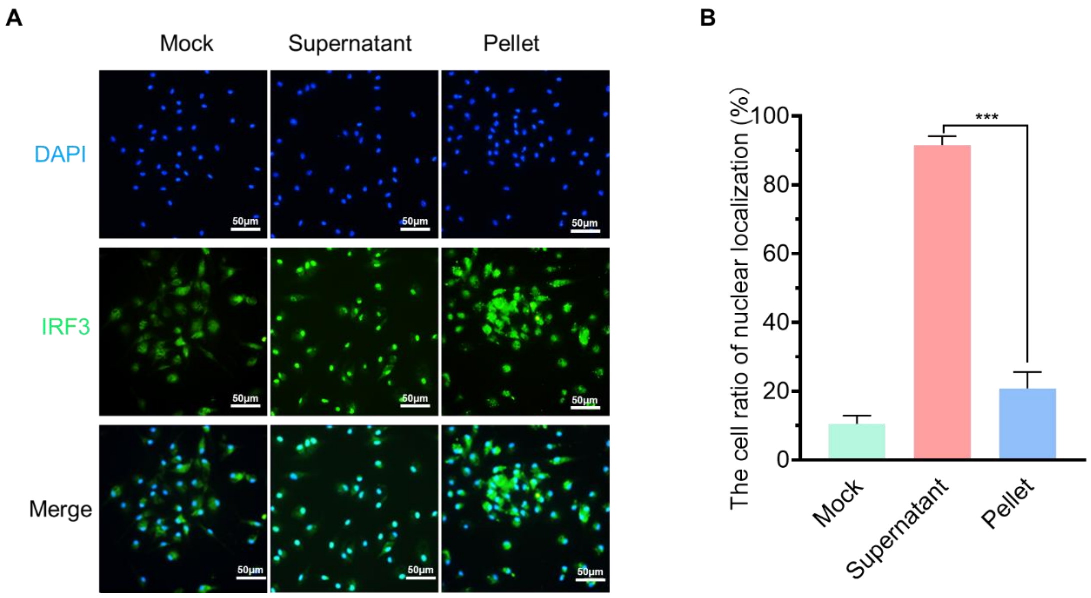

Figure S29. Immunofluorescence analysis of nuclear translocation of IRF3 in macrophages treated by supernatant or pellet isolated from engineered bacteria after heated with 45 °C.

Macrophages were treated with supernatant, or pellet of engineered bacteria collected after heated and incubated for 24 h. IRF3 was stained with the specific antibody against total IRF3. **A,** Immunofluorescence staining; **B,** The quantification of the percentage of nuclear expression of IRF3. Scale bars, 50 μm,"***" means $P<0.001$.

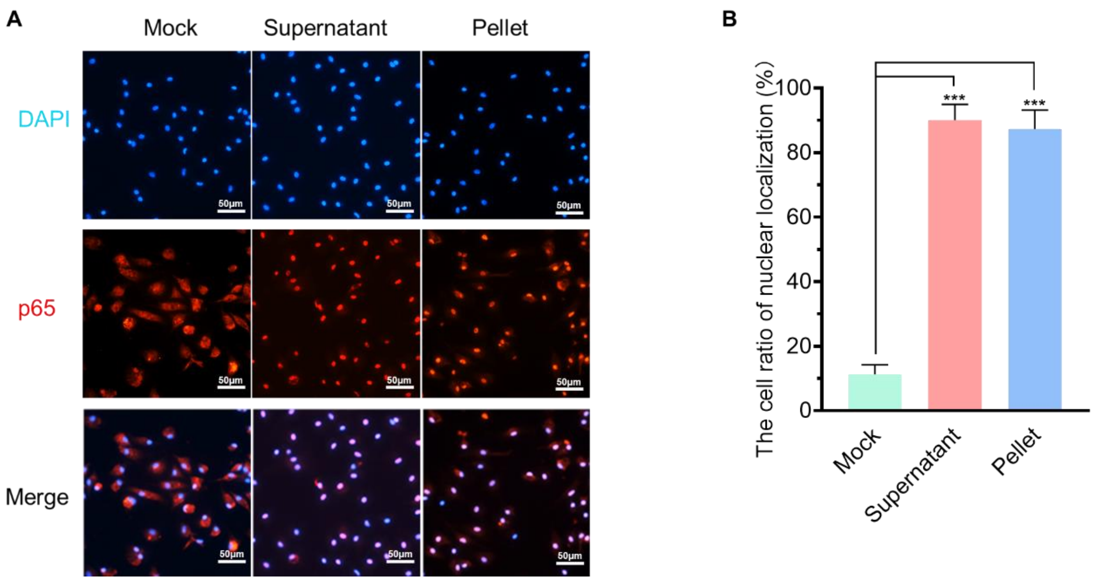

Figure S30. Immunofluorescence analysis of nuclear translocation of p65 in macrophages treated by supernatant or pellet of engineered bacteria.

Macrophages were treated with supernatant, or pellet of engineered bacteria collected after heated and incubated for 24 h. p65 was stained with the specific antibody against total p65. **A,** Immunofluorescence staining; **B,** The quantification of the percentage of nuclear expression of p65. Scale bars, 50 μm,"***" means P<0.001.

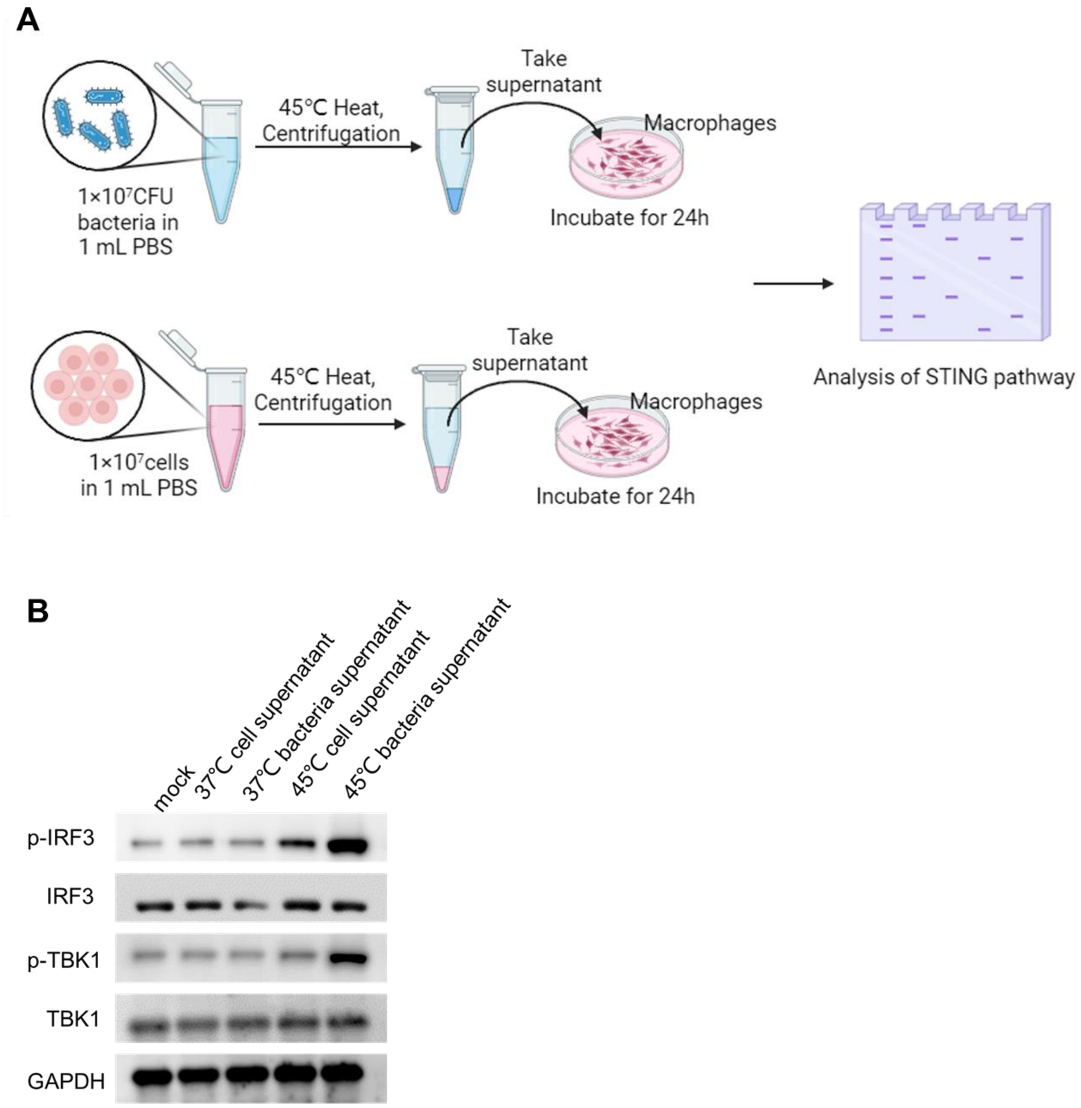

Figure S31. Comparison of the ability to STING pathway activation by DNA released from bacteria and cells after in vitro thermal treatment.

A, Schematic illustration of STING pathway activation assay in macrophages with DNA from heated cancer cells and bacteria. **B,** Western-blot detection of phosphorylation levels of IRF3 and TBK1, indicators for STING pathway activation, in macrophages after various treatments.

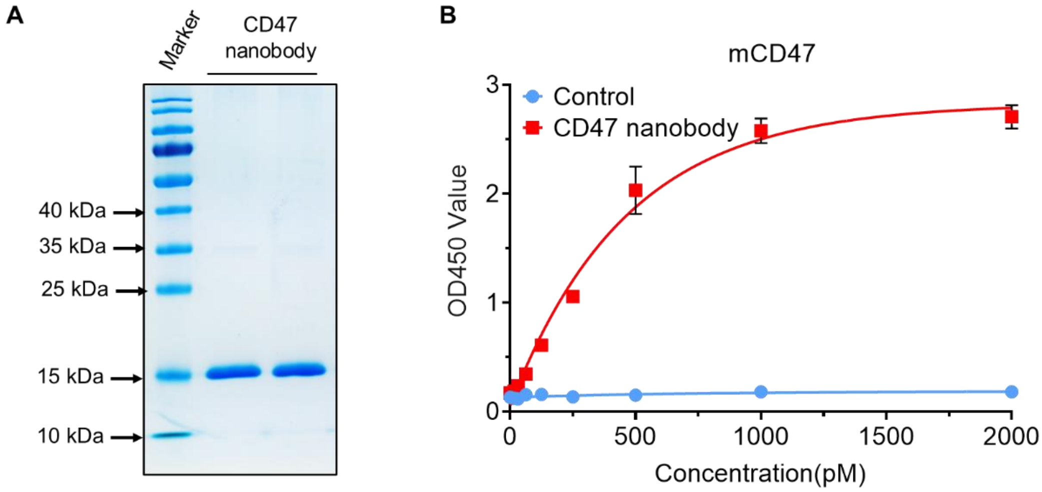

Figure S32. Purification and binding characterization of CD47 nanobody.

A, SDS-PAGE gel electrophoresis of CD47 nanobody stained with Coomassie Brilliant Blue.

B, ELISA analysis of CD47 nanobody binding activity to murine CD47(n=4).

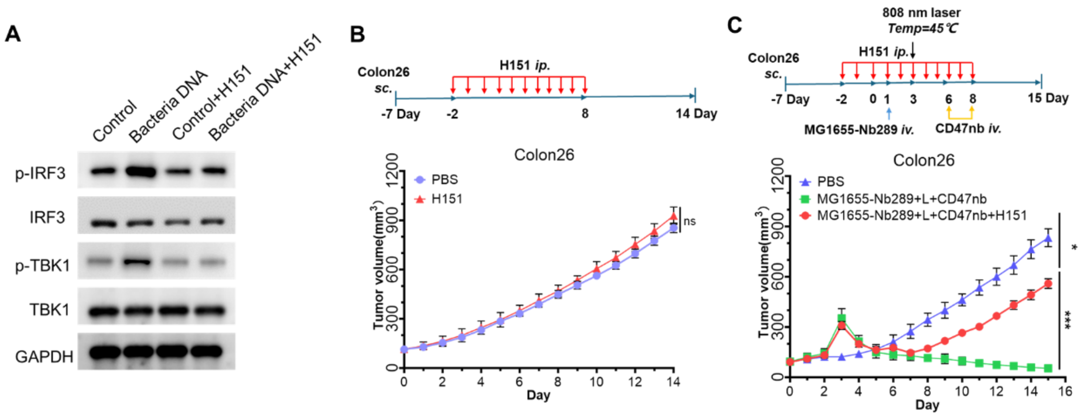

Figure S33. STING pathway inhibitor H151 reverses tumor inhibitory effect resulted from photothermally engineered bacteria therapy combined with CD47 nanobody.

A, STING pathway inhibition by H151 at the cellular level. **B,** No tumor growth repression for H151 alone in vivo (n=6). **C,** The effect of STING inhibitor H151 on tumor growth in mice receiving photothermally engineered bacteria therapy combined with CD47 nanobody (n=6). ***, P<0.001, *, P<0.05.

Table S1. Patient Characteristics of gastric cancer tissue microarray

			CDH17 IHC Staining		
**Variable**	**N**	**Overall**, N = 93[1]	**Negative**, N = 21[1]	**Positive**, N = 72[1]	**p-value**[2]
**Pathological type**	93				0.57
Adenocarcinoma		89 (96%)	21 (100%)	68 (94%)	
signet-ring cell carcinoma		4 (4.3%)	0 (0%)	4 (5.6%)	
**Gender**	93				0.087
female		30 (32%)	10 (48%)	20 (28%)	
male		63 (68%)	11 (52%)	52 (72%)	
**Age**	93				0.013
<or=60		40 (43%)	14 (67%)	26 (36%)	
>60		53 (57%)	7 (33%)	46 (64%)	
**Tumor location**	93				>0.99
curvature of the stomach sinus		6 (6.5%)	1 (4.8%)	5 (6.9%)	
gastric antrum pylorus		3 (3.2%)	0 (0%)	3 (4.2%)	
gastric sinus		81 (87%)	20 (95%)	61 (85%)	
greater curvature of the antrum		3 (3.2%)	0 (0%)	3 (4.2%)	
**pathological grade**	93				0.006
I		30 (32%)	12 (57%)	18 (25%)	
II		31 (33%)	7 (33%)	24 (33%)	
III		32 (34%)	2 (9.5%)	30 (42%)	
**Lymph node invasion**	93				0.17
>or=1		69 (74%)	18 (86%)	51 (71%)	
0		24 (26%)	3 (14%)	21 (29%)	

[1]Median (IQR) or Frequency (%)

			CDH17 IHC Staining		
**Variable**	**N**	**Overall**, N = 93[1]	**Negative**, N = 21[1]	**Positive**, N = 72[1]	**p-value**[2]

[2]Fisher's exact test; Pearson's Chi-squared test

Table S2. Patient characteristics of pancreatic cancer tissue microarray

			CDH17 IHC Staining		
**Variable**	**N**	**Overall**, N = 140[1]	**Negative**, N = 42[1]	**Positive**, N = 98[1]	**p-value**[2]
**Pathological type**	140				0.009
Adenocarcinoma		122 (87%)	42 (100%)	80 (82%)	
Adenosine squamous carcinoma		7 (5.0%)	0 (0%)	7 (7.1%)	
mucinous adenocarcinoma		11 (7.9%)	0 (0%)	11 (11%)	
**Gender**	140				0.41
female		47 (34%)	12 (29%)	35 (36%)	
male		93 (66%)	30 (71%)	63 (64%)	
**Age**	140				0.91
<or=60		69 (49%)	21 (50%)	48 (49%)	
>60		71 (51%)	21 (50%)	50 (51%)	
**pathological grade**	140				<0.001
I		42 (30%)	23 (55%)	19 (19%)	

		CDH17 IHC Staining			
**Variable**	**N**	**Overall**, N = 140[1]	**Negative**, N = 42[1]	**Positive**, N = 98[1]	**p-value**[2]
II		50 (36%)	13 (31%)	37 (38%)	
III		48 (34%)	6 (14%)	42 (43%)	
**Tumor location**	140				0.020
body of pancreas		7 (5.0%)	1 (2.4%)	6 (6.1%)	
head of the pancreas		121 (86%)	41 (98%)	80 (82%)	
tail of pancreas		12 (8.6%)	0 (0%)	12 (12%)	
**Lymph node invasion**	140				0.19
>or=1		18 (13%)	3 (7.1%)	15 (15%)	
0		122 (87%)	39 (93%)	83 (85%)	

[1]Median (IQR) or Frequency (%)

[2]Fisher's exact test; Pearson's Chi-squared test

Table S3. Patient characteristics of colon cancer tissue microarray

		CDH17 IHC Staining			
**Variable**	**N**	**Overall**, N = 93[1]	**Negative**, N = 21[1]	**Positive**, N = 72[1]	**p-value**[2]
**Pathological type**	93				0.57
Adenocarcinoma		89 (96%)	21 (100%)	68 (94%)	
signet-ring cell carcinoma		4 (4.3%)	0 (0%)	4 (5.6%)	
**Gender**	93				0.087

		CDH17 IHC Staining			
**Variable**	**N**	**Overall**, N = 93[1]	**Negative**, N = 21[1]	**Positive**, N = 72[1]	**p-value**[2]
female		30 (32%)	10 (48%)	20 (28%)	
male		63 (68%)	11 (52%)	52 (72%)	
**Age**	93				0.013
<or=60		40 (43%)	14 (67%)	26 (36%)	
>60		53 (57%)	7 (33%)	46 (64%)	
**Tumor location**	93				>0.99
curvature of the stomach sinus		6 (6.5%)	1 (4.8%)	5 (6.9%)	
gastric antrum pylorus		3 (3.2%)	0 (0%)	3 (4.2%)	
gastric sinus		81 (87%)	20 (95%)	61 (85%)	
greater curvature of the antrum		3 (3.2%)	0 (0%)	3 (4.2%)	
**pathological grade**	93				0.006
I		30 (32%)	12 (57%)	18 (25%)	
II		31 (33%)	7 (33%)	24 (33%)	
III		32 (34%)	2 (9.5%)	30 (42%)	
**Lymph node invasion**	93				0.003
>or=1		57 (61%)	7 (33%)	50 (69%)	
0		36 (39%)	14 (67%)	22 (31%)	

[1]Median (IQR) or Frequency (%)

[2]Fisher's exact test; Pearson's Chi-squared test

Table S4. Primers applied in research

No.	name	sequence (from 5' to 3')
1	m_β-actin-F	GGCTGTATTCCCCTCCATCG
2	m_β-actin-R	CCAGTTGGTAACAATGCCATGT
3	m_CXCL9-F	TCCTTTTGGGCATCATCTTCC
4	m_CXCL9-R	TTTGTAGTGGATCGTGCCTCG
5	m_CXCL10-F	CCAAGTGCTGCCGTCATTTTC
6	m_CXCL10-R	GGCTCGCAGGGATGATTTCAA
7	m_CCL5-F	GCTGCTTTGCCTACCTCTCC
8	m_CCL5-R	TCGAGTGACAAACACGACTGC
9	m_IFNB1-F	CAGCTCCAAGAAAGGACGAAC
10	m_IFNB1-R	GGCAGTGTAACTCTTCTGCAT
11	m_IFNG-F	ATGAACGCTACACACTGCATC
12	m_IFNG-R	CCATCCTTTTGCCAGTTCCTC
13	m_IFIH1-F	AGATCAACACCTGTGGTAACACC
14	m_IFIH1-R	CTCTAGGGCCTCCACGAACA
15	m_IFIT1-F	CTGAGATGTCACTTCACATGGAA
16	m_IFIT1-R	GTGCATCCCCAATGGGTTCT
17	m_IFIT2-F	AGTACAACGAGTAAGGAGTCACT
18	m_IFIT2-R	AGGCCAGTATGTTGCACATGG
19	m_IL6-F	CCAAGAGGTGAGTGCTTCCC
20	m_IL6-R	CTGTTGTTCAGACTCTCTCCCT
21	m_ISG15-F	GGTGTCCGTGACTAACTCCAT
22	m_ISG15-R	TGGAAAGGGTAAGACCGTCCT
23	m_MX1-F	GACCATAGGGGTCTTGACCAA
24	m_MX1-R	AGACTTGCTCTTTCTGAAAAGCC
25	m_β-TNF1A-F	GGTCCCCAAAGGGATGAGAAGT
26	m_β-TNF1A-R	TTGCTACGACGTGGGCTACA
